# Supplementary material for: Estimating the cost impact of atrial fibrillation using a prospective cohort study and population-based controls
Source: BMJ Open. 2023 Sep 14;13(9):e072080. doi: 10.1136/bmjopen-2023-072080 (PMC10503354; doi:10.1136/bmjopen-2023-072080)
Supplement: Supplementary data [file bmjopen-2023-072080supp001.pdf]

Table S1. Sample size determination for the population-based control sample

In the absence of exact solutions for the determination of the required size of the population-based, non-AF control sample, we tried to estimate a plausible magnitude based on published cost studies. Our study aim was to compare the costs of prevalent atrial fibrillation (AF) patients with the costs of controls not having AF. In the absence of published comparisons of this type, we used the AF-attributable versus non-AF-attributable costs of AF patients as a fallback. We found that mean attributable costs may differ by roughly 0.3 standard deviations from the mean of non-attributable costs [1, 2, 3]. Based on the results of Turakhia [4], an expected minimum effect size (Cohen's d) of the cost of AF would be approximately 0.1. Given the possibility of such a small effect size and to be on the safe side we assumed a 50% smaller effect, i.e. Cohen's d of 0.05.

AF patients were planned to be compared to controls differing in several dimensions, and a variety of sub-analyses were planned to be performed to characterize the cost impact of AF. To mimic the impact of this situation on the required size of the control sample, a Bonferroni correction for multiple comparisons was assumed, with an estimated number of 15 hypothesis: checking for divergences in gender, age, accumulation of costs over time in different subgroups, various types of costs etc.

With a standard statistical power function and assuming the parameter values and corrections explained above (Cohen's d = 0.05, number of hypotheses = 15), a sample size of 17'000 valid controls was estimated to be required to obtain a 95% statistical power and a 5% false positive risk. This became the planned size of the non-AF control sample. Considering that some otherwise eligible people would have AF, the size of the full reference sample was inflated to the point where 17'000 non-AF controls were reached.

[1] Jönsson et al. 2010. Cost of Illness and drivers of Cost in Atrial Fibrillation in Sweden and Germany, Applied Health Economics and Health Policy, 8, 317-325, DOI: 10.2165/11319880-000000000-00000

[2] Brüggenjürgen et al. 2007, The Impact of Atrial Fibrillation on the Cost of Stroke: the Berlin Acute Stroke Study, Value in Health, 10:2, 137-143, DOI: 10.1111/j.1524-4733.2006.00160.x

[3] Wodchis et al. 2012. A Review of the Cost of Atrial Fibrillation, Value in Health, 15:2, 240-248, DOI: https://doi.org/10.1016/j.jval.2011.09.009

[4] Turakhia et al. 2015. Economic Burden of Undiagnosed Nonvalvular Atrial Fibrillation in the United States, Am J Cariol, 116:5, 733-739, DOI: 10.1016/j.amjcard.2015.05.045.

Table S2. Algorithm classifying the population-based reference sample as "AF likely" and "AF possible". Individuals with none of the listed criteria present were classified as "AF not obvious" and considered as controls.

code		allocation	
		AF likely	AF possible
ICD10	**I48.0** Vorhofflimmern, paroxysmal	1	
ICD10	**I48.1** Vorhofflimmern, persistierend	1	
ICD10	**I48.2** Vorhofflimmern, permanent	1	
ICD10	**I48.3** Vorhofflattern, typisch	1	
ICD10	**I48.4** Vorhofflattern, atypisch	1	
ICD10	**I48.9** Vorhofflimmern und Vorhofflattern, nicht näher bezeichnet	1	
ICD10	**I49.8** Sonstige näher bezeichnete kardiale Arrhythmien		1
ICD10	**I49.9** Kardiale Arrhythmie, nicht näher bezeichnet		1
DRG	**F50A** Ablative Massnahmen bei Tachyarrhythmie mit bestimmter Ablation und komplexem Eingriff, Alter < 16 Jahre		1
DRG	**F50D** Ablative Massnahmen bei Tachyarrhythmie, Alter > 15 Jahre		1
ICD10 + DRG	ICD **I48 +** DRG **F50A**	1	
ICD10 + DRG	ICD **I48 +** DRG **F50D**	1	
CHOP	**Z37.34.24** Lokalisationen bei Ablationsverfahren bei Tachyarrhythmien	1	
CHOP	**Z99.61** Vorhofskardioversion	1	
CHOP	**Z99.62** Externe Kardioversion	1	
Tarmed	**17.1510** Kardioversion bei Vorhofflimmern/Vorhofflattern, als alleinige Leistung	1	
ATC	**C01BD07** Dronedarone (Multaq)	1	

Notes: Abbreviations: AF: atrial fibrillation, ATC: anatomical therapeutic chemical classification, CHOP: Swiss invasive medical procedures catalogue, DRG: diagnosis related group, ICD10: international classification of diseases (10th revision).

Figure S1. Trajectories of monthly costs and Kaplan-Meier curves by cohort.

A)

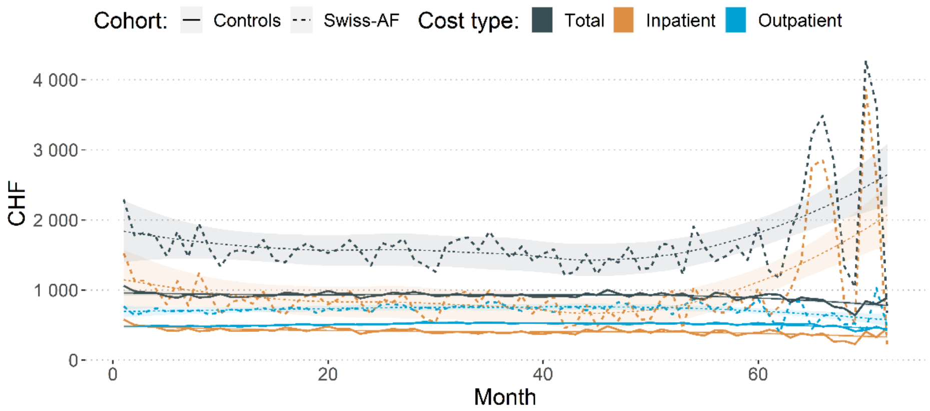

B)

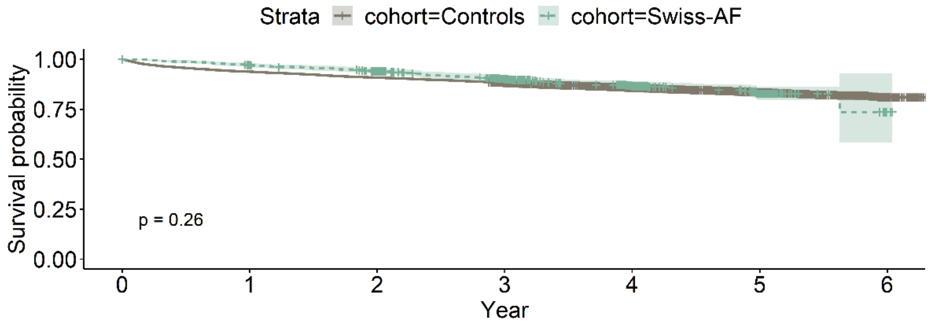

C) N at risk

Number at risk

Strata	0	1	2	3	4	5	6
cohort=Controls	16556	15532	15041	14279	8714	4299	385
cohort=Swiss-AF	1024	982	920	706	375	136	2

Year

D) deaths

Cumulative number of events

Strata	0	1	2	3	4	5	6
cohort=Controls	5	1026	1515	1939	2271	2439	2504
cohort=Swiss-AF	0	29	60	96	117	126	127

Year

E) loss to FU

Cumulative number of events

Strata	0	1	2	3	4	5	6
cohort=Controls	0	0	0	0	0	0	0
cohort=Swiss-AF	7	14	23	28	34	37	39

Year

F) admin. censoring

Cumulative number of events

Strata	0	1	2	3	4	5	6
cohort=Controls	0	0	0	357	5572	9844	13675
cohort=Swiss-AF	0	0	26	212	505	735	856

Year

Notes: median (interquartile range IQR) follow-up: Swiss-AF 3.41 (1.08) years, controls 4.10 (1.72) years; total patient-years of follow-up: SAF 3 571.24, cohort 66 068.24.

Table S3. Observed annual costs in CHF by cost component and cohort.

	Swiss-AF		***Controls***	
Cost component	*Median [IQR]*	*Mean (SD)*	*Median [IQR]*	*Mean (SD)*
Total	4 518 [825, 11 771]	19 037 (59 998)	2 135 [0, 7 473]	11 192 (38 939)
Total inpatient	0 [0, 0]	10 235 (56 327)	0 [0, 0]	5 077 (34 925)
Total outpatient drugs	508 [0, 29 56]	2 495 (7 382)	235 [0, 1 781]	1 984 (7 852)
Total outpatient without drugs	2 282 [59, 7 225]	6 307 (13 154)	801 [0, 4 310]	4 131 (10 260)
Total AF-adj.	400 [0, 3 213]	5 679 (36 135)	*NA*	*NA*
Total AF-adj. inpatient	0 [0, 0]	3 458 (35 188)	*NA*	*NA*
Total AF-adj. outpatient drugs	0 [0, 250]	591 (1 392)	*NA*	*NA*
Total AF-adj. outpatient without drugs	0 [0, 1 251]	1 630 (6 899)	*NA*	*NA*
AF-adjudication:				
Total AF treatment	226 [0, 2 773]	4 078 (2 8640)	*NA*	*NA*
Total stroke or TIA	0 [0, 0]	174 (9124)	*NA*	*NA*
Total bleeding	0 [0, 0]	696 (17462)	*NA*	*NA*
Total fall	0 [0, 0]	237 (4434)	*NA*	*NA*
Total heart failure	0 [0, 0]	494 (8469)	*NA*	*NA*

Abbreviations: adj.: adjudicated, AF: atrial fibrillation, IQR: interquartile range, PCG: pharmaceutical cost groups, SD: standard deviation, SMD: standardized mean difference.

Figure S2. Boxplot distribution of mean annual costs by cost outcome and cohort.

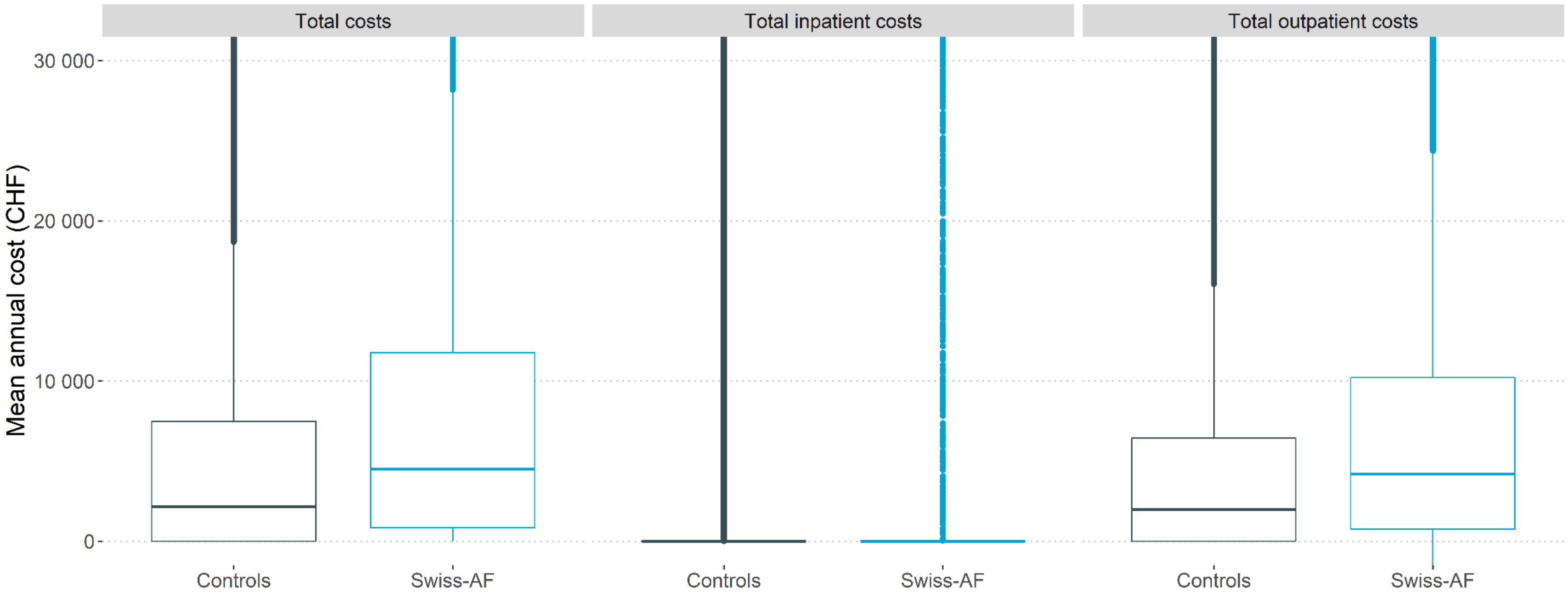

Table S4. Regression results from GLM-based two part modelling.

	**Total costs**		**Outpatient costs**		**Inpatient costs**	
	Odds ratio (Logistic part)	*Marginal effect (GLM part)*	*Odds ratio (Logistic part)*	*Marginal effect (GLM part)*	*Odds ratio (Logistic part)*	*Marginal effect (GLM part)*
Cohort: Swiss-AF	1.5 [1.46, 1.54]	6 374 [5 609, 7 139]	1.46 [1.42, 1.5]	1 299 [1 097, 1 501]	1.13 [1.08, 1.17]	35 154 [28 827, 41 481]
Month	1.00 [1.00, 1.00]	29 [20, 38]	1.00 [1.00, 1.00]	35 [33, 38]	1.00 [1.00, 1.00]	- 335 [- 408, -262]
Age	1.03 [1.03, 1.03]	242 [223, 260]	1.03 [1.02, 1.03]	36 [30, 41]	1.09 [1.09, 1.09]	- 3 075 [- 3 244, - 2 906]
Sex: Male	0.92 [0.91, 0.93]	3 254 [2 963, 3 545]	0.92 [0.91, 0.93]	1 485 [1 393, 1 577]	1.05 [1.02, 1.07]	10 449 [8 031, 12 866]
PCG acid related disorders	1.69 [1.66, 1.72]	2 610 [2 231, 2 989]	1.66 [1.63, 1.69]	1 487 [1 367, 1 606]	1.30 [1.27, 1.34]	- 3 545 [- 6 209, - 882]
PCG bone diseases	1.94 [1.87, 2.01]	5 278 [4 418, 6 138]	1.91 [1.84, 1.98]	4 517 [4 214, 4 821]	0.97 [0.93, 1.02]	18 455 [12 539, 24 372]
PCG cancer	2.12 [2.03, 2.21]	16 094 [14 613, 17 575]	2.09 [2, 2.18]	12 834 [12 269, 13 399]	1.22 [1.16, 1.29]	18 812 [11 626, 25 999]
PCG cardio	1.89 [1.87, 1.91]	- 402 [- 738, - 66]	1.90 [1.87, 1.92]	317 [214, 419]	0.98 [0.96, 1.01]	8 045 [5 312, 10 779]
PCG dementia	2.05 [1.98, 2.13]	1 819 [1 166, 2 471]	1.93 [1.87, 2]	949 [745, 1 154]	2.14 [2.07, 2.22]	- 26 586 [- 29 160, - 24 012]
PCG diabetes	1.67 [1.64, 1.7]	3 790 [3 355, 4 225]	1.66 [1.63, 1.69]	2 220 [2 083, 2 358]	1.25 [1.21, 1.28]	5 212 [1 986, 8 439]
PCG epilepsy	2.26 [2.18, 2.34]	5 403 [4 703, 6 103]	2.19 [2.12, 2.27]	2 636 [2 421, 2 851]	1.58 [1.53, 1.63]	- 3 375 [- 6 723, - 28]
PCG glaucoma	1.56	- 493	1.56	638	0.87	- 1 171

	[1.53, 1.59]	[- 902, - 83]	[1.53, 1.59]	[500, 776]	[0.85, 0.9]	[- 4 610, 2 268]
PCG gout	1.35<br>[1.32, 1.39]	1 296<br>[713, 1 878]	1.36<br>[1.32, 1.39]	509<br>[329, 688]	1.02<br>[0.98, 1.06]	7 684<br>[27 98, 12 571]
PCG hyperlipidemia	1.32<br>[1.3, 1.33]	- 1 142<br>[- 1 437, - 847]	1.32<br>[1.3, 1.33]	- 275<br>[- 368, - 182]	0.86<br>[0.84, 0.88]	5 201<br>[2 606, 7 796]
PCG iron deficiency	1.57<br>[1.51, 1.64]	5 284<br>[4 401, 6 167]	1.56<br>[1.5, 1.62]	3 695<br>[3 401, 3 989]	1.20<br>[1.15, 1.25]	2 373<br>[- 2 429, 7 176]
PCG pain	1.47<br>[1.44, 1.5]	6 097<br>[5 628, 6 567]	1.45<br>[1.42, 1.49]	2 326<br>[2 186, 2 465]	1.82<br>[1.78, 1.87]	- 3 500<br>[- 6 189, - 812]
PCG psychiatric	2.19<br>[2.15, 2.24]	1 909<br>[1 534, 2285]	2.17<br>[2.12, 2.21]	1 048<br>[930, 1 167]	1.60<br>[1.56, 1.64]	- 15 150<br>[- 17 606, - 12 693]
PCG antipsychotic	2.92<br>[2.78, 3.06]	9 281<br>[8278, 1 0285]	2.50<br>[2.39, 2.62]	1 347<br>[1 108, 1 586]	5.98<br>[5.8, 6.17]	- 39 595<br>[- 41 844, - 37 345]
PCG respiratory	1.67<br>[1.64, 1.7]	2 124<br>[1 689, 2 558]	1.66<br>[1.63, 1.69]	1 192<br>[1 055, 1 328]	1.09<br>[1.06, 1.12]	9 644<br>[6 096, 13 193]
PCG rheumatic conditions	1.64<br>[1.61, 1.66]	- 1<br>[- 332, 330]	1.64<br>[1.62, 1.67]	458<br>[352, 564]	0.85<br>[0.82, 0.87]	8 762<br>[5 847, 11 677]
PCG thyroid disorders	1.47<br>[1.43, 1.51]	- 306<br>[- 850, 237]	1.47<br>[1.43, 1.51]	454<br>[273, 635]	0.90<br>[0.87, 0.94]	2 587<br>[- 2 050, 7 223]
PCG other rare diseases	2.26<br>[2.17, 2.35]	4 675<br>[3 889, 5 462]	2.22<br>[2.14, 2.3]	3022<br>[2 766, 3 277]	1.54<br>[1.49, 1.6]	- 1 274<br>[- 5 112, 2 564]
Urbanisation: agglomeration	0.97<br>[0.96, 0.99]	- 115<br>[- 436, 206]	0.98<br>[0.97, 0.99]	- 154<br>[- 256, - 53]	0.98<br>[0.96, 1]	1 242<br>[- 1 460, 3 945]
Urbanisation: rural	0.91<br>[0.9, 0.92]	- 44<br>[- 461, 374]	0.91<br>[0.9, 0.92]	- 307<br>[- 437, - 177]	1.13<br>[1.09, 1.16]	- 8 008<br>[- 11 371, - 4 645]
Greater Region: Lake Geneva	1.2<br>[1.17, 1.23]	2 819<br>[2 116, 3 523]	1.19<br>[1.16, 1.22]	2 131<br>[1 899, 2 362]	1.01<br>[0.97, 1.06]	18 469<br>[13 431, 23 508]
	1.07	- 771	1.06	- 499	0.82	8 683

Greater Region: Espace Mittelland	[1.05, 1.09]	[- 1 247, - 295]	[1.04, 1.08]	[- 650, - 348]	[0.79, 0.85]	[4 836, 12 529]
Greater Region: Northwestern Switzerland	1.1<br>[1.09, 1.12]	656<br>[217, 1 096]	1.10<br>[1.09, 1.12]	- 134<br>[- 272, 3]	0.95<br>[0.92, 0.98]	11 761<br>[8 460, 15 063]
Greater Region: Eastern Switzerland	0.93<br>[0.91, 0.96]	- 504<br>[- 1 182, 173]	0.94<br>[0.91, 0.96]	- 900<br>[- 1 105, - 695]	0.97<br>[0.92, 1.02]	6 304<br>[747, 11 861]
Greater Region: Southern Switzerland	1.26<br>[1.24, 1.29]	-720<br>[- 1 247, - 193]	1.26<br>[1.24, 1.29]	241<br>[68, 414]	0.67<br>[0.64, 0.7]	20 870<br>[15 771, 25 969]
Greater Region: Central Switzerland	0.94<br>[0.91, 0.96]	- 676<br>[- 1 379, 26]	0.93<br>[0.91, 0.95]	- 237<br>[- 463, - 11]	0.91<br>[0.86, 0.96]	- 3 079<br>[- 8 627, 2 470]
Observations	798 940	545 385	798 940	543 131	798 940	47 028

Notes: The brackets show 95% confidence intervals. Abbreviations: AF: atrial fibrillation, GLM: generalized linear model, PCG: pharmaceutical cost groups.

Table S5. Regression results from OLS-based two part modelling.

	Total costs		Outpatient costs		Inpatient costs	
	Odds ratio (logistic part)	*Cost estimate (OLS part)*	*Odds ratio (logistic part)*	*Cost estimate (OLS part)*	*Odds ratio (logistic part)*	*Cost estimate (OLS part)*
Cohort: Swiss-AF	1.5 [1.46, 1.54]	5 744 [5 210, 6 277]	1.46 [1.42, 1.5]	1 043 [860, 1 226]	1.13 [1.08, 1.17]	37 322 [32 916, 41 728]
Month	1.00 [1.00, 1.00]	5.47 [- 2.49, 13.43]	1.00 [1.00, 1.00]	26.33 [23.61, 29.06]	1.00 [1.00, 1.00]	- 330.16 [- 397.79, -262.52]
Age	1.03 [1.03, 1.03]	208 [191, 225]	1.03 [1.02, 1.03]	12 [6, 17]	1.09 [1.09, 1.09]	- 2 833 [- 2 975, - 2 690]
Sex: Male	0.92 [0.91, 0.93]	3 378 [3 090, 3 666]	0.92 [0.91, 0.93]	1 802 [1 703, 1 901]	1.05 [1.02, 1.07]	9 021 [6 663, 11 379]
PCG acid related disorders	1.69 [1.66, 1.72]	2 568 [2 239, 2 896]	1.66 [1.63, 1.69]	1 454 [1 341, 1 566]	1.30 [1.27, 1.34]	- 1 799 [- 4 324, 726]
PCG bone diseases	1.94 [1.87, 2.01]	6 789 [6 167, 7 411]	1.91 [1.84, 1.98]	5 529 [5 316, 5 742]	0.97 [0.93, 1.02]	13 650 [9 006, 18 294]
PCG cancer	2.12 [2.03, 2.21]	17 579 [16 855, 18 302]	2.09 [2, 2.18]	14 032 [13 784, 14 279]	1.22 [1.16, 1.29]	15 126 [9 514, 20 738]
PCG cardio	1.89 [1.87, 1.91]	- 325 [- 633, - 16]	1.90 [1.87, 1.92]	339 [234, 445]	0.98 [0.96, 1.01]	7 699 [5 040, 10 358]
PCG dementia	2.05 [1.98, 2.13]	1 897 [1 343, 2 451]	1.93 [1.87, 2]	869 [679, 1 060]	2.14 [2.07, 2.22]	- 23 773 [- 26 889, - 20 657]
PCG diabetes	1.67 [1.64, 1.7]	3 847 [3 497, 4 198]	1.66 [1.63, 1.69]	2 435 [2 315, 2 555]	1.25 [1.21, 1.28]	5 543 [2 641, 8 446]
PCG epilepsy	2.26 [2.18, 2.34]	6 908 [6 395, 7 421]	2.19 [2.12, 2.27]	3 450 [3 274, 3 626]	1.58 [1.53, 1.63]	- 8 073 [- 11 290, - 4 856]
PCG glaucoma	1.56	- 638	1.56	541	0.87	- 832

	[1.53, 1.59]	[- 1 028, - 249]	[1.53, 1.59]	[407, 674]	[0.85, 0.9]	[- 4 077, 2 413]
PCG gout	1.35<br>[1.32, 1.39]	1 766<br>[1 258, 2 275]	1.36<br>[1.32, 1.39]	806<br>[632, 980]	1.02<br>[0.98, 1.06]	8 036<br>[3 787, 12 285]
PCG hyperlipidemia	1.32<br>[1.3, 1.33]	- 1 269<br>[- 1 545, - 992]	1.32<br>[1.3, 1.33]	- 493<br>[- 588, - 398]	0.86<br>[0.84, 0.88]	4 874<br>[2 483, 7 266]
PCG iron deficiency	1.57<br>[1.51, 1.64]	6 803<br>[6 164, 7 442]	1.56<br>[1.5, 1.62]	4 636<br>[4 417, 4 855]	1.20<br>[1.15, 1.25]	1 784<br>[- 2 597, 6 165]
PCG pain	1.47<br>[1.44, 1.5]	6 773<br>[6 412, 7 134]	1.45<br>[1.42, 1.49]	2 817<br>[2 693, 2 941]	1.82<br>[1.78, 1.87]	- 2 141<br>[- 4 690, 407]
PCG psychiatric	2.19<br>[2.15, 2.24]	2 214<br>[1 883, 2 544]	2.17<br>[2.12, 2.21]	1 306<br>[1 193, 1 419]	1.60<br>[1.56, 1.64]	- 15 352<br>[- 17 778, - 12 926]
PCG antipsychotic	2.92<br>[2.78, 3.06]	9 387<br>[8 764, 10 009]	2.50<br>[2.39, 2.62]	1 256<br>[1 042, 1 470]	5.98<br>[5.8, 6.17]	- 34 176<br>[- 37 082, - 31 269]
PCG respiratory	1.67<br>[1.64, 1.7]	2 062<br>[1 692, 2 433]	1.66<br>[1.63, 1.69]	1 180<br>[1 053, 1 307]	1.09<br>[1.06, 1.12]	5 583<br>[2 507, 8 660]
PCG rheumatic conditions	1.64<br>[1.61, 1.66]	- 648<br>[- 956, - 341]	1.64<br>[1.62, 1.67]	26<br>[- 79, 131]	0.85<br>[0.82, 0.87]	7 306<br>[4 712, 9 900]
PCG thyroid disorders	1.47<br>[1.43, 1.51]	72<br>[- 442, 585]	1.47<br>[1.43, 1.51]	495<br>[319, 671]	0.90<br>[0.87, 0.94]	2 701<br>[- 1 520, 6 922]
PCG other rare diseases	2.26<br>[2.17, 2.35]	4 566<br>[3 979, 5 152]	2.22<br>[2.14, 2.3]	2 874<br>[2 673, 3 075]	1.54<br>[1.49, 1.6]	- 5 617<br>[- 9 245, - 1 989]
Urbanisation: agglomeration	0.97<br>[0.96, 0.99]	- 213<br>[- 511, 85]	0.98<br>[0.97, 0.99]	- 279<br>[- 381, - 177]	0.98<br>[0.96, 1]	1 560<br>[- 927, 4 046]
Urbanisation: rural	0.91<br>[0.9, 0.92]	- 416<br>[- 803, - 29]	0.91<br>[0.9, 0.92]	- 501<br>[- 634, - 369]	1.13<br>[1.09, 1.16]	- 6 086<br>[- 9 376, - 2 796]
Greater Region: Lake Geneva	1.2<br>[1.17, 1.23]	3 425<br>[2 832, 4 018]	1.19<br>[1.16, 1.22]	2 319<br>[2 116, 2 522]	1.01<br>[0.97, 1.06]	15 877<br>[11 270, 20 483]
	1.07	- 899	1.06	- 583	0.82	7 964

Greater Region: Espace Mittelland	[1.05, 1.09]	[- 1 353, - 445]	[1.04, 1.08]	[- 739, - 428]	[0.79, 0.85]	[4 098, 11 830]
Greater Region: Northwestern Switzerland	1.1<br>[1.09, 1.12]	493<br>[85, 901]	1.10<br>[1.09, 1.12]	- 255<br>[- 395, - 115]	0.95<br>[0.92, 0.98]	12 598<br>[9 256, 15 939]
Greater Region: Eastern Switzerland	0.93<br>[0.91, 0.96]	- 330<br>[- 979, 319]	0.94<br>[0.91, 0.96]	- 857<br>[- 1 079, - 634]	0.97<br>[0.92, 1.02]	1 036<br>[- 4 494, 6 567]
Greater Region: Southern Switzerland	1.26<br>[1.24, 1.29]	- 803<br>[- 1 308, - 298]	1.26<br>[1.24, 1.29]	171<br>[- 2, 344]	0.67<br>[0.64, 0.7]	13 761<br>[9 162, 18 360]
Greater Region: Central Switzerland	0.94<br>[0.91, 0.96]	- 781<br>[- 1 460, - 101]	0.93<br>[0.91, 0.95]	- 167<br>[- 399, 66]	0.91<br>[0.86, 0.96]	- 1 150<br>[- 7 175, 4 875]
Observations	798 940	545 385	798 940	543 131	798 940	47 028

Notes: The brackets show 95% confidence intervals. Abbreviations: AF: atrial fibrillation, OLS: ordinary least square, PCG: pharmaceutical cost groups.

Table S6. Comparison of cohort characteristics before and after propensity score matching.

	Before propensity score matching (1:1)				*After propensity score matching (1:1)*			
	**Swiss-AF**	**Controls**			**Swiss-AF**	**Controls**		
N	1 024	16 556	p	SMD	958	958	p	SMD
**Characteristics**								
Age *mean (SD)*	73.04 (8.17)	72.64 (8.52)	0.139	0.049	73.01 (8.20)	72.96 (8.37)	0.908	0.005
Sex: Male *N (%)*	741 (72.4)	11766 (71.1)	0.394	0.029	694 (72.4)	652 (68.1)	0.04	0.096
**Comorbidities (PCG)** *N (%)*								
Acid related	397 (38.8)	2802 (17.4)	<0.001	0.491	372 (38.8)	387 (40.4)	0.513	0.032
Bone	44 (4.3)	644 (4.0)	0.719	0.014	43 (4.5)	42 (4.4)	1	0.005
Cancer	35 (3.4)	510 (3.2)	0.748	0.013	33 (3.4)	29 (3.0)	0.699	0.024
Cardiovascular	754 (73.8)	10381 (63.7)	<0.001	0.22	706 (73.7)	676 (70.6)	0.14	0.07
Dementia	27 (2.6)	797 (5.0)	0.001	0.122	27 (2.8)	28 (2.9)	1	0.006
Diabetes	122 (11.9)	2298 (14.3)	0.04	0.07	110 (11.5)	101 (10.5)	0.559	0.03
Epilepsy	66 (6.5)	982 (6.1)	0.719	0.014	64 (6.7)	67 (7.0)	0.856	0.012
Glaucoma	103 (10.1)	1634 (10.2)	0.939	0.004	98 (10.2)	115 (12.0)	0.245	0.056
Gout	96 (9.4)	935 (5.8)	<0.001	0.134	89 (9.3)	87 (9.1)	0.937	0.007
Hyperlipidemia	425 (41.6)	5649 (35.0)	<0.001	0.136	395 (41.2)	371 (38.7)	0.283	0.051
Iron deficiency	66 (6.5)	567 (3.5)	<0.001	0.134	60 (6.3)	62 (6.5)	0.925	0.009
Pain	386 (37.8)	2484 (15.4)	<0.001	0.523	363 (37.9)	358 (37.4)	0.85	0.011
Psychiatric	266 (26.0)	2837 (17.6)	<0.001	0.204	250 (26.1)	269 (28.1)	0.355	0.045
Antipsychotic	16 (1.6)	878 (5.5)	<0.001	0.213	16 (1.7)	15 (1.6)	1	0.008
Respiratory	144 (14.1)	1915 (11.9)	0.045	0.064	137 (14.3)	148 (15.4)	0.521	0.032
Rheumatic	406 (39.7)	3074 (19.1)	<0.001	0.465	378 (39.5)	378 (39.5)	1	<0.001
Thyroid	87 (8.5)	908 (5.7)	<0.001	0.111	78 (8.1)	88 (9.2)	0.465	0.037
Other rare diseases	27 (2.6)	696 (4.4)	0.011	0.093	27 (2.8)	20 (2.1)	0.376	0.047
**Socioeconomic**								
Mother tongue *N (%)*			0.001	0.116			0.253	0.076
German	755 (73.7)	12944 (78.2)			737 (76.9)	759 (79.2)		
French	141 (13.8)	1708 (10.3)			132 (13.8)	108 (11.3)		

Italian	128 (12.5)	1904 (11.5)			89 (9.3)	91 (9.5)		
Urbanisation *N (%)*			0.236	0.056			0.973	0.011
Urban	253 (26.2)	4330 (26.2)			252 (26.3)	250 (26.1)		
Agglomeration	500 (51.9)	8953 (54.1)			497 (51.9)	502 (52.4)		
Rural	211 (21.9)	3273 (19.8)			209 (21.8)	206 (21.5)		
**Greater Region *N (%)***			<0.001	0.167			0.994	0.038
Zurich	125 (12.2)	2083 (12.6)			120 (12.5)	128 (13.4)		
Lake Geneva Region	56 (5.5)	1086 (6.6)			53 (5.5)	53 (5.5)		
Espace Mitelland	289 (28.2)	3702 (22.4)			278 (29.0)	266 (27.8)		
Northwestern Switzerland	310 (30.3)	5990 (36.2)			307 (32.0)	308 (32.2)		
Eastern Switzerland	67 (6.5)	944 (5.7)			66 (6.9)	66 (6.9)		
Southern Switzerland	125 (12.2)	1904 (11.5)			86 (9.0)	91 (9.5)		
Central Switzerland	52 (5.1)	847 (5.1)			48 (5.0)	46 (4.8)		

Notes: Abbreviations: AF: atrial fibrillation, PCG: pharmaceutical cost groups, SD: standard deviation, SMD: standardized mean difference.

Table S7. Regression results from ordinary (single-part) OLS modelling.

	Total costs	Outpatient costs	Inpatient costs
Cohort: Swiss-AF	5 124<br>[4 726, 5 522]	1 125<br>[986, 1 263]	3 999<br>[3 636, 4 362]
Month	8<br>[3, 14]	20<br>[18, 22]	- 12<br>[- 17, - 7]
Age	201<br>[190, 212]	44<br>[40, 47]	158<br>[147, 168]
Sex: Male	2 197<br>[1 996, 2 398]	1 158<br>[1 088, 1 228]	1 039<br>[856, 1 223]
PCG acid related disorders	3 206<br>[2 953, 3 458]	1 780<br>[1 692, 1 868]	1 426<br>[1 195, 1 656]
PCG bone diseases	6 983<br>[6 502, 7 465]	5 330<br>[5 162, 5 497]	1 653<br>[1 214, 2 093]
PCG cancer	16 504<br>[15 944, 1 7063]	12 765<br>[12 570, 12 960]	3 738<br>[3 228, 4 249]
PCG cardio	1 379<br>[1 171, 1 587]	1 118<br>[1 045, 1 190]	261<br>[71, 451]
PCG dementia	2 907<br>[2 472, 3 342]	1 320<br>[1 168, 1 471]	1 587<br>[1 190, 1 984]
PCG diabetes	4 195<br>[3 930, 4 460]	2 599<br>[2 507, 2 691]	1 596<br>[1 354, 1 838]
PCG epilepsy	7 533<br>[7 127, 7 938]	3 836<br>[3 694, 3 977]	3 697<br>[3 327, 4 067]
PCG glaucoma	434<br>[143, 725]	932<br>[830, 1 033]	- 497<br>[- 763, - 232]
PCG gout	2 168	1 061	1 107

	[1 784, 2 553]	[927, 1 195]	[756, 1 458]
PCG hyperlipidemia	- 396<br>[- 598, - 194]	- 40<br>[- 110, 30]	- 356<br>[- 540, - 171]
PCG iron deficiency	6 671<br>[6 173, 7 170]	4 392<br>[4 219, 4 566]	2 279<br>[1 824, 2 734]
PCG pain	6 620<br>[6 341, 6 899]	2 821<br>[2 724, 2 919]	3 799<br>[3 544, 4 054]
PCG psychiatric	3 328<br>[3 072, 3 584]	1 907<br>[1 818, 1 996]	1 421<br>[1 188, 1 654]
PCG antipsychotic	10 213<br>[9 717, 10 709]	1 960<br>[1 787, 2 132]	8 254<br>[7 800, 8 707]
PCG respiratory	2 669<br>[2 390, 2 949]	1 520<br>[1 423, 1 617]	1 149<br>[894, 1 404]
PCG rheumatic conditions	328<br>[96, 561]	528<br>[447, 609]	- 200<br>[- 412, 12]
PCG thyroid disorders	656<br>[268, 1 044]	724<br>[589, 859]	- 68<br>[- 422, 286]
PCG other rare diseases	5 353<br>[4 894, 5 812]	3 268<br>[3 108, 3 428]	2 084<br>[1 665, 2 503]
Urbanisation: agglomeration	- 205<br>[- 412, 2]	- 217<br>[- 289, - 145]	12<br>[- 177, 202]
Urbanisation: rural	-503<br>[-768, -237]	-455<br>[-548, -363]	-48<br>[-290, 195]
Greater Region: Lake Geneva	3 002<br>[2 585, 3 419]	1 963<br>[1 818, 2 108]	1 038<br>[658, 1 419]
Greater Region: Espace Mittelland	- 406<br>[- 716, - 97]	- 267<br>[- 375, - 160]	- 139<br>[- 421, 144]
	592	- 29	620

Greater Region: Northwestern Switzerland	[313, 870]	[- 126, 68]	[366, 875]
Greater Region: Eastern Switzerland	- 394<br>[- 826, 39]	- 623<br>[- 773, - 472]	229<br>[-166, 624]
Greater Region: Southern Switzerland	9<br>[- 342, 360]	475<br>[353, 598]	- 466<br>[- 787, - 146]
Greater Region: Central Switzerland	- 610<br>[- 1 059, - 162]	- 197<br>[- 354, - 41]	- 413<br>[- 823, - 3]
Observations	798 940	798 940	798 940

Notes: The brackets show 95% confidence intervals. Abbreviations: AF: atrial fibrillation, OLS: ordinary least square, PCG: pharmaceutical cost groups.

Figure S3. Cost of illness estimated total AF costs per year, in Switzerland and per inhabitant in the general population.

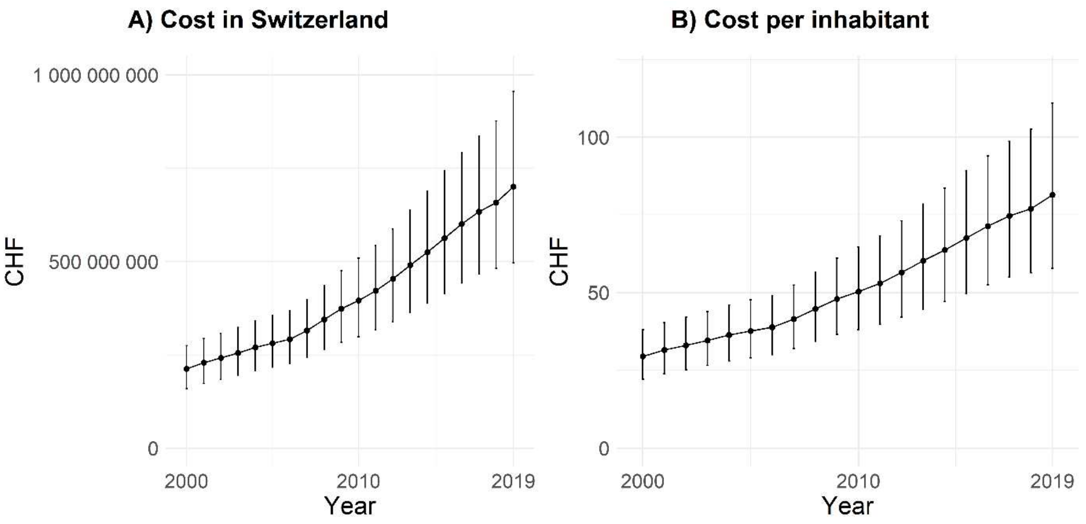

Figure S4. Cost of illness by sex and age group: estimated total AF costs per year, in Switzerland and per inhabitant in the general population.

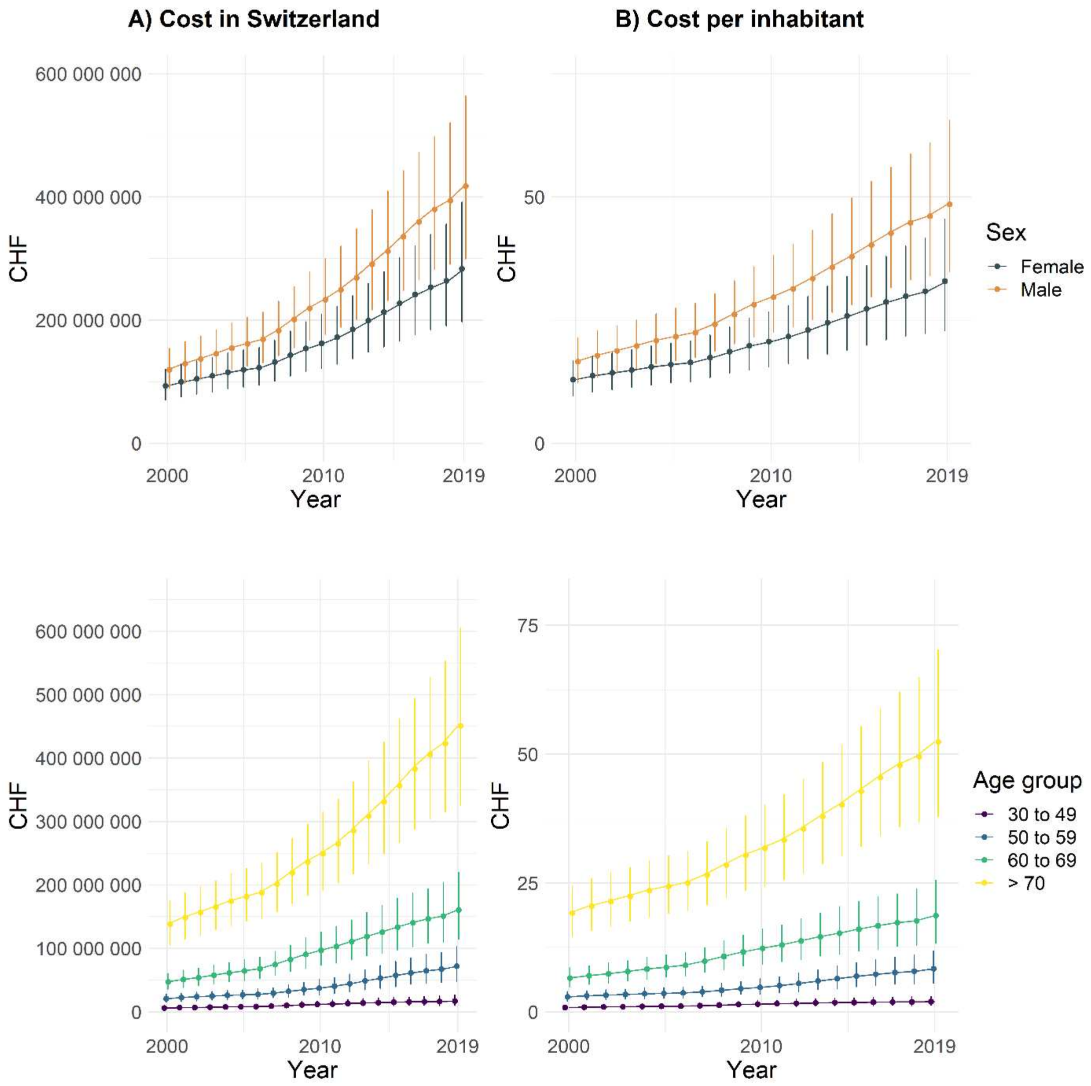

Swiss-AF investigators

University Hospital Basel and Basel University: Stefanie Aeschbacher, Katalin Bhend, Steffen Blum, Leo Bonati, David Conen, Ceylan Eken, Urs Fischer, Corinne Girroy, Elisa Hennings, Elena Herber, Vasco Iten, Philipp Krisai, Michael Kühne, Maurin Lampart, Mirko Lischer, Nina Mäder, Christine Meyer-Zürn, Pascal Meyre, Andreas U. Monsch, Luke Mosher, Christian Müller, Stefan Osswald, Rebecca E. Paladini, Anne Springer, Christian Sticherling, Thomas Szucs, Gian Völlmin.
Principal Investigator: Stefan Osswald; Local Principal Investigator: Michael Kühne

University Hospital Bern: Faculty: Faculty: Drahomir Aujesky, Juerg Fuhrer, Laurent Roten, Simon Jung, Heinrich Mattle; Research fellows: Seraina Netzer, Luise Adam, Carole Elodie Aubert, Martin Feller, Axel Loewe, Elisavet Moutzouri, Claudio Schneider; Study nurses: Tanja Flückiger, Cindy Groen, Lukas Ehrsam, Sven Hellrigl, Alexandra Nuoffer, Damiana Rakovic, Nathalie Schwab, Rylana Wenger, Tu Hanh Zarrabi Saffari. Local Principal Investigator: Nicolas Rodondi, Tobias Reichlin

Stadtspital Triemli Zurich: Christopher Beynon, Roger Dillier, Michèle Deubelbeiss, Franz Eberli, Christine Franzini, Isabel Juchli, Claudia Liedtke, Samira Murugiah, Jacqueline Nadler, Thayze Obst, Jasmin Roth, Fiona Schlomowitsch, Xiaoye Schneider, Katrin Studerus, Noreen Tynan, Dominik Weishaupt. Local Principal Investigator: Andreas Müller

Kantonspital Baden: Simone Fontana, Corinne Friedli, Silke Kuest, Karin Scheuch, Denise Hischier, Nicole Bonetti, Alexandra Grau, Jonas Villinger, Eva Laube, Philipp Baumgartner, Mark Filipovic, Marcel Frick, Giulia Montrasio, Stefanie Leuenberger, Franziska Rutz. Local Principal Investigator: Jürg-Hans Beer

Cardiocentro Lugano: Angelo Auricchio, Adriana Anesini, Cristina Camporini, Maria Luce Caputo, Francois Regoli, Martina Ronchi. Local Principal Investigator: Giulio Conte

Kantonsspital St. Gallen: Roman Brenner, David Altmann, Michaela Gemperle. Local Principal Investigator: Peter Ammann

Hôpital Cantonal Fribourg: Mathieu Firmann, Sandrine Foucras, Martine Rime. Local Principal Investigator: Daniel Hayoz

Luzerner Kantonsspital: Benjamin Berte, Kathrin Bühler, Virgina Justi, Frauke Kellner-Weldon, Melanie Koch, Brigitta Mehmann, Sonja Meier, Myriam Roth, Andrea Ruckli-Kaeppeli, Ian Russi, Kai Schmidt, Mabelle Young, Melanie Zbinden. Local Principal Investigator: Richard Kobza

Ente Ospedaliero Cantonale Lugano: Elia Rigamonti, Carlo Cereda, Alessandro Cianfoni, Maria Luisa De Perna, Jane Frangi-Kultalahti, Patrizia Assunta Mayer Melchiorre, Anica Pin,Tatiana Terrot, Luisa Vicari. Local Principal Investigator: Giorgio Moschovitis.

University Hospital Geneva: Georg Ehret, Hervé Gallet, Elise Guillermet, Francois Lazeyras, Karl-Olof Lovblad, Patrick Perret, Philippe Tavel, Cheryl Teres. Local Principal Investigator: Dipen Shah

University Hospital Lausanne: Nathalie Lauriers, Marie Méan, Sandrine Salzmann, Jürg Schläpfer. Local Principal Investigator: Alessandra Pia Porretta

Bürgerspital Solothurn: Andrea Grêt, Jan Novak, Sandra Vitelli. Local Principal Investigator: Frank-Peter Stephan

Ente Ospedaliero Cantonale Bellinzona: Jane Frangi-Kultalahti, Augusto Gallino, Luisa Vicari. Local Principal Investigator: Marcello Di Valentino

University of Zurich/University Hospital Zurich: Helena Aebersold, Fabienne Foster, Matthias Schwenkglenks.

Medical Image Analysis Center AG Basel: Jens Würfel (Head), Anna Altermatt, Michael Amann, Marco Düring, Petra Huber, Esther Ruberte, Tim Sinnecker, Vanessa Zuber.

Clinical Trial Unit Basel: Michael Coslovsky (Head), Pascal Benkert, Gilles Dutilh, Milica Markovic, Pia Neuschwander, Patrick Simon, Olivia Wunderlin

Schiller AG Baar: Ramun Schmid
