# Supplementary material for: Estimating the cost impact of atrial fibrillation using a prospective cohort study and population-based controls
Source: BMJ Open. 2023 Sep 14;13(9):e072080. doi: 10.1136/bmjopen-2023-072080 (PMC10503354; doi:10.1136/bmjopen-2023-072080)
Supplement: Author's manuscript [file bmjopen-2023-072080.draft_revisions.pdf]

BMJ Open is committed to open peer review. As part of this commitment we make the peer review history of every article we publish publicly available.

When an article is published we post the peer reviewers' comments and the authors' responses online. We also post the versions of the paper that were used during peer review. These are the versions that the peer review comments apply to.

The versions of the paper that follow are the versions that were submitted during the peer review process. They are not the versions of record or the final published versions. They should not be cited or distributed as the published version of this manuscript.

BMJ Open is an open access journal and the full, final, typeset and author-corrected version of record of the manuscript is available on our site with no access controls, subscription charges or pay-per-view fees (http://bmjopen.bmj.com).

If you have any questions on BMJ Open's open peer review process please email info.bmjopen@bmj.com

BMJ Open

Estimating the cost impact of atrial fibrillation using a prospective cohort study and population-based controls

Journal:	*BMJ Open*
Manuscript ID	bmjopen-2023-072080
Article Type:	Original research
Date Submitted by the Author:	26-Jan-2023

Complete List of Authors:	Aebersold, Helena; University of Zurich, Epidemiology, Biostatistics and Prevention Institute<br>Witassek, Fabienne ; University of Zurich, Epidemiology, Biostatistics and Prevention Institute<br>Serra-Burriel, Miquel; University of Zurich, Epidemiology, Biostatistics and Prevention Institute<br>Brüngger, Beat; Helsana Versicherungen AG, Department of Health Sciences<br>Aeschbacher, Stefanie; University Hospital Basel, Department of Medicine, Cardiology Division; University Hospital Basel, Cardiovascular Research Institute Basel<br>Beer, Jürg-Hans; Baden Cantonal Hospital, Department of Medicine, Cantonal Hospital of Baden; University of Zurich, Center for Molecular Cardiology<br>Blozik, Eva; University of Zurich, Institute of Primary Care<br>blum, manuel; University of Bern Institute of Primary Health Care; Inselspital University Hospital Bern, Department of General Internal Medicine<br>Bonati, Leo; University of Basel, Department of Neurology; Reha Rheinfelden, Research Department<br>Conen, David; McMaster University, Population Health Research Institute<br>Conte, Giulio; Cardiocentro Ticino, Division of Cardiology<br>Felder, Stefan; University of Basel, Faculty of Business and Economics<br>Huber, Carola; Helsana Versicherungen AG, Department of Health Sciences<br>Kuehne, Michael; University of Basel, Department of Medicine, Cardiology Division; University Hospital Basel, Cardiovascular Research Institute Basel<br>Moschovitis, Giorgio; Ospedale Regionale di Lugano-Civico e Italiano, Division of Cardiology<br>Mueller, Andreas; Municipal Hospital Triemli, Department of Cardiology<br>Paladini, Rebecca E; University Hospital Basel, Department of Medicine, Cardiology Division; University Hospital Basel, Cardiovascular Research Institute Basel<br>Reichlin, Tobias; Inselspital University Hospital Bern, Cardiology<br>Rodondi, Nicolas; University of Bern Institute of Primary Health Care; Inselspital University Hospital Bern, Department of General Internal Medicine<br>Springer, Anne; University Hospital Basel, Department of Medicine, Cardiology Division; University Hospital Basel, Cardiovascular Research Institute Basel

	Stauber, Annina; Municipal Hospital Triemli, Department of Cardiology<br>Sticherling, Christian; University Hospital Basel, Department of Medicine, Cardiology Division; University Hospital Basel, Cardiovascular Research Institute Basel<br>Szucs, Thomas; University of Basel, Institute of Pharmaceutical Medicine<br>Osswald , Stefan; University Hospital Basel, Department of Medicine, Cardiology Division; University Hospital Basel, Cardiovascular Research Institute Basel<br>Schwenkglenks, M; University of Zurich, Epidemiology, Biostatistics and Prevention Institute; University of Basel, Institute of Pharmaceutical Medicine
Keywords:	HEALTH ECONOMICS, CARDIOLOGY, Cardiac Epidemiology < CARDIOLOGY

SCHOLARONE™
Manuscripts

I, the Submitting Author has the right to grant and does grant on behalf of all authors of the Work (as defined in the below author licence), an exclusive licence and/or a non-exclusive licence for contributions from authors who are: i) UK Crown employees; ii) where BMJ has agreed a CC-BY licence shall apply, and/or iii) in accordance with the terms applicable for US Federal Government officers or employees acting as part of their official duties; on a worldwide, perpetual, irrevocable, royalty-free basis to BMJ Publishing Group Ltd ("BMJ") its licensees and where the relevant Journal is co-owned by BMJ to the co-owners of the Journal, to publish the Work in this journal and any other BMJ products and to exploit all rights, as set out in our licence.

The Submitting Author accepts and understands that any supply made under these terms is made by BMJ to the Submitting Author unless you are acting as an employee on behalf of your employer or a postgraduate student of an affiliated institution which is paying any applicable article publishing charge ("APC") for Open Access articles. Where the Submitting Author wishes to make the Work available on an Open Access basis (and intends to pay the relevant APC), the terms of reuse of such Open Access shall be governed by a Creative Commons licence – details of these licences and which Creative Commons licence will apply to this Work are set out in our licence referred to above.

Other than as permitted in any relevant BMJ Author's Self Archiving Policies, I confirm this Work has not been accepted for publication elsewhere, is not being considered for publication elsewhere and does not duplicate material already published. I confirm all authors consent to publication of this Work and authorise the granting of this licence.

For peer review only

Estimating the cost impact of atrial fibrillation using a prospective cohort study and population-based controls

Authors

Aebersold Helena[a], Foster-Witassek Fabienne[a], Serra-Burriel Miquel[a], Beat Brüngger[b], Aeschbacher Stefanie[c,d], Beer Jürg H.[e,f], Blozik Eva[g], Blum Manuel R.[h,q], Bonati Leo H.[i,j], Conen David[k], Conte Giulio[l], Felder Stefan[m], Huber Carola A.[b], Kühne Michael[c,d], Moschovitis Giorgio[n], Müller Andreas[o], Paladini Rebecca E.[c,d], Reichlin Tobias[p], Rodondi Nicolas[h,q], Springer Anne[c,d], Stauber Annina[o], Sticherling Christian[c,d], Szucs Thomas[r], Osswald Stefan[c,d], Schwenkglenks Matthias[a,r], on behalf of the Swiss-AF Investigators

[a] Epidemiology, Biostatistics and Prevention Institute, University of Zurich, Zurich, Switzerland

[b] Department of Health Sciences, Helsana Group, Zurich, Switzerland

[c] Cardiology Division, Department of Medicine, University Hospital Basel, Switzerland

[d] Cardiovascular Research Institute Basel, University Hospital Basel, Switzerland

[e] Department of Medicine, Cantonal Hospital of Baden, Switzerland

[f] Center for Molecular Cardiology, University of Zürich, Switzerland

[g] Institute of Primary Care, University of Zurich, Zurich, Switzerland

[h] Institute of Primary Health Care (BIHAM), University of Bern, Switzerland

[i] Department of Neurology, University Hospital Basel, Basel Switzerland

[j] Research Department, Reha Rheinfelden, Rheinfelden, Switzerland

[k] Population Health Research Institute, McMaster University, Hamilton, Canada

[l] Division of Cardiology, Cardiocentro Ticino (CCT), Lugano, Switzerland

[m] University of Basel, Faculty of Business and Economics, Switzerland

[n] Division of Cardiology, Ente Ospedaliero Cantonale (EOC), Istituto Cardiocentro Ticino, Ospedale Regionale di Lugano, Lugano, Switzerland

[o] Department of Cardiology, Triemli Hospital Zurich, Switzerland

[p] Department of Cardiology, Inselspital, Bern University Hospital, University of Bern, Switzerland

[q] Department of General Internal Medicine, Inselspital, Bern University Hospital, University of Bern, Switzerland

[r] Institute of Pharmaceutical Medicine (ECPM), University of Basel, Basel, Switzerland

Correspondence

Helena Aebersold

University of Zurich

For peer review only - http://bmjopen.bmj.com/site/about/guidelines.xhtml

Epidemiology, Biostatistics & Prevention Institute (EBPI)
Hirschengraben 84, 8001 Zurich, Switzerland
helena.aebersold@uzh.ch

Word Count: 3,020

For peer review only

For peer review only - http://bmjopen.bmj.com/site/about/guidelines.xhtml

Abstract

Aims

Atrial Fibrillation (AF) costs are expected to be substantial, but cost comparisons with the general population are scarce. Using data from the prospective Swiss-AF cohort study and population-based controls, we estimated the impact of AF on direct healthcare costs from the Swiss statutory health insurance perspective.

Methods

Swiss-AF patients, enrolled from 2014-2017, had documented, prevalent AF. Yearly follow-ups collected clinical data, and health insurance claims in 42% of the patients. Controls from a health insurance claims database were matched for demographics and region. The cost impact of AF was estimated using five different methods: i) ordinary least square regression (OLS), ii) OLS-based two-part modelling, iii) generalised linear model (GLM)-based two-part modelling, iv) 1:1 nearest neighbour propensity score matching, and v) a cost adjudication algorithm using Swiss-AF data non-comparatively and considering clinical data. Cost-of-illness at the Swiss national level was modelled using obtained cost estimates, prevalence from the Global Burden of Disease Project, and Swiss population data.

Results

The 1,024 Swiss-AF patients with available claims data were compared with 16,556 controls without known AF. Average yearly AF-related direct healthcare costs amounted to CHF 5,600 (EUR 5,091), while non-AF related healthcare costs were CHF 11,100 (EUR 10,091). All five methods yielded comparable results. AF-related costs at the national level were estimated to amount to 1% of Swiss healthcare expenditure.

Conclusions

We robustly found direct medical costs of AF patients are 50% higher than those of population-based controls. Such information on the incremental cost burden of AF may support healthcare capacity planning.

Keywords

atrial fibrillation, cost-of-illness, two-part model, population-based controls, healthcare costs

For peer review only - http://bmjopen.bmj.com/site/about/guidelines.xhtml

Strengths and limitations

- This study used 5 years of follow-up data from a large prospective cohort of prevalent atrial fibrillation (AF) patients.
- The direct medical cost impact of AF was assessed by comparison with population-based controls drawn from a large health insurance database.
- Several regression-based and propensity score-based methods were used to judge robustness and AF costs were also assessed using a non-comparative approach.
- The cohort of AF patients may not be fully representative of all AF patients.
- A limited degree of residual presence of AF in the control population cannot be ruled out.

For peer review only

For peer review only - http://bmjopen.bmj.com/site/about/guidelines.xhtml

Introduction

Atrial fibrillation (AF) is the most common form of serious arrhythmia worldwide, and a major cause of stroke and heart failure. More than 11 million people live with AF in Europe.[1,2] Given demographic ageing, Europe is expected to face a larger increase in AF prevalence by 2050 than any other region globally.[1]

Several studies on cost-of-illness of AF have estimated costs at the patient or nationwide levels. Direct healthcare costs per patient were estimated to range from EUR 2,315–3,307 annually in Europe[3–6], and from USD 6,410–8,705 in the USA[7,8]. At the national level, direct costs of AF in Europe may range from EUR 660–2,548 million[9–12], in the US they were estimated at around USD 6 billion[8,13]. These costs are substantial, accounting for 0.28-1.7% of the national health expenditures of these countries[12,14–16].

So far, most attempts assessing the cost impact of AF remained descriptive. To our knowledge, only two studies[8,16] compared costs between AF patients and a control population. Even less evidence is available for cost changes since 2010, as most cost-of-illness studies rely on data collected earlier.

We used a recent real-world dataset from a large prospective cohort study of AF patients to assess the yearly cost impact of AF. Comparing with a population-based control sample, direct healthcare costs of AF were estimated at the patient level and transferred to the national level. Results were compared with estimates resulting from an adjudication algorithm only using the cohort data in a non-comparative approach.

For peer review only

For peer review only - http://bmjopen.bmj.com/site/about/guidelines.xhtml

Methods

Study Design and Data Sources

Swiss-AF is a large, ongoing prospective observational cohort study across 14 clinical centres in Switzerland, investigating AF-related cognition, complications, and economic aspects. Patients were enrolled between 2014 and 2017 if they had a history of documented AF and were older than 65 years; 228 patients were enrolled aged 45-64 to enhance the study of socio-economic aspects. A data cut of 2014-2020 was used in this analysis. The detailed study setup has been published earlier.[17]

Alongside clinical data, health economic data were collected. These included medical resource use at the study centres, and health insurance claims from four cooperating health insurers covering 42% of the study sample. In Switzerland, health insurance is compulsory and offered to anyone, covering inpatient and outpatient services. The benefit package is uniform across the country and defined by law.

To assess the cost impact of AF, a population-based reference sample was provided by Helsana, an insurer covering about 15% of the Swiss population. Helsana enrolees were eligible for the reference sample if they were not Swiss-AF patients, were in the same age range as the Swiss-AF population, and had statutory health insurance claims data available for a period equivalent to the one available for Swiss-AF patients. For the reference sample a subset of 19,002 patients was randomly selected, frequency-matched to the Swiss-AF patients by age, gender and geographic region (supplementary **Table S1**). To ensure similar observation times, start dates for the controls were randomly assigned using the distribution of Swiss-AF enrolment dates. Sensitivity analyses with different starting and ending dates were run without altering the results significantly. Individuals within the reference sample could have AF, as Swiss claims data do not have direct diagnosis information for outpatient services. Hence, a categorization algorithm (supplementary **Table S2**) was developed together with clinicians from the Swiss-AF centres to distinguish such persons. Using codes from the International Classification of Diseases 10th Revision (ICD10)[18], the Swiss diagnosis related group-based flat fee reimbursement system for inpatient episodes (SwissDRG)[19], the Swiss invasive medical procedures catalogue (CHOP)[20], the anatomical therapeutic chemical classification (ATC) of medicines[21], and the national tariff for outpatient physician services (Tarmed)[22], three categories resulted: “AF likely”, “AF possible”, and “AF not obvious”. We assumed the category of “AF likely” to mainly contain severe AF patients, as most codes were hospitalisation based. Persons categorized as “AF possible” had codes possibly but not clearly allocable to AF. All other patients were classified as “AF not obvious” and considered as controls (**Figure 1**).

For peer review only - http://bmjopen.bmj.com/site/about/guidelines.xhtml

Equivalent claims data were available for the Swiss-AF and control patients, reflecting all claims for reimbursement by the Swiss statutory health insurance. The claims data included detailed information on outpatient services and drugs, and less detailed information on inpatient services based on SwissDRG[19]. Given the absence of clinical data for the control sample, the presence of major chronic morbidities was approximated, uniformly for Swiss-AF patients and controls, based on outpatient drug claims, using the pharmaceutical cost groups (PCG) approach[23].

Outcome Measures

Our main outcome of interest was the AF-induced part of direct medical healthcare costs from the perspective of the Swiss statutory health insurance. To assess the cost impact of AF, the Swiss-AF patients were compared with the population-based controls, using different multivariable regression methods: i) ordinary least square regression (OLS), ii) OLS-based two-part modelling, iii) generalised linear model (GLM)-based two-part modelling, and iv) 1:1 nearest neighbour propensity score matching. Furthermore, v) estimates were compared with AF costs estimated using a previously developed adjudication algorithm[24]. In brief, the AF-adjudication algorithm combined clinical event data collected in Swiss-AF with health insurance claims, adjudicating each cost component as AF-related or non-AF related. We distinguished between total, outpatient, and inpatient costs. All cost calculations considered individual start dates and follow-up times and were aggregated to a yearly level. Given the relative stability of prices over the observation period, costs were taken as recorded in the health insurance database. To facilitate comparison with other countries, main cost results are presented in Euros (EUR) in addition to Swiss francs (CHF), based on an exchange rate (averaged 2014-2020) of EUR 1.0 = CHF 1.1. Individual follow-up times were censored at five years after the start date due to the small number of longer follow-up periods available.

Covariates

Covariates available for both the Swiss-AF and control population included the following types: Firstly, patient characteristics: age, sex, and area of residence (greater regions of Switzerland). Secondly, PCGs as proxies for comorbidities: acid related disorders, bone diseases, cancer, dementia, epilepsy, respiratory illness, rheumatic conditions, glaucoma, gout, iron deficiency, chronic pain, psychiatric diseases, use of antipsychotic drugs, thyroid disease, and other rare diseases. Thirdly, year of follow-up. Insurance characteristics were obtained from three of four insurers and considered in a sensitivity analysis.

For peer review only - http://bmjopen.bmj.com/site/about/guidelines.xhtml

Statistical analysis and estimation of AF costs per person

First, the characteristics of the included Swiss-AF and control patients were described with standard methods. Healthcare costs per patient and cost trajectories over time were descriptively analysed for both populations, distinguishing between total, outpatient, and inpatient costs. Cost trajectories over time were depicted as line plots not considering missing data points.

Second, the mentioned multivariable regression approaches were pursued to assess the cost impact of AF, using the above-listed covariates as independent variables. All approaches included a time fixed effect for month of observation.

The two-part alternatives to OLS were pursued because healthcare costs are characterised by a significant proportion of zero values and right-skewed distributions of non-zero costs.[25] In the first part of the two-part models, the probability of having any costs in a given year of follow-up was estimated using a logistic model. The same covariates were used in the second part of the model, estimating the costs conditional on having occurred. Again, OLS was chosen for the second part to achieve direct cost estimates. Alternatively, generalised linear models (GLMs) with an assumed gamma distribution and logarithmic link function were used in the second part, to better account for the heteroscedasticity typically present in healthcare costs.[26] The cost ratios of the GLM part were converted to marginal effects to enable a direct comparison with the OLS-based results. Mean annual costs were finally calculated by multiplying the predicted values of both modelling parts.[27] To estimate the marginal cost impact of AF, all patients were assumed to have AF, or not to have AF. Both sets of predicted values were calculated, and the difference was interpreted as the cost impact of AF.[28] A further analysis was run by estimating the AF costs with propensity score matching, using a 1:1 nearest neighbour approach. Given the characteristics of the data, the GLM-based two-part modelling approach was considered theoretically most suitable, and the corresponding results were treated as primary.

Third, the different regression-based estimates of AF costs were compared with the estimates of AF costs resulting from applying the AF adjudication algorithm to the Swiss-AF patients' claims data.[24]

AF costs at the national level

Fourth, cost of illness of AF for Switzerland was roughly approximated as total costs per year, and costs per inhabitant and year, for the time period 2000-2019. Mean annual AF-related costs were taken from the GLM-based two-part model and assumed to follow the trend of healthcare expenditures in Switzerland for the period (index 2019

For peer review only - http://bmjopen.bmj.com/site/about/guidelines.xhtml

= 100%). AF prevalence was taken from the data base of the Global Burden of Disease Project for the Swiss population older than 30.[2] For cost calculations per capita, the Swiss population size was used with no age restriction, obtained from the Swiss Federal Statistical Office[29].

All analyses were conducted using R V3.6.3.

Patient and public involvement

Patients or the public were not involved in the design, or conduct, or reporting, or dissemination plans of our research.

For peer review only

Results

Patient population

Figure 1 shows the cohort selection. Of 2,415 Swiss-AF patients, 1,024 (42.4%) had claims data available and were included in the analysis (patients without available claims data showed similar characteristics[24]). In the population-based reference sample, 16,556 individuals were classified as "AF not obvious" and included as controls. Baseline characteristics by cohort are shown in **Table 1**. The supplementary **Figure S1** provides details on the numbers of patients at risk, cumulative numbers of events, the development of costs and Kaplan-Meier survival estimates across the full observation period 2014-2020 by cohort.

Table 1. Baseline characteristics.

	**Swiss-AF**	**Controls**	
N	1 024	16 556	SMD
**Characteristics**			
Age *mean (SD)*	73.04 (8.17)	72.64 (8.52)	0.401
Sex male *N (%)*	741 (72.4)	11766 (71.1)	0.145
**Comorbidities (PCG)** ***N (%)***			
Acid related disorders	397 (38.8)	2802 (17.4)	0.326
Bone diseases	44 (4.3)	644 (4.0)	0.035
Cancer	35 (3.4)	510 (3.2)	0.067
Cardiovascular	754 (73.8)	10381 (63.7)	0.402
Dementia	27 (2.6)	797 (5.0)	0.097
Diabetes	122 (11.9)	2298 (14.3)	0.161
Epilepsy	66 (6.5)	982 (6.1)	0.077
Glaucoma	103 (10.1)	1634 (10.2)	0.035
Gout	96 (9.4)	935 (5.8)	0.151
Hyperlipidaemia	425 (41.6)	5649 (35.0)	0.174
Iron deficiency	66 (6.5)	567 (3.5)	0.116
Pain	386 (37.8)	2484 (15.4)	0.347
Psychiatric	266 (26.0)	2837 (17.6)	0.136
Antipsychotic	16 (1.6)	878 (5.5)	0.142
Respiratory	144 (14.1)	1915 (11.9)	0.141
Rheumatic conditions	406 (39.7)	3074 (19.1)	0.309
Thyroid disorders	87 (8.5)	908 (5.7)	0.083
Other rare diseases	27 (2.6)	696 (4.4)	0.107
Number of PCGs *mean (SD)*	3.39 (2.53)	2.41 (1.98)	0.31
**Socioeconomic**			
Mother tongue *N (%)*			0.108
German	755 (73.7)	12944 (78.2)	
French	141 (13.8)	1708 (10.3)	
Italian	128 (12.5)	1904 (11.5)	
**Greater Region** ***N (%)***			0.182
Zurich	125 (12.2)	2083 (12.6)	

For peer review only - http://bmjopen.bmj.com/site/about/guidelines.xhtml

Lake Geneva Region	56 (5.5)	1086 (6.6)	
Espace Mitelland	289 (28.2)	3702 (22.4)	
Northwestern Switzerland	310 (30.3)	5990 (36.2)	
Eastern Switzerland	67 (6.5)	944 (5.7)	
Southern Switzerland	125 (12.2)	1904 (11.5)	
Central Switzerland	52 (5.1)	847 (5.1)	

Abbreviations: AF: atrial fibrillation, PCG: pharmaceutical cost groups, SD: standard deviation, SMD: standardized mean difference.

Healthcare costs over time

The evolution of mean annual costs by cohort and cost component is depicted in **Figure 2** (details in **Table S3**, **Figure S2**). The unadjusted average total cost per patient and year amounted to CHF 19,037 (EUR 17,306) for Swiss-AF patients, around 1.7-fold more than for control patients. In both cohorts, inpatient and outpatient costs each contributed half of the total costs on average.

AF-related and non-AF related healthcare costs

Table 2 compares the model-based estimated differences in healthcare costs between AF patients and controls, interpreted as AF-related costs. Details for each model are in the supplement (**Tables S4-S7**). All estimates of AF-related costs were in a similar range. The GLM-based two-part model yielded total AF costs of CHF 5,588 (EUR 5,080) annually, while outpatient costs were CHF 1,425 (EUR 1,295), and inpatient costs CHF 2,779 (EUR 2,526).

Table 2. Estimates of difference in healthcare costs between AF patients and controls: comparison of alternative models.

Dependent variable		*Model*			
		**Two part GLM**	**Two part OLS**	**Propensity score matching**	**OLS**
**Total costs**	*Odds ratio (OR) (Logistic part)*	1.50 [1.46, 1.54]		–	–
	Marginal effect / Cost estimate (GLM / OLS part)	6 374 [5 609, 7 139]	5 743 [5 210, 6 277]	–	–
	Combined two part / direct estimate	5 588	5 187	5 692	5 124

For peer review only - http://bmjopen.bmj.com/site/about/guidelines.xhtml

**Outpatient costs**	*OR (Logistic part)*	1.46 [1.42, 1.50]		–	–
	Marginal effect / Cost estimate (GLM / OLS part)	1 299 [1 097, 1 501]	1 043 [860, 1 226]	–	–
	Combined two part / direct estimate	1 425	1 246	1 342	1 124
**Inpatient costs**	*OR (logistic part)*	1.13 [1.08, 1.17]		–	–
	Marginal effect / Cost estimate (GLM / OLS part)	35 154 [28 827, 41 481]	37 322 [32 916, 41 728]	–	–
	Combined two part / direct estimate	2 779	2 957	4 350	3 999

Notes: The two part models used a logistic regression in the first part, and GLM or OLS respectively in the second part. Propensity score matching was done 1:1, and OLS refers to a direct (non-two part) OLS estimate. The brackets show 95% confidence intervals. An exchange rate of EUR 1.0 = CHF 1.1 can be used to convert the costs into Euros to facilitate comparison with other countries. Abbreviations: GLM: generalised linear model, OLS: ordinary least squares regression, OR: odds ratio.

Figure 3 compares the estimates of AF-related costs from the GLM- and OLS-based two-part models with the estimates for the Swiss-AF patients based on the AF-adjudication algorithm without controls. The estimated AF-related costs were very similar for all three methods, ranging from CHF 5,187 (OLS-based) to CHF 5,588 (GLM-based), and CHF 5,679 (adjudication-based). AF-related costs from the adjudication algorithm are shown by subgroup, revealing details not available from the regression estimates: AF-treatment costs contributed most to AF-related costs, while the costs of AF-related complications contributed relatively little. Non-AF-related costs induced by diseases other than AF were similar across all approaches. They amounted to CHF 11,100 (EUR 10,091) per year OLS- and GLM-based, and CHF 13,400 (EUR 12,182) per year adjudication-based.

For peer review only

Cost of illness in Switzerland

Figure 4 shows the estimated evolution of AF-related costs at the Swiss national level, in total and in CHF per inhabitant. Since 2000 the increase in costs was faster than the prevalence increase of AF in the population. Estimates amounted to CHF 700 million (EUR 636 million) in 2019, equivalent to about CHF 80 per inhabitant. Male patients contributed 1.5 times more to the costs than female patients due to higher prevalence, and most of the costs were accrued in patients older than 70 years (supplementary **Figure S3**).

For peer review only

For peer review only - http://bmjopen.bmj.com/site/about/guidelines.xhtml

Discussion

This study presents up-to-date evidence of real-world AF-related healthcare costs. To the best of our knowledge, it is the first study comparing AF-related cost estimates using population-based controls with a data-derived bottom-up approach to adjudication of AF costs. We obtained similar results for all estimation methods used: mean annual AF-related costs amounted to CHF 5,600 (EUR 5,091); indicating roughly 50% higher direct medical costs of Swiss AF patients compared to the population-based controls. At the national level, AF-related costs amounted to CHF 700 million (EUR 636 million) in 2019, equivalent to about 1% of the Swiss healthcare expenditure.

Our estimates of AF-related direct medical costs of CHF 5,600 annually are consistent with previously published estimates, despite notable differences in study designs and data collection approaches. In Europe, annual direct medical cost estimates at the patient level ranged from EUR 2,315–3,785 (Spain EUR 2,315 (2006)[4], Germany EUR 2,405 (2005)[3], Sweden EUR 2,787 (2006)[3], Italy EUR 3,225 (2006)[4], France EUR 3,307 (2004)[6], Scotland GBP 3,785 (2015)[5]). After accounting for purchasing power parity (PPP), our estimate for Switzerland is still somewhat higher, but comparable. As Ringborg[4] has shown, differences within Europe are notable even after accounting for PPP, reflecting differences in the healthcare systems of the countries. Moreover, Switzerland is known to have a relatively more expensive healthcare system than other European countries.

Transferred to the Swiss national level, direct medical AF costs amounted to CHF 700 million in 2019. AF-related cost estimates for European countries ranged from EUR 660–2,548 million (Germany EUR 660 million (2004)[9], France EUR 1,942 million (2012)[10], Sweden EUR 240 million (2007)[11], United Kingdom GBP 244 million (1995) to model-based estimates of 2,548 million (2020)[12,15]). In the USA, AF-related costs were estimated to be around USD 6 billion (2008)[8,13]. It is difficult to compare the existing cost-of-illness studies due to methodological differences, while differences in their timing and in population size can e.g. be captured by expressing AF-related costs as a share of the gross domestic product (GDP) or total healthcare expenditure in the relevant year. In Switzerland, the estimated AF-related costs amounted to 0.1% of the GDP in 2019, equivalent to roughly 1% of the total healthcare expenditure. This is again comparable with the existing literature. In Portugal, AF-related costs were estimated to be 0.08% of the GDP, including indirect costs but excluding bleeding-related events and services.[30] AF-related cost estimates as a share of healthcare expenditures ranged from 0.28-1.7%:

For peer review only - http://bmjopen.bmj.com/site/about/guidelines.xhtml

Germany 0.28%[14], USA 0.42%[14], UK 0.62%[12], Australia 1.01%[14], UK based on modelling 0.91-1.62%[15], Denmark 1.7%[16].

Our estimates of AF-related costs in the large, prospective Swiss-AF cohort were highly consistent and robust. In particular, the regression-based estimates of AF costs using a matched control population were remarkably similar to the cost estimates based on direct adjudication to AF. The adjudication algorithm was derived using clinical and claims data for the Swiss AF sample only, without comparison to the population-based controls. So far, most literature has focussed on estimating costs from clinical or claims data[3,4,6,9,10,30]; only very few comparisons with a control population are available[8,16]. While lending strong credibility to our results, the observed similarity also suggests that lacking controls, the AF-related portion of healthcare costs may still be estimated quite accurately with a well-defined algorithm supported by clinical data.

There are still several limitations of our work requiring discussion. Most importantly, the Swiss-AF study population is not truly representative of all AF patients in Switzerland, given enrolment in in- and outpatient clinical centres and an expected under-representation of patients younger than 65 years driven by eligibility criteria. It would in fact be extremely difficult, if not impossible, to recruit a truly representative sample of AF patients into any study. We still expect our cost estimates to provide a reasonable approximation of the typical AF-related costs of Swiss patients with clinically diagnosed AF. The decision to enrol patients independently of time since diagnosis supports this notion, all the more given the observed high degree of stability of our results over time. However, we cannot exclude that enrolment of the Swiss-AF patients in clinical centres may have led to a certain overestimation of inpatient cost in the first year of observation. Second, the selection algorithm used to define the control population is likely to have missed some patients with AF. However, this should not have biased the results strongly, as these patients did not display indicators of AF-related hospitalization or major procedures. If anything, a moderate underestimation of AF costs may have occurred. Third, cost calculations were based on claims data, and not all claims may have been handed in for reimbursement. However, in patients with a chronic disease and substantial healthcare costs, this is rather not expected. We could not acquire insurance characteristics from one insurer and have consider these in a sensitivity analysis without distortion of our results. Fourth, the controls were provided by one health insurance only. Major differences between insurers are not expected in the Swiss statutory health insurance, as the primary benefit package is uniform across the country and defined by law. A further limitation affects the estimation of the cost-of-illness at the

For peer review only - http://bmjopen.bmj.com/site/about/guidelines.xhtml

national level. There were several assumptions made: a) AF-related cost estimates were based on the results of the GLM-based two part model, b) the development of costs per patient over time was assumed to follow the development of healthcare expenditures in Switzerland, and c) AF patients under the age of 30 were not considered in the prevalence estimates. As a last limitation, this analysis focused on direct medical costs from the perspective of the Swiss statutory health insurance. Costs of lost productivity were not considered and the total impact of AF on the economy was thus not captured. Separate work will address the topic of impact of AF on productivity in younger Swiss-AF patients.

In conclusion, the results of this study indicate that AF patients incur 50% higher costs than comparable population-based controls. Costs were at a comparable level as reported by other cost-of-illness studies for AF. Different regression-based approaches to estimating AF-related costs led to similar results, confirming the robustness of our findings. A well-defined bottom-up approach using clinical and claims data but no control population also yielded similar results. This finding is valuable for the interpretation of the existing cost-of-illness literature and may inform decisions on investments in healthcare policies.

For peer review only

Funding

This work is supported by grants of the Swiss National Science Foundation (grant numbers 105318_189195 / 1, 33CS30_148474, 33CS30_177520, 32473B_176178, and 32003B_197524), the Swiss Heart Foundation, the Foundation for Cardiovascular Research Basel (FCVR), and the University of Basel.

Acknowledgements

We thank Lukas Kauer from the health insurance CSS, Pascal Godet from the health insurance Sanitas, Andri Signorell from the health insurance Helsana, and the health insurance KPT for providing the claims data for this study.

Contributors

All authors certify that they have participated sufficiently in the work to take public responsibility for the content, including participation in the concept, design, analysis, writing, or revision of the manuscript.

Disclosures

Prof. Beer reports grant support from the Swiss National Foundation of Science, The Swiss Heart Foundation and the Stiftung Kardio; grant support, speakers- and consultation fees to the institution from Bayer, Sanofi and Daichii Sankyo. Dr Blozik reports grants from Swiss Cancer Research Foundation; institutional grants from Amgen, MSD, Novartis, Pfizer, all outside the submitted work. Dr Bonati reports personal fees and nonfinancial support from Amgen, grants from AstraZeneca, personal fees and nonfinancial support from Bayer, personal fees from Bristol-Myers Squibb, personal fees from Claret Medical, grants from Swiss National Science Foundation, grants from University of Basel, grants from Swiss Heart Foundation, outside the submitted work. Dr Conen received consulting fees from Roche Diagnostics, and speaker fees from Servier and BMS/Pfizer, all outside of the current work. Dr Kühne reports personal fees from Bayer, personal fees from Böhringer Ingelheim, personal fees from Pfizer BMS, personal fees from Daiichi Sankyo, personal fees from Medtronic, personal fees from Biotronik, personal fees from Boston Scientific, personal fees from Johnson&Johnson, personal fees from Roche, grants from Bayer, grants from Pfizer, grants from Boston Scientific, grants from BMS, grants from Biotronik, grants from Daiichi Sankyo. Dr Moschovitis has received consultant fees for taking part to advisory boards from Novartis, Boehringer Ingelheim, Bayer, Astra Zeneca and Daiichi Sankyo, all outside of the presented work. Dr Müller reports fellowship and training support from Biotronik, Boston Scientific, Medtronic, Abbott/St. Jude Medical, and Biosense Webster; speaker honoraria from

For peer review only

Biosense Webster, Medtronic, Abbott/St. Jude Medical, AstraZeneca, Daiichi Sankyo, Biotronik, MicroPort, Novartis, and consultant honoraria for Biosense Webster, Medtronic, Abbott/St. Jude Medcal, and Biotronik. Dr Osswald received research grants from the Swiss National Science Foundation and from the Swiss Heart Foundation, research grants from Foundation for CardioVascular Research Basel, research grants from Roche, educational and speaker office grants from Roche, Bayer, Novartis, Sanofi AstraZeneca, Daiichi-Sankyo and Pfizer. Dr Reichlin has received research grants from the Swiss National Science Foundation, the Swiss Heart Foundation, and the sitem insel support fund, all for work outside the submitted study. Speaker/consulting honoraria or travel support from Abbott/SJM, Astra Zeneca, Brahms, Bayer, Biosense-Webster, Biotronik, Boston-Scientific, Daiichi Sankyo, Medtronic, Pfizer-BMS and Roche, all for work outside the submitted study. Support for his institution's fellowship program from Abbott/SJM, Biosense-Webster, Biotronik, Boston-Scientific and Medtronic for work outside the submitted study. Dr Schwenkglenks reports grants from Swiss National Science Foundation, for the conduct of the study; grants and personal fees from Amgen, grants from MSD, grants from Novartis, grants from Pfizer, grants from The Medicines Company, all outside the submitted work. Dr Serra-Burriel reports grants from the European Commission outside of the present work. Dr Sticherling has received speaker honoraria from Biosense Webster and Medtronic and research grants from Biosense Webster, Daiichi-Sankyo, and Medtronic. The remaining authors have nothing to disclose.

Ethics

The Swiss-AF study protocol was approved by the local ethics committee (Ethikkommission Nordwest- und Zentralschweiz, 2014-067, PB_2016-00793), and written informed consent was obtained from each participant.

The population-based reference data set from Helsana was provided anonymously based on a waiver provided by the competent ethics committee (Kantonale Ethikkommission Zürich, 2020-01346).

Data availability

The Swiss-AF patient informed consent forms state that the data, containing personal and medical information, are exclusively available for research institutions in an anonymized form and are not allowed to be made publicly available. Researchers interested in obtaining the Swiss-AF data for research purposes can contact the Swiss-AF scientific lead. Contact information is provided on the Swiss-AF website

For peer review only - http://bmjopen.bmj.com/site/about/guidelines.xhtml

(http://www.swissaf.ch/contact.htm). Authorization of the responsible ethics committee is mandatory before the requested data can be transferred to external research institutions.

The population-based reference data set from Helsana was provided anonymously. These claims data cannot be shared publicly because they are the property of Helsana. Considering SNSF policies encouraging data sharing, the data may be shared via Helsana with scientific institutions under specific conditions and considering all data protection rules; final decisions are taken by Helsana. Any such sharing would also require ethical clarification of responsibility and/or clearance, as applicable.

For peer review only

References

1. Velleca, M., Costa, G., Goldstein, L., Bishara, M. & Ming Boo, L. *A Review of the Burden of Atrial Fibrillation: Understanding the Impact of the New Millennium Epidemic across Europe*. *EMJ EUROPEAN MEDICAL JOURNAL* vol. 110 (2019).
2. Global Burden of Disease (GBD 2019) | Institute for Health Metrics and Evaluation. https://www.healthdata.org/gbd/2019.
3. Jönsson, L., Eliasson, Å., Kindblom, J., Almgren, O. & Edvardsson, N. Cost of illness and drivers of cost in atrial fibrillation in Sweden and Germany. *Appl Health Econ Health Policy* 8, 317–325 (2010).
4. Ringborg, A. *et al.* Costs of atrial fibrillation in five European countries: Results from the Euro Heart Survey on atrial fibrillation. *Europace* 10, 403–411 (2008).
5. Ciminata, G., Geue, C., Langhorne, P. & Wu, O. A two-part model to estimate inpatient, outpatient, prescribing and care home costs associated with atrial fibrillation in Scotland. *BMJ Open* 10, e028575 (2020).
6. le Heuzey, J.-Y. *et al.* Cost of care distribution in atrial fibrillation patients: the COCAF study. *Am Heart J* 147, 121–126 (2004).
7. Patel, N. J. *et al.* Contemporary Trends of Hospitalization for Atrial Fibrillation in the United States, 2000 Through 2010. *Circulation* 129, 2371–2379 (2014).
8. Kim, M., Johnston, S., Chu, B., Dalal, M. R. & Schulman, K. Estimation of total incremental health care costs in patients with atrial fibrillation in the United States. *Circ Cardiovasc Qual Outcomes* 4, 313–320 (2011).
9. McBride, D., Mattenklotz, A. M., Willich, S. N. & Brüggenjürgen, B. The costs of care in atrial fibrillation and the effect of treatment modalities in Germany. *Value in Health* 12, 293–301 (2009).
10. Cotté, F. E. *et al.* Burden of stroke and other cardiovascular complications in patients with atrial fibrillation hospitalized in France. *Europace* 18, 501 (2016).
11. Ericson, L., Bergfeldt, L. & Björholt, I. Atrial fibrillation: the cost of illness in Sweden. *The European Journal of Health Economics* 12, 479–487 (2010).
12. Stewart, S., Murphy, N. F., Walker, A., McGuire, A. & McMurray, JJ. Cost of an emerging epidemic: an economic analysis of atrial fibrillation in the UK. *Heart* 90, 286–292 (2004).
13. Coyne, K. S. *et al.* Assessing the direct costs of treating nonvalvular atrial fibrillation in the United States. *Value in Health* 9, 348–356 (2006).
14. Ball, J., Carrington, M. J., McMurray, J. J. V. & Stewart, S. Atrial fibrillation: Profile and burden of an evolving epidemic in the 21st century. *Int J Cardiol* 167, 1807–1824 (2013).

For peer review only - http://bmjopen.bmj.com/site/about/guidelines.xhtml

15. Burdett, P. & Lip, G. Y. H. Atrial fibrillation in the UK: predicting costs of an emerging epidemic recognizing and forecasting the cost drivers of atrial fibrillation-related costs. *Eur Heart J Qual Care Clin Outcomes* 0, 1–8 (2020).

16. Johnsen, S. P., Dalby, L. W., Täckström, T., Olsen, J. & Fraschke, A. Cost of illness of atrial fibrillation: a nationwide study of societal impact. *BMC Health Serv Res* 17, 1–8 (2017).

17. Conen, D. *et al.* Design of the Swiss Atrial Fibrillation Cohort Study (Swiss-AF): structural brain damage and cognitive decline among patients with atrial fibrillation. *Swiss Med Wkly* 147, (2017).

18. ICD. https://www.who.int/standards/classifications/classification-of-diseases (2021).

19. SwissDRG. https://www.swissdrg.org/de/akutsomatik/swissdrg-system-1002021 (2021).

20. CHOP. https://www.bfs.admin.ch/bfs/de/home/statistiken/kataloge-datenbanken/publikationen.assetdetail.9286150.html (2021).

21. ATC. https://www.swissmedic.ch/swissmedic/en/home/services/listen_neu.html (2021).

22. TARMED. https://www.bag.admin.ch/bag/de/home/versicherungen/krankenversicherung/krankenversicherung-leistungen-tarife/Aerztliche-Leistungen-in-der-Krankenversicherung/Tarifsystem-Tarmed.html (2021).

23. Huber, C. A., Szucs, T. D., Rapold, R. & Reich, O. Identifying patients with chronic conditions using pharmacy data in Switzerland: an updated mapping approach to the classification of medications. *BMC Public Health* 13, 1–10 (2013).

24. Aebersold, H. *et al.* Patient clusters and cost trajectories in the Swiss Atrial Fibrillation cohort. *Heart* heartjnl-2022-321520 (2022) doi:10.1136/HEARTJNL-2022-321520.

25. Belotti, F., Deb, P., Manning, W. G. & Norton, E. C. Twopm: Two-Part Models: *https://doi.org/10.1177/1536867X1501500102* 15, 3–20 (2015).

26. Deb, P. & Norton, E. C. Modeling Health Care Expenditures and Use. *Annu Rev Public Health* 39, 489–505 (2018).

27. Blough, D. K., Madden, C. W. & Hornbrook, M. C. Modeling risk using generalized linear models. *J Health Econ* 18, 153–171 (1999).

28. Smith, V. A., Neelon, B., Maciejewski, M. L. & Preisser, J. S. Two parts are better than one: modeling marginal means of semicontinuous data. *Health Serv Outcomes Res Methodol* 17, 198–218 (2017).

For peer review only

29. Bundesamt für Statistik | Bundesamt für Statistik. https://www.bfs.admin.ch/bfs/de/home.html.

30. Gouveia, M. *et al.* Burden of disease and cost of illness of atrial fibrillation in Portugal. *Cardiologia* 34, 1–11 (2015).

For peer review only

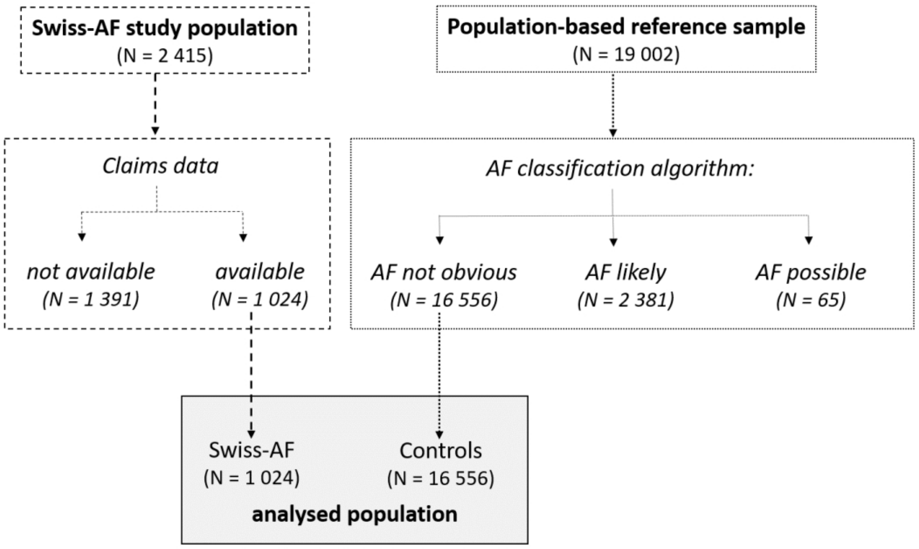

659x401mm (59 x 59 DPI)

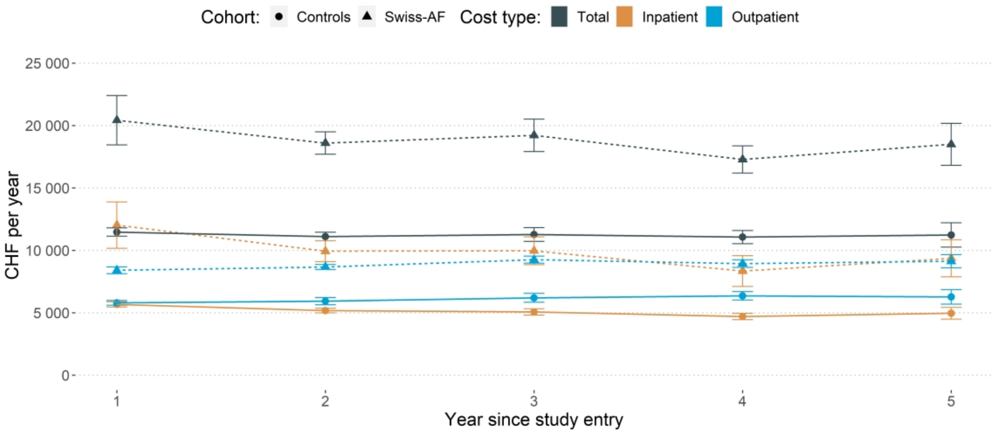

349x159mm (600 x 600 DPI)

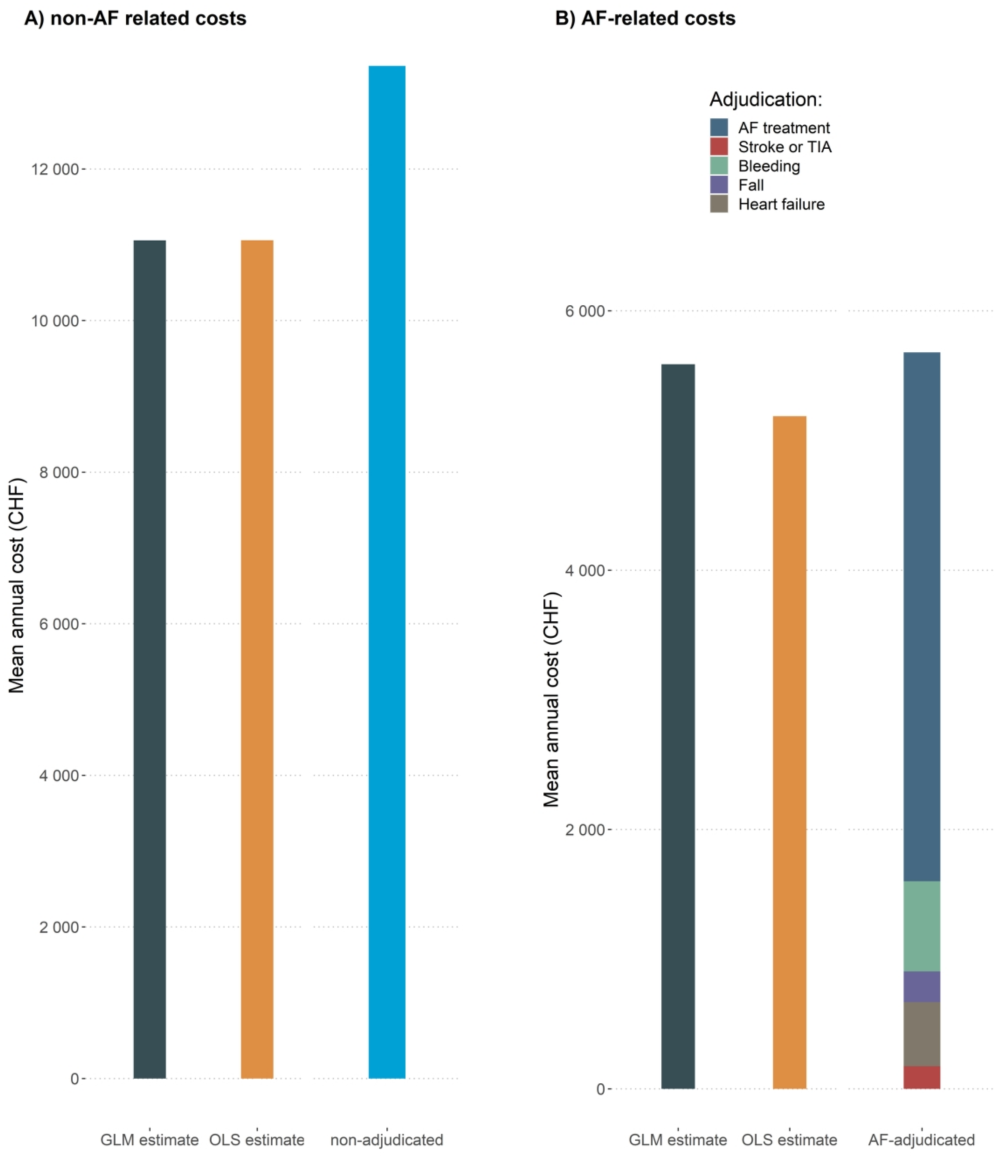

84x99mm (600 x 600 DPI)

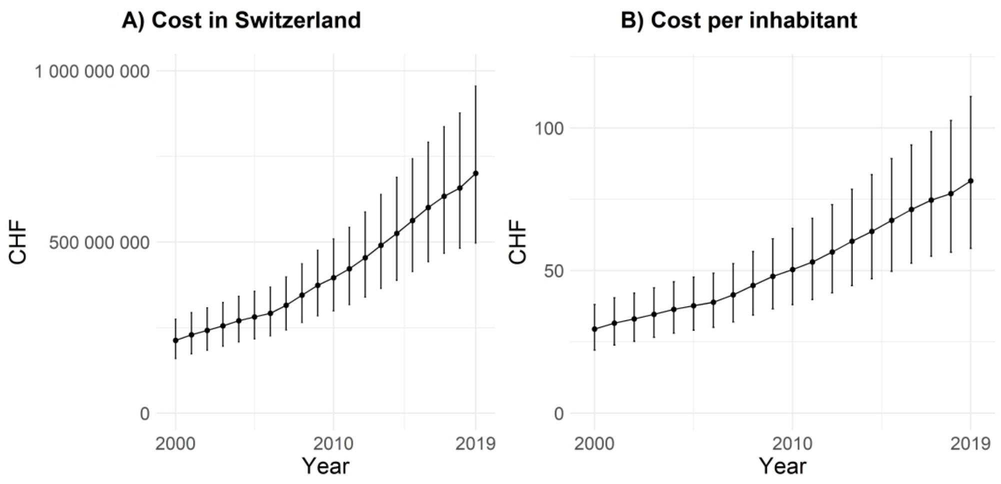

279x134mm (600 x 600 DPI)

Table S1. Sample size determination for the population-based control sample

In the absence of exact solutions for the determination of the required size of the population-based, non-AF control sample, we tried to estimate a plausible magnitude based on published cost studies. Our study aim was to compare the costs of prevalent atrial fibrillation (AF) patients with the costs of controls not having AF. In the absence of published comparisons of this type, we used the AF-attributable versus non-AF-attributable costs of AF patients as a fallback. We found that mean attributable costs may differ by roughly 0.3 standard deviations from the mean of non-attributable costs [1, 2, 3]. Based on the results of Turakhia [4], an expected minimum effect size (Cohen's d) of the cost of AF would be approximately 0.1. Given the possibility of such a small effect size and to be on the safe side we assumed a 50% smaller effect, i.e. Cohen's d of 0.05.

AF patients were planned to be compared to controls differing in several dimensions, and a variety of sub-analyses were planned to be performed to characterize the cost impact of AF. To mimic the impact of this situation on the required size of the control sample, a Bonferroni correction for multiple comparisons was assumed, with an estimated number of 15 hypothesis: checking for divergences in gender, age, accumulation of costs over time in different subgroups, various types of costs etc.

With a standard statistical power function and assuming the parameter values and corrections explained above (Cohen's d = 0.05, number of hypotheses = 15), a sample size of 17'000 valid controls was estimated to be required to obtain a 95% statistical power and a 5% false positive risk. This became the planned size of the non-AF control sample. Considering that some otherwise eligible people would have AF, the size of the full reference sample was inflated to the point where 17'000 non-AF controls were reached.

[1] Jönsson et al. 2010. Cost of Illness and drivers of Cost in Atrial Fibrillation in Sweden and Germany, Applied Health Economics and Health Policy, 8, 317-325, DOI: 10.2165/11319880-000000000-00000

[2] Brüggenjürgen et al. 2007, The Impact of Atrial Fibrillation on the Cost of Stroke: the Berlin Acute Stroke Study, Value in Health, 10:2, 137-143, DOI: 10.1111/j.1524-4733.2006.00160.x

[3] Wodchis et al. 2012. A Review of the Cost of Atrial Fibrillation, Value in Health, 15:2, 240-248, DOI: https://doi.org/10.1016/j.jval.2011.09.009

[4] Turakhia et al. 2015. Economic Burden of Undiagnosed Nonvalvular Atrial Fibrillation in the United States, Am J Cariol, 116:5, 733-739, DOI: 10.1016/j.amjcard.2015.05.045.

For peer review only

Table S2. Algorithm classifying the population-based reference sample as “AF likely” and “AF possible”. Individuals with none of the listed criteria present were classified as “AF not obvious” and considered as controls.

code		allocation	
		AF likely	AF possible
ICD10	**I48.0** Vorhofflimmern, paroxysmal	1	
ICD10	**I48.1** Vorhofflimmern, persistierend	1	
ICD10	**I48.2** Vorhofflimmern, permanent	1	
ICD10	**I48.3** Vorhofflattern, typisch	1	
ICD10	**I48.4** Vorhofflattern, atypisch	1	
ICD10	**I48.9** Vorhofflimmern und Vorhofflattern, nicht näher bezeichnet	1	
ICD10	**I49.8** Sonstige näher bezeichnete kardiale Arrhythmien		1
ICD10	**I49.9** Kardiale Arrhythmie, nicht näher bezeichnet		1
DRG	**F50A** Ablative Massnahmen bei Tachyarrhythmie mit bestimmter Ablation und komplexem Eingriff, Alter < 16 Jahre		1
DRG	**F50D** Ablative Massnahmen bei Tachyarrhythmie, Alter > 15 Jahre		1
ICD10 + DRG	ICD **I48 +** DRG **F50A**	1	
ICD10 + DRG	ICD **I48 +** DRG **F50D**	1	
CHOP	**Z37.34.24** Lokalisationen bei Ablationsverfahren bei Tachyarrhythmien	1	
CHOP	**Z99.61** Vorhofskardioversion	1	
CHOP	**Z99.62** Externe Kardioversion	1	
Tarmed	**17.1510** Kardioversion bei Vorhofflimmern/Vorhofflattern, als alleinige Leistung	1	
ATC	**C01BD07** Dronedarone (Multaq)	1	

Notes: Abbreviations: AF: atrial fibrillation, ATC: anatomical therapeutic chemical classification, CHOP: Swiss invasive medical procedures catalogue, DRG: diagnosis related group, ICD10: international classification of diseases (10th revision).

For peer review only

For peer review only - http://bmjopen.bmj.com/site/about/guidelines.xhtml

Figure S1. Trajectories of monthly costs and Kaplan-Meier curves by cohort.

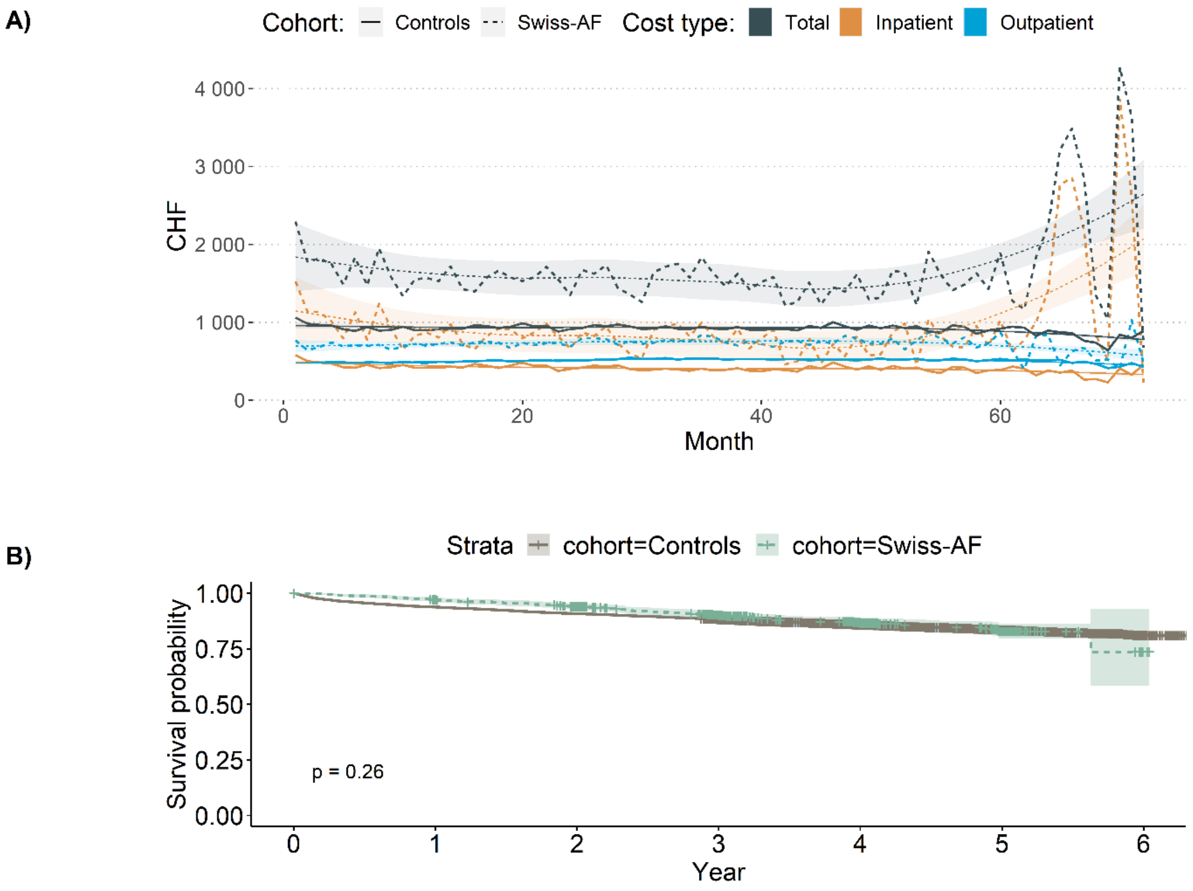

C) N at risk

Number at risk

Strata	0	1	2	3	4	5	6
cohort=Controls	16556	15532	15041	14279	8714	4299	385
cohort=Swiss-AF	1024	982	920	706	375	136	2

Year

D) deaths

Cumulative number of events

Strata	0	1	2	3	4	5	6
cohort=Controls	5	1026	1515	1939	2271	2439	2504
cohort=Swiss-AF	0	29	60	96	117	126	127

Year

E) loss to FU

Cumulative number of events

Strata	0	1	2	3	4	5	6
cohort=Controls	0	0	0	0	0	0	0
cohort=Swiss-AF	7	14	23	28	34	37	39

Year

F) admin. censoring

Cumulative number of events

Strata	0	1	2	3	4	5	6
cohort=Controls	0	0	0	357	5572	9844	13675
cohort=Swiss-AF	0	0	26	212	505	735	856

Year

Notes: median (interquartile range IQR) follow-up: Swiss-AF 3.41 (1.08) years, controls 4.10 (1.72) years; total patient-years of follow-up: SAF 3 571.24, cohort 66 068.24.

Table S3. Observed annual costs in CHF by cost component and cohort.

	Swiss-AF		***Controls***	
Cost component	*Median [IQR]*	*Mean (SD)*	*Median [IQR]*	*Mean (SD)*
Total	4 518 [825, 11 771]	19 037 (59 998)	2 135 [0, 7 473]	11 192 (38 939)
Total inpatient	0 [0, 0]	10 235 (56 327)	0 [0, 0]	5 077 (34 925)
Total outpatient drugs	508 [0, 29 56]	2 495 (7 382)	235 [0, 1 781]	1 984 (7 852)
Total outpatient without drugs	2 282 [59, 7 225]	6 307 (13 154)	801 [0, 4 310]	4 131 (10 260)
Total AF-adj.	400 [0, 3 213]	5 679 (36 135)	*NA*	*NA*
Total AF-adj. inpatient	0 [0, 0]	3 458 (35 188)	*NA*	*NA*
Total AF-adj. outpatient drugs	0 [0, 250]	591 (1 392)	*NA*	*NA*
Total AF-adj. outpatient without drugs	0 [0, 1 251]	1 630 (6 899)	*NA*	*NA*
AF-adjudication:				
Total AF treatment	226 [0, 2 773]	4 078 (2 8640)	*NA*	*NA*
Total stroke or TIA	0 [0, 0]	174 (9124)	*NA*	*NA*
Total bleeding	0 [0, 0]	696 (17462)	*NA*	*NA*
Total fall	0 [0, 0]	237 (4434)	*NA*	*NA*
Total heart failure	0 [0, 0]	494 (8469)	*NA*	*NA*

Abbreviations: adj.: adjudicated, AF: atrial fibrillation, IQR: interquartile range, PCG: pharmaceutical cost groups, SD: standard deviation, SMD: standardized mean difference.

For peer review only

Figure S2. Boxplot distribution of mean annual costs by cost outcome and cohort.

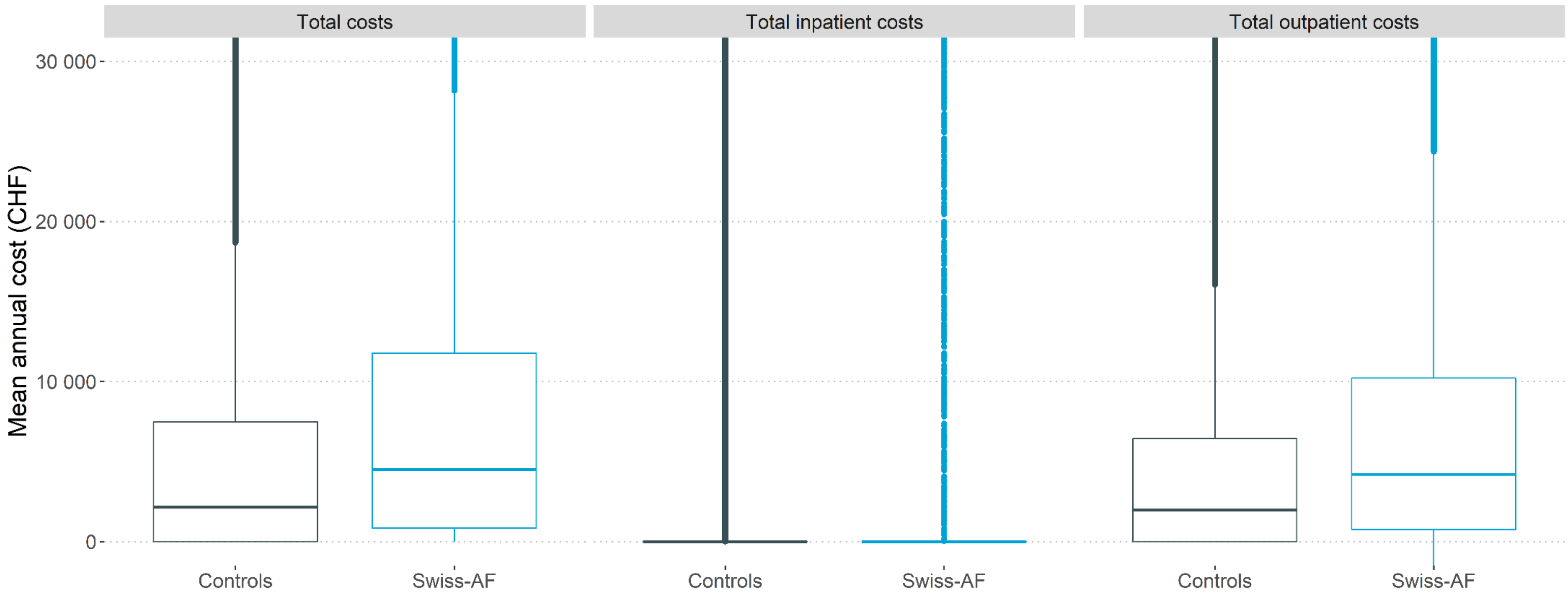

only

Table S4. Regression results from GLM-based two part modelling.

	Total costs		Outpatient costs		Inpatient costs	
	Odds ratio (Logistic part)	*Marginal effect (GLM part)*	*Odds ratio (Logistic part)*	*Marginal effect (GLM part)*	*Odds ratio (Logistic part)*	*Marginal effect (GLM part)*
Cohort: Swiss-AF	1.5 [1.46, 1.54]	6 374 [5 609, 7 139]	1.46 [1.42, 1.5]	1 299 [1 097, 1 501]	1.13 [1.08, 1.17]	35 154 [28 827, 41 481]
Month	1.00 [1.00, 1.00]	29 [20, 38]	1.00 [1.00, 1.00]	35 [33, 38]	1.00 [1.00, 1.00]	- 335 [- 408, -262]
Age	1.03 [1.03, 1.03]	242 [223, 260]	1.03 [1.02, 1.03]	36 [30, 41]	1.09 [1.09, 1.09]	- 3 075 [- 3 244, - 2 906]
Sex: Male	0.92 [0.91, 0.93]	3 254 [2 963, 3 545]	0.92 [0.91, 0.93]	1 485 [1 393, 1 577]	1.05 [1.02, 1.07]	10 449 [8 031, 12 866]
PCG acid related disorders	1.69 [1.66, 1.72]	2 610 [2 231, 2 989]	1.66 [1.63, 1.69]	1 487 [1 367, 1 606]	1.30 [1.27, 1.34]	- 3 545 [- 6 209, - 882]
PCG bone diseases	1.94 [1.87, 2.01]	5 278 [4 418, 6 138]	1.91 [1.84, 1.98]	4 517 [4 214, 4 821]	0.97 [0.93, 1.02]	18 455 [12 539, 24 372]
PCG cancer	2.12 [2.03, 2.21]	16 094 [14 613, 17 575]	2.09 [2, 2.18]	12 834 [12 269, 13 399]	1.22 [1.16, 1.29]	18 812 [11 626, 25 999]
PCG cardio	1.89 [1.87, 1.91]	- 402 [- 738, - 66]	1.90 [1.87, 1.92]	317 [214, 419]	0.98 [0.96, 1.01]	8 045 [5 312, 10 779]
PCG dementia	2.05 [1.98, 2.13]	1 819 [1 166, 2 471]	1.93 [1.87, 2]	949 [745, 1 154]	2.14 [2.07, 2.22]	- 26 586 [- 29 160, - 24 012]
PCG diabetes	1.67 [1.64, 1.7]	3 790 [3 355, 4 225]	1.66 [1.63, 1.69]	2 220 [2 083, 2 358]	1.25 [1.21, 1.28]	5 212 [1 986, 8 439]
PCG epilepsy	2.26 [2.18, 2.34]	5 403 [4 703, 6 103]	2.19 [2.12, 2.27]	2 636 [2 421, 2 851]	1.58 [1.53, 1.63]	- 3 375 [- 6 723, - 28]
PCG glaucoma	1.56	- 493	1.56	638	0.87	- 1 171

For peer review

	[1.53, 1.59]	[- 902, - 83]	[1.53, 1.59]	[500, 776]	[0.85, 0.9]	[- 4 610, 2 268]
PCG gout	1.35<br>[1.32, 1.39]	1 296<br>[713, 1 878]	1.36<br>[1.32, 1.39]	509<br>[329, 688]	1.02<br>[0.98, 1.06]	7 684<br>[27 98, 12 571]
PCG hyperlipidemia	1.32<br>[1.3, 1.33]	- 1 142<br>[- 1 437, - 847]	1.32<br>[1.3, 1.33]	- 275<br>[- 368, - 182]	0.86<br>[0.84, 0.88]	5 201<br>[2 606, 7 796]
PCG iron deficiency	1.57<br>[1.51, 1.64]	5 284<br>[4 401, 6 167]	1.56<br>[1.5, 1.62]	3 695<br>[3 401, 3 989]	1.20<br>[1.15, 1.25]	2 373<br>[- 2 429, 7 176]
PCG pain	1.47<br>[1.44, 1.5]	6 097<br>[5 628, 6 567]	1.45<br>[1.42, 1.49]	2 326<br>[2 186, 2 465]	1.82<br>[1.78, 1.87]	- 3 500<br>[- 6 189, - 812]
PCG psychiatric	2.19<br>[2.15, 2.24]	1 909<br>[1 534, 2285]	2.17<br>[2.12, 2.21]	1 048<br>[930, 1 167]	1.60<br>[1.56, 1.64]	- 15 150<br>[- 17 606, - 12 693]
PCG antipsychotic	2.92<br>[2.78, 3.06]	9 281<br>[8278, 1 0285]	2.50<br>[2.39, 2.62]	1 347<br>[1 108, 1 586]	5.98<br>[5.8, 6.17]	- 39 595<br>[- 41 844, - 37 345]
PCG respiratory	1.67<br>[1.64, 1.7]	2 124<br>[1 689, 2 558]	1.66<br>[1.63, 1.69]	1 192<br>[1 055, 1 328]	1.09<br>[1.06, 1.12]	9 644<br>[6 096, 13 193]
PCG rheumatic conditions	1.64<br>[1.61, 1.66]	- 1<br>[- 332, 330]	1.64<br>[1.62, 1.67]	458<br>[352, 564]	0.85<br>[0.82, 0.87]	8 762<br>[5 847, 11 677]
PCG thyroid disorders	1.47<br>[1.43, 1.51]	- 306<br>[- 850, 237]	1.47<br>[1.43, 1.51]	454<br>[273, 635]	0.90<br>[0.87, 0.94]	2 587<br>[- 2 050, 7 223]
PCG other rare diseases	2.26<br>[2.17, 2.35]	4 675<br>[3 889, 5 462]	2.22<br>[2.14, 2.3]	3022<br>[2 766, 3 277]	1.54<br>[1.49, 1.6]	- 1 274<br>[- 5 112, 2 564]
Urbanisation: agglomeration	0.97<br>[0.96, 0.99]	- 115<br>[- 436, 206]	0.98<br>[0.97, 0.99]	- 154<br>[- 256, - 53]	0.98<br>[0.96, 1]	1 242<br>[- 1 460, 3 945]
Urbanisation: rural	0.91<br>[0.9, 0.92]	- 44<br>[- 461, 374]	0.91<br>[0.9, 0.92]	- 307<br>[- 437, - 177]	1.13<br>[1.09, 1.16]	- 8 008<br>[- 11 371, - 4 645]
Greater Region: Lake Geneva	1.2<br>[1.17, 1.23]	2 819<br>[2 116, 3 523]	1.19<br>[1.16, 1.22]	2 131<br>[1 899, 2 362]	1.01<br>[0.97, 1.06]	18 469<br>[13 431, 23 508]
	1.07	- 771	1.06	- 499	0.82	8 683

For peer review only

Greater Region: Espace Mittelland	[1.05, 1.09]	[- 1 247, - 295]	[1.04, 1.08]	[- 650, - 348]	[0.79, 0.85]	[4 836, 12 529]
Greater Region: Northwestern Switzerland	1.1<br>[1.09, 1.12]	656<br>[217, 1 096]	1.10<br>[1.09, 1.12]	- 134<br>[- 272, 3]	0.95<br>[0.92, 0.98]	11 761<br>[8 460, 15 063]
Greater Region: Eastern Switzerland	0.93<br>[0.91, 0.96]	- 504<br>[- 1 182, 173]	0.94<br>[0.91, 0.96]	- 900<br>[- 1 105, - 695]	0.97<br>[0.92, 1.02]	6 304<br>[747, 11 861]
Greater Region: Southern Switzerland	1.26<br>[1.24, 1.29]	-720<br>[- 1 247, - 193]	1.26<br>[1.24, 1.29]	241<br>[68, 414]	0.67<br>[0.64, 0.7]	20 870<br>[15 771, 25 969]
Greater Region: Central Switzerland	0.94<br>[0.91, 0.96]	- 676<br>[- 1 379, 26]	0.93<br>[0.91, 0.95]	- 237<br>[- 463, - 11]	0.91<br>[0.86, 0.96]	- 3 079<br>[- 8 627, 2 470]
Observations	798 940	545 385	798 940	543 131	798 940	47 028

Notes: The brackets show 95% confidence intervals. Abbreviations: AF: atrial fibrillation, GLM: generalized linear model, PCG: pharmaceutical cost groups.

For peer review only

Table S5. Regression results from OLS-based two part modelling.

	Total costs		Outpatient costs		Inpatient costs	
	Odds ratio (logistic part)	*Cost estimate (OLS part)*	*Odds ratio (logistic part)*	*Cost estimate (OLS part)*	*Odds ratio (logistic part)*	*Cost estimate (OLS part)*
Cohort: Swiss-AF	1.5 [1.46, 1.54]	5 744 [5 210, 6 277]	1.46 [1.42, 1.5]	1 043 [860, 1 226]	1.13 [1.08, 1.17]	37 322 [32 916, 41 728]
Month	1.00 [1.00, 1.00]	5.47 [- 2.49, 13.43]	1.00 [1.00, 1.00]	26.33 [23.61, 29.06]	1.00 [1.00, 1.00]	- 330.16 [- 397.79, -262.52]
Age	1.03 [1.03, 1.03]	208 [191, 225]	1.03 [1.02, 1.03]	12 [6, 17]	1.09 [1.09, 1.09]	- 2 833 [- 2 975, - 2 690]
Sex: Male	0.92 [0.91, 0.93]	3 378 [3 090, 3 666]	0.92 [0.91, 0.93]	1 802 [1 703, 1 901]	1.05 [1.02, 1.07]	9 021 [6 663, 11 379]
PCG acid related disorders	1.69 [1.66, 1.72]	2 568 [2 239, 2 896]	1.66 [1.63, 1.69]	1 454 [1 341, 1 566]	1.30 [1.27, 1.34]	- 1 799 [- 4 324, 726]
PCG bone diseases	1.94 [1.87, 2.01]	6 789 [6 167, 7 411]	1.91 [1.84, 1.98]	5 529 [5 316, 5 742]	0.97 [0.93, 1.02]	13 650 [9 006, 18 294]
PCG cancer	2.12 [2.03, 2.21]	17 579 [16 855, 18 302]	2.09 [2, 2.18]	14 032 [13 784, 14 279]	1.22 [1.16, 1.29]	15 126 [9 514, 20 738]
PCG cardio	1.89 [1.87, 1.91]	- 325 [- 633, - 16]	1.90 [1.87, 1.92]	339 [234, 445]	0.98 [0.96, 1.01]	7 699 [5 040, 10 358]
PCG dementia	2.05 [1.98, 2.13]	1 897 [1 343, 2 451]	1.93 [1.87, 2]	869 [679, 1 060]	2.14 [2.07, 2.22]	- 23 773 [- 26 889, - 20 657]
PCG diabetes	1.67 [1.64, 1.7]	3 847 [3 497, 4 198]	1.66 [1.63, 1.69]	2 435 [2 315, 2 555]	1.25 [1.21, 1.28]	5 543 [2 641, 8 446]
PCG epilepsy	2.26 [2.18, 2.34]	6 908 [6 395, 7 421]	2.19 [2.12, 2.27]	3 450 [3 274, 3 626]	1.58 [1.53, 1.63]	- 8 073 [- 11 290, - 4 856]
PCG glaucoma	1.56	- 638	1.56	541	0.87	- 832

For peer review only

	[1.53, 1.59]	[- 1 028, - 249]	[1.53, 1.59]	[407, 674]	[0.85, 0.9]	[- 4 077, 2 413]
PCG gout	1.35<br>[1.32, 1.39]	1 766<br>[1 258, 2 275]	1.36<br>[1.32, 1.39]	806<br>[632, 980]	1.02<br>[0.98, 1.06]	8 036<br>[3 787, 12 285]
PCG hyperlipidemia	1.32<br>[1.3, 1.33]	- 1 269<br>[- 1 545, - 992]	1.32<br>[1.3, 1.33]	- 493<br>[- 588, - 398]	0.86<br>[0.84, 0.88]	4 874<br>[2 483, 7 266]
PCG iron deficiency	1.57<br>[1.51, 1.64]	6 803<br>[6 164, 7 442]	1.56<br>[1.5, 1.62]	4 636<br>[4 417, 4 855]	1.20<br>[1.15, 1.25]	1 784<br>[- 2 597, 6 165]
PCG pain	1.47<br>[1.44, 1.5]	6 773<br>[6 412, 7 134]	1.45<br>[1.42, 1.49]	2 817<br>[2 693, 2 941]	1.82<br>[1.78, 1.87]	- 2 141<br>[- 4 690, 407]
PCG psychiatric	2.19<br>[2.15, 2.24]	2 214<br>[1 883, 2 544]	2.17<br>[2.12, 2.21]	1 306<br>[1 193, 1 419]	1.60<br>[1.56, 1.64]	- 15 352<br>[- 17 778, - 12 926]
PCG antipsychotic	2.92<br>[2.78, 3.06]	9 387<br>[8 764, 10 009]	2.50<br>[2.39, 2.62]	1 256<br>[1 042, 1 470]	5.98<br>[5.8, 6.17]	- 34 176<br>[- 37 082, - 31 269]
PCG respiratory	1.67<br>[1.64, 1.7]	2 062<br>[1 692, 2 433]	1.66<br>[1.63, 1.69]	1 180<br>[1 053, 1 307]	1.09<br>[1.06, 1.12]	5 583<br>[2 507, 8 660]
PCG rheumatic conditions	1.64<br>[1.61, 1.66]	- 648<br>[- 956, - 341]	1.64<br>[1.62, 1.67]	26<br>[- 79, 131]	0.85<br>[0.82, 0.87]	7 306<br>[4 712, 9 900]
PCG thyroid disorders	1.47<br>[1.43, 1.51]	72<br>[- 442, 585]	1.47<br>[1.43, 1.51]	495<br>[319, 671]	0.90<br>[0.87, 0.94]	2 701<br>[- 1 520, 6 922]
PCG other rare diseases	2.26<br>[2.17, 2.35]	4 566<br>[3 979, 5 152]	2.22<br>[2.14, 2.3]	2 874<br>[2 673, 3 075]	1.54<br>[1.49, 1.6]	- 5 617<br>[- 9 245, - 1 989]
Urbanisation: agglomeration	0.97<br>[0.96, 0.99]	- 213<br>[- 511, 85]	0.98<br>[0.97, 0.99]	- 279<br>[- 381, - 177]	0.98<br>[0.96, 1]	1 560<br>[- 927, 4 046]
Urbanisation: rural	0.91<br>[0.9, 0.92]	- 416<br>[- 803, - 29]	0.91<br>[0.9, 0.92]	- 501<br>[- 634, - 369]	1.13<br>[1.09, 1.16]	- 6 086<br>[- 9 376, - 2 796]
Greater Region: Lake Geneva	1.2<br>[1.17, 1.23]	3 425<br>[2 832, 4 018]	1.19<br>[1.16, 1.22]	2 319<br>[2 116, 2 522]	1.01<br>[0.97, 1.06]	15 877<br>[11 270, 20 483]
	1.07	- 899	1.06	- 583	0.82	7 964

For peer review only

Greater Region: Espace Mittelland	[1.05, 1.09]	[- 1 353, - 445]	[1.04, 1.08]	[- 739, - 428]	[0.79, 0.85]	[4 098, 11 830]
Greater Region: Northwestern Switzerland	1.1<br>[1.09, 1.12]	493<br>[85, 901]	1.10<br>[1.09, 1.12]	- 255<br>[- 395, - 115]	0.95<br>[0.92, 0.98]	12 598<br>[9 256, 15 939]
Greater Region: Eastern Switzerland	0.93<br>[0.91, 0.96]	- 330<br>[- 979, 319]	0.94<br>[0.91, 0.96]	- 857<br>[- 1 079, - 634]	0.97<br>[0.92, 1.02]	1 036<br>[- 4 494, 6 567]
Greater Region: Southern Switzerland	1.26<br>[1.24, 1.29]	- 803<br>[- 1 308, - 298]	1.26<br>[1.24, 1.29]	171<br>[- 2, 344]	0.67<br>[0.64, 0.7]	13 761<br>[9 162, 18 360]
Greater Region: Central Switzerland	0.94<br>[0.91, 0.96]	- 781<br>[- 1 460, - 101]	0.93<br>[0.91, 0.95]	- 167<br>[- 399, 66]	0.91<br>[0.86, 0.96]	- 1 150<br>[- 7 175, 4 875]
Observations	798 940	545 385	798 940	543 131	798 940	47 028

Notes: The brackets show 95% confidence intervals. Abbreviations: AF: atrial fibrillation, OLS: ordinary least square, PCG: pharmaceutical cost groups.

For peer review only

Table S6. Comparison of cohort characteristics before and after propensity score matching.

	Before propensity score matching (1:1)				*After propensity score matching (1:1)*			
	**Swiss-AF**	**Controls**			**Swiss-AF**	**Controls**		
N	1 024	16 556	p	SMD	958	958	p	SMD
**Characteristics**								
Age *mean (SD)*	73.04 (8.17)	72.64 (8.52)	0.139	0.049	73.01 (8.20)	72.96 (8.37)	0.908	0.005
Sex: Male *N (%)*	741 (72.4)	11766 (71.1)	0.394	0.029	694 (72.4)	652 (68.1)	0.04	0.096
**Comorbidities (PCG)** *N (%)*								
Acid related	397 (38.8)	2802 (17.4)	<0.001	0.491	372 (38.8)	387 (40.4)	0.513	0.032
Bone	44 (4.3)	644 (4.0)	0.719	0.014	43 (4.5)	42 (4.4)	1	0.005
Cancer	35 (3.4)	510 (3.2)	0.748	0.013	33 (3.4)	29 (3.0)	0.699	0.024
Cardiovascular	754 (73.8)	10381 (63.7)	<0.001	0.22	706 (73.7)	676 (70.6)	0.14	0.07
Dementia	27 (2.6)	797 (5.0)	0.001	0.122	27 (2.8)	28 (2.9)	1	0.006
Diabetes	122 (11.9)	2298 (14.3)	0.04	0.07	110 (11.5)	101 (10.5)	0.559	0.03
Epilepsy	66 (6.5)	982 (6.1)	0.719	0.014	64 (6.7)	67 (7.0)	0.856	0.012
Glaucoma	103 (10.1)	1634 (10.2)	0.939	0.004	98 (10.2)	115 (12.0)	0.245	0.056
Gout	96 (9.4)	935 (5.8)	<0.001	0.134	89 (9.3)	87 (9.1)	0.937	0.007
Hyperlipidemia	425 (41.6)	5649 (35.0)	<0.001	0.136	395 (41.2)	371 (38.7)	0.283	0.051
Iron deficiency	66 (6.5)	567 (3.5)	<0.001	0.134	60 (6.3)	62 (6.5)	0.925	0.009
Pain	386 (37.8)	2484 (15.4)	<0.001	0.523	363 (37.9)	358 (37.4)	0.85	0.011
Psychiatric	266 (26.0)	2837 (17.6)	<0.001	0.204	250 (26.1)	269 (28.1)	0.355	0.045
Antipsychotic	16 (1.6)	878 (5.5)	<0.001	0.213	16 (1.7)	15 (1.6)	1	0.008
Respiratory	144 (14.1)	1915 (11.9)	0.045	0.064	137 (14.3)	148 (15.4)	0.521	0.032
Rheumatic	406 (39.7)	3074 (19.1)	<0.001	0.465	378 (39.5)	378 (39.5)	1	<0.001
Thyroid	87 (8.5)	908 (5.7)	<0.001	0.111	78 (8.1)	88 (9.2)	0.465	0.037
Other rare diseases	27 (2.6)	696 (4.4)	0.011	0.093	27 (2.8)	20 (2.1)	0.376	0.047
**Socioeconomic**								
Mother tongue *N (%)*			0.001	0.116			0.253	0.076
German	755 (73.7)	12944 (78.2)			737 (76.9)	759 (79.2)		
French	141 (13.8)	1708 (10.3)			132 (13.8)	108 (11.3)		

For peer review only

Italian	128 (12.5)	1904 (11.5)			89 (9.3)	91 (9.5)		
Urbanisation *N (%)*			0.236	0.056			0.973	0.011
Urban	253 (26.2)	4330 (26.2)			252 (26.3)	250 (26.1)		
Agglomeration	500 (51.9)	8953 (54.1)			497 (51.9)	502 (52.4)		
Rural	211 (21.9)	3273 (19.8)			209 (21.8)	206 (21.5)		
**Greater Region *N (%)***			<0.001	0.167			0.994	0.038
Zurich	125 (12.2)	2083 (12.6)			120 (12.5)	128 (13.4)		
Lake Geneva Region	56 (5.5)	1086 (6.6)			53 (5.5)	53 (5.5)		
Espace Mitelland	289 (28.2)	3702 (22.4)			278 (29.0)	266 (27.8)		
Northwestern Switzerland	310 (30.3)	5990 (36.2)			307 (32.0)	308 (32.2)		
Eastern Switzerland	67 (6.5)	944 (5.7)			66 (6.9)	66 (6.9)		
Southern Switzerland	125 (12.2)	1904 (11.5)			86 (9.0)	91 (9.5)		
Central Switzerland	52 (5.1)	847 (5.1)			48 (5.0)	46 (4.8)		

Notes: Abbreviations: AF: atrial fibrillation, PCG: pharmaceutical cost groups, SD: standard deviation, SMD: standardized mean difference.

For peer review only

Table S7. Regression results from ordinary (single-part) OLS modelling.

	Total costs	Outpatient costs	Inpatient costs
Cohort: Swiss-AF	5 124<br>[4 726, 5 522]	1 125<br>[986, 1 263]	3 999<br>[3 636, 4 362]
Month	8<br>[3, 14]	20<br>[18, 22]	- 12<br>[- 17, - 7]
Age	201<br>[190, 212]	44<br>[40, 47]	158<br>[147, 168]
Sex: Male	2 197<br>[1 996, 2 398]	1 158<br>[1 088, 1 228]	1 039<br>[856, 1 223]
PCG acid related disorders	3 206<br>[2 953, 3 458]	1 780<br>[1 692, 1 868]	1 426<br>[1 195, 1 656]
PCG bone diseases	6 983<br>[6 502, 7 465]	5 330<br>[5 162, 5 497]	1 653<br>[1 214, 2 093]
PCG cancer	16 504<br>[15 944, 1 7063]	12 765<br>[12 570, 12 960]	3 738<br>[3 228, 4 249]
PCG cardio	1 379<br>[1 171, 1 587]	1 118<br>[1 045, 1 190]	261<br>[71, 451]
PCG dementia	2 907<br>[2 472, 3 342]	1 320<br>[1 168, 1 471]	1 587<br>[1 190, 1 984]
PCG diabetes	4 195<br>[3 930, 4 460]	2 599<br>[2 507, 2 691]	1 596<br>[1 354, 1 838]
PCG epilepsy	7 533<br>[7 127, 7 938]	3 836<br>[3 694, 3 977]	3 697<br>[3 327, 4 067]
PCG glaucoma	434<br>[143, 725]	932<br>[830, 1 033]	- 497<br>[- 763, - 232]
PCG gout	2 168	1 061	1 107

For peer review only - http://bmjopen.bmj.com/site/about/guidelines.xhtml

	[1 784, 2 553]	[927, 1 195]	[756, 1 458]
PCG hyperlipidemia	- 396<br>[- 598, - 194]	- 40<br>[- 110, 30]	- 356<br>[- 540, - 171]
PCG iron deficiency	6 671<br>[6 173, 7 170]	4 392<br>[4 219, 4 566]	2 279<br>[1 824, 2 734]
PCG pain	6 620<br>[6 341, 6 899]	2 821<br>[2 724, 2 919]	3 799<br>[3 544, 4 054]
PCG psychiatric	3 328<br>[3 072, 3 584]	1 907<br>[1 818, 1 996]	1 421<br>[1 188, 1 654]
PCG antipsychotic	10 213<br>[9 717, 10 709]	1 960<br>[1 787, 2 132]	8 254<br>[7 800, 8 707]
PCG respiratory	2 669<br>[2 390, 2 949]	1 520<br>[1 423, 1 617]	1 149<br>[894, 1 404]
PCG rheumatic conditions	328<br>[96, 561]	528<br>[447, 609]	- 200<br>[- 412, 12]
PCG thyroid disorders	656<br>[268, 1 044]	724<br>[589, 859]	- 68<br>[- 422, 286]
PCG other rare diseases	5 353<br>[4 894, 5 812]	3 268<br>[3 108, 3 428]	2 084<br>[1 665, 2 503]
Urbanisation: agglomeration	- 205<br>[- 412, 2]	- 217<br>[- 289, - 145]	12<br>[- 177, 202]
Urbanisation: rural	-503<br>[-768, -237]	-455<br>[-548, -363]	-48<br>[-290, 195]
Greater Region: Lake Geneva	3 002<br>[2 585, 3 419]	1 963<br>[1 818, 2 108]	1 038<br>[658, 1 419]
Greater Region: Espace Mittelland	- 406<br>[- 716, - 97]	- 267<br>[- 375, - 160]	- 139<br>[- 421, 144]
	592	- 29	620

For peer review only

Greater Region: Northwestern Switzerland	[313, 870]	[- 126, 68]	[366, 875]
Greater Region: Eastern Switzerland	- 394<br>[- 826, 39]	- 623<br>[- 773, - 472]	229<br>[-166, 624]
Greater Region: Southern Switzerland	9<br>[- 342, 360]	475<br>[353, 598]	- 466<br>[- 787, - 146]
Greater Region: Central Switzerland	- 610<br>[- 1 059, - 162]	- 197<br>[- 354, - 41]	- 413<br>[- 823, - 3]
Observations	798 940	798 940	798 940

Notes: The brackets show 95% confidence intervals. Abbreviations: AF: atrial fibrillation, OLS: ordinary least square, PCG: pharmaceutical cost groups.

For peer review only

Figure S3. Cost of illness by sex and age group: estimated total AF costs per year, in Switzerland and per inhabitant in the general population.

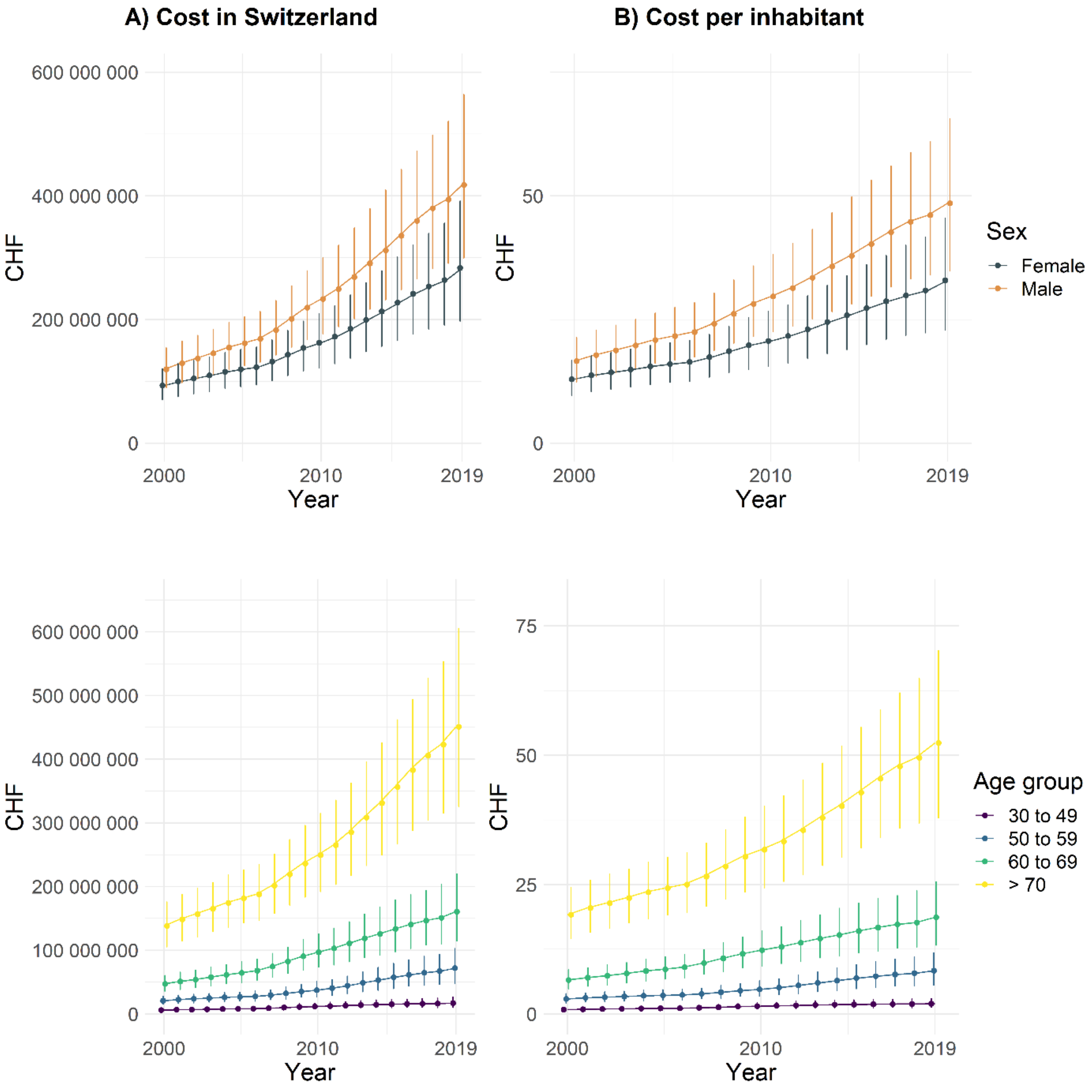

Swiss-AF investigators

University Hospital Basel and Basel University: Stefanie Aeschbacher, Katalin Bhend, Steffen Blum, Leo Bonati, David Conen, Ceylan Eken, Urs Fischer, Corinne Girroy, Elisa Hennings, Elena Herber, Vasco Iten, Philipp Krisai, Michael Kühne, Maurin Lampart, Mirko Lischer, Nina Mäder, Christine Meyer-Zürn, Pascal Meyre, Andreas U. Monsch, Luke Mosher, Christian Müller, Stefan Osswald, Rebecca E. Paladini, Anne Springer, Christian Sticherling, Thomas Szucs, Gian Völlmin.
Principal Investigator: Stefan Osswald; Local Principal Investigator: Michael Kühne

University Hospital Bern: Faculty: Faculty: Drahomir Aujesky, Juerg Fuhrer, Laurent Roten, Simon Jung, Heinrich Mattle; Research fellows: Seraina Netzer, Luise Adam, Carole Elodie Aubert, Martin Feller, Axel Loewe, Elisavet Moutzouri, Claudio Schneider; Study nurses: Tanja Flückiger, Cindy Groen, Lukas Ehrsam, Sven Hellrigl, Alexandra Nuoffer, Damiana Rakovic, Nathalie Schwab, Rylana Wenger, Tu Hanh Zarrabi Saffari. Local Principal Investigator: Nicolas Rodondi, Tobias Reichlin

Stadtspital Triemli Zurich: Christopher Beynon, Roger Dillier, Michèle Deubelbeiss, Franz Eberli, Christine Franzini, Isabel Juchli, Claudia Liedtke, Samira Murugiah, Jacqueline Nadler, Thayze Obst, Jasmin Roth, Fiona Schlomowitsch, Xiaoye Schneider, Katrin Studerus, Noreen Tynan, Dominik Weishaupt. Local Principal Investigator: Andreas Müller

Kantonspital Baden: Simone Fontana, Corinne Friedli, Silke Kuest, Karin Scheuch, Denise Hischier, Nicole Bonetti, Alexandra Grau, Jonas Villinger, Eva Laube, Philipp Baumgartner, Mark Filipovic, Marcel Frick, Giulia Montrasio, Stefanie Leuenberger, Franziska Rutz. Local Principal Investigator: Jürg-Hans Beer

Cardiocentro Lugano: Angelo Auricchio, Adriana Anesini, Cristina Camporini, Maria Luce Caputo, Francois Regoli, Martina Ronchi. Local Principal Investigator: Giulio Conte

Kantonsspital St. Gallen: Roman Brenner, David Altmann, Michaela Gemperle. Local Principal Investigator: Peter Ammann

Hôpital Cantonal Fribourg: Mathieu Firmann, Sandrine Foucras, Martine Rime. Local Principal Investigator: Daniel Hayoz

Luzerner Kantonsspital: Benjamin Berte, Kathrin Bühler, Virgina Justi, Frauke Kellner-Weldon, Melanie Koch, Brigitta Mehmann, Sonja Meier, Myriam Roth, Andrea Ruckli-Kaeppeli, Ian Russi, Kai Schmidt, Mabelle Young, Melanie Zbinden. Local Principal Investigator: Richard Kobza

Ente Ospedaliero Cantonale Lugano: Elia Rigamonti, Carlo Cereda, Alessandro Cianfoni, Maria Luisa De Perna, Jane Frangi-Kultalahti, Patrizia Assunta Mayer Melchiorre, Anica Pin,Tatiana Terrot, Luisa Vicari. Local Principal Investigator: Giorgio Moschovitis.

University Hospital Geneva: Georg Ehret, Hervé Gallet, Elise Guillermet, Francois Lazeyras, Karl-Olof Lovblad, Patrick Perret, Philippe Tavel, Cheryl Teres. Local Principal Investigator: Dipen Shah

University Hospital Lausanne: Nathalie Lauriers, Marie Méan, Sandrine Salzmann, Jürg Schläpfer. Local Principal Investigator: Alessandra Pia Porretta

Bürgerspital Solothurn: Andrea Grêt, Jan Novak, Sandra Vitelli. Local Principal Investigator: Frank-Peter Stephan

Ente Ospedaliero Cantonale Bellinzona: Jane Frangi-Kultalahti, Augusto Gallino, Luisa Vicari. Local Principal Investigator: Marcello Di Valentino

For peer review only

University of Zurich/University Hospital Zurich: Helena Aebersold, Fabienne Foster, Matthias Schwenkglenks.

Medical Image Analysis Center AG Basel: Jens Würfel (Head), Anna Altermatt, Michael Amann, Marco Düring, Petra Huber, Esther Ruberte, Tim Sinnecker, Vanessa Zuber.

Clinical Trial Unit Basel: Michael Coslovsky (Head), Pascal Benkert, Gilles Dutilh, Milica Markovic, Pia Neuschwander, Patrick Simon, Olivia Wunderlin

Schiller AG Baar: Ramun Schmid

For peer review only

STROBE Statement—Checklist of items that should be included in reports of ***cohort studies***

Estimating the cost impact of atrial fibrillation using a prospective cohort study and population-based controls

	Item No	Recommendation	addressed in the manuscript
**Title and abstract**	1	(*a*) Indicate the study's design with a commonly used term in the title or the abstract	p. 1
		(*b*) Provide in the abstract an informative and balanced summary of what was done and what was found	p. 3
**Introduction**			
Background/rationale	2	Explain the scientific background and rationale for the investigation being reported	p. 5
Objectives	3	State specific objectives, including any prespecified hypotheses	p. 5
**Methods**			
Study design	4	Present key elements of study design early in the paper	p. 6
Setting	5	Describe the setting, locations, and relevant dates, including periods of recruitment, exposure, follow-up, and data collection	p. 6
Participants	6	(*a*) Give the eligibility criteria, and the sources and methods of selection of participants. Describe methods of follow-up	p. 6, 7
		(*b*) For matched studies, give matching criteria and number of exposed and unexposed	NA
Variables	7	Clearly define all outcomes, exposures, predictors, potential confounders, and effect modifiers. Give diagnostic criteria, if applicable	p. 7
Data sources/ measurement	8*	For each variable of interest, give sources of data and details of methods of assessment (measurement). Describe comparability of assessment methods if there is more than one group	p. 6-8
Bias	9	Describe any efforts to address potential sources of bias	p. 6-8
Study size	10	Explain how the study size was arrived at	p. 6-7, Table S1-S2
Quantitative variables	11	Explain how quantitative variables were handled in the analyses. If applicable, describe which groupings were chosen and why	p. 7-9
Statistical methods	12	(*a*) Describe all statistical methods, including those used to control for confounding	p. 7-9
		(*b*) Describe any methods used to examine subgroups and interactions	p. 7-9
		(*c*) Explain how missing data were addressed	p. 7
		(*d*) If applicable, explain how loss to follow-up was addressed	p. 7
		(*e*) Describe any sensitivity analyses	p. 7-8
**Results**			
Participants	13*	(a) Report numbers of individuals at each stage of study—eg numbers potentially eligible, examined for eligibility, confirmed eligible, included in the study, completing follow-up, and analysed	p. 10, Table S1-S2
		(b) Give reasons for non-participation at each stage	p. 10
		(c) Consider use of a flow diagram	p. 10, Figure 1
Descriptive data	14*	(a) Give characteristics of study participants (eg demographic, clinical, social) and information on exposures and potential confounders	p.10, Table 1, Figure S1
		(b) Indicate number of participants with missing data for each variable of	NA

For peer review only - http://bmjopen.bmj.com/site/about/guidelines.xhtml

		interest	
		(c) Summarise follow-up time (eg, average and total amount)	Figure S1
Outcome data	15*	Report numbers of outcome events or summary measures over time	p. 10
Main results	16	(*a*) Give unadjusted estimates and, if applicable, confounder-adjusted estimates and their precision (eg, 95% confidence interval). Make clear which confounders were adjusted for and why they were included	p. 10, Tables S4-S7
		(*b*) Report category boundaries when continuous variables were categorized	NA
		(*c*) If relevant, consider translating estimates of relative risk into absolute risk for a meaningful time period	NA
Other analyses	17	Report other analyses done—eg analyses of subgroups and interactions, and sensitivity analyses	p. 10-11; Figures S3
**Discussion**			
Key results	18	Summarise key results with reference to study objectives	p. 11-13
Limitations	19	Discuss limitations of the study, taking into account sources of potential bias or imprecision. Discuss both direction and magnitude of any potential bias	p. 12-13
Interpretation	20	Give a cautious overall interpretation of results considering objectives, limitations, multiplicity of analyses, results from similar studies, and other relevant evidence	p. 11-13
Generalisability	21	Discuss the generalisability (external validity) of the study results	p. 11-13
**Other information**			
Funding	22	Give the source of funding and the role of the funders for the present study and, if applicable, for the original study on which the present article is based	p. 14

*Give information separately for exposed and unexposed groups.

Note: An Explanation and Elaboration article discusses each checklist item and gives methodological background and published examples of transparent reporting. The STROBE checklist is best used in conjunction with this article (freely available on the Web sites of PLoS Medicine at http://www.plosmedicine.org/, Annals of Internal Medicine at http://www.annals.org/, and Epidemiology at http://www.epidem.com/). Information on the STROBE Initiative is available at http://www.strobe-statement.org.

For peer review only

BMJ Open

Estimating the cost impact of atrial fibrillation using a prospective cohort study and population-based controls

Journal:	*BMJ Open*
Manuscript ID	bmjopen-2023-072080.R1
Article Type:	Original research
Date Submitted by the Author:	13-Jul-2023

Complete List of Authors:	Aebersold, Helena; University of Zurich, Epidemiology, Biostatistics and Prevention Institute<br>Foster-Witassek , Fabienne ; University of Zurich, Epidemiology, Biostatistics and Prevention Institute<br>Serra-Burriel, Miquel; University of Zurich, Epidemiology, Biostatistics and Prevention Institute<br>Brüngger, Beat; Helsana Versicherungen AG, Department of Health Sciences<br>Aeschbacher, Stefanie; University Hospital Basel, Department of Medicine, Cardiology Division; University Hospital Basel, Cardiovascular Research Institute Basel<br>Beer, Jürg-Hans; Baden Cantonal Hospital, Department of Medicine, Cantonal Hospital of Baden; University of Zurich, Center for Molecular Cardiology<br>Blozik, Eva; University of Zurich, Institute of Primary Care<br>blum, manuel; University of Bern Institute of Primary Health Care; Inselspital University Hospital Bern, Department of General Internal Medicine<br>Bonati, Leo; University of Basel, Department of Neurology; Reha Rheinfelden, Research Department<br>Conen, David; McMaster University, Population Health Research Institute<br>Conte, Giulio; Cardiocentro Ticino, Division of Cardiology<br>Felder, Stefan; University of Basel, Faculty of Business and Economics<br>Huber, Carola; Helsana Versicherungen AG, Department of Health Sciences<br>Kuehne, Michael; University of Basel, Department of Medicine, Cardiology Division; University Hospital Basel, Cardiovascular Research Institute Basel<br>Moschovitis, Giorgio; Ospedale Regionale di Lugano-Civico e Italiano, Division of Cardiology<br>Mueller, Andreas; Municipal Hospital Triemli, Department of Cardiology<br>Paladini, Rebecca E; University Hospital Basel, Department of Medicine, Cardiology Division; University Hospital Basel, Cardiovascular Research Institute Basel<br>Reichlin, Tobias; Inselspital University Hospital Bern, Cardiology<br>Rodondi, Nicolas; University of Bern Institute of Primary Health Care; Inselspital University Hospital Bern, Department of General Internal Medicine<br>Springer, Anne; University Hospital Basel, Department of Medicine, Cardiology Division; University Hospital Basel, Cardiovascular Research Institute Basel

	Stauber, Annina; Municipal Hospital Triemli, Department of Cardiology<br>Sticherling, Christian; University Hospital Basel, Department of Medicine, Cardiology Division; University Hospital Basel, Cardiovascular Research Institute Basel<br>Szucs, Thomas; University of Basel, Institute of Pharmaceutical Medicine<br>Osswald , Stefan; University Hospital Basel, Department of Medicine, Cardiology Division; University Hospital Basel, Cardiovascular Research Institute Basel<br>Schwenkglenks, M; University of Zurich, Epidemiology, Biostatistics and Prevention Institute; University of Basel, Institute of Pharmaceutical Medicine
**Primary Subject Heading**:	Health economics
Secondary Subject Heading:	Epidemiology, Cardiovascular medicine
Keywords:	HEALTH ECONOMICS, CARDIOLOGY, Cardiac Epidemiology < CARDIOLOGY

SCHOLARONE™ Manuscripts

I, the Submitting Author has the right to grant and does grant on behalf of all authors of the Work (as defined in the below author licence), an exclusive licence and/or a non-exclusive licence for contributions from authors who are: i) UK Crown employees; ii) where BMJ has agreed a CC-BY licence shall apply, and/or iii) in accordance with the terms applicable for US Federal Government officers or employees acting as part of their official duties; on a worldwide, perpetual, irrevocable, royalty-free basis to BMJ Publishing Group Ltd ("BMJ") its licensees and where the relevant Journal is co-owned by BMJ to the co-owners of the Journal, to publish the Work in this journal and any other BMJ products and to exploit all rights, as set out in our licence.

The Submitting Author accepts and understands that any supply made under these terms is made by BMJ to the Submitting Author unless you are acting as an employee on behalf of your employer or a postgraduate student of an affiliated institution which is paying any applicable article publishing charge ("APC") for Open Access articles. Where the Submitting Author wishes to make the Work available on an Open Access basis (and intends to pay the relevant APC), the terms of reuse of such Open Access shall be governed by a Creative Commons licence – details of these licences and which Creative Commons licence will apply to this Work are set out in our licence referred to above.

Other than as permitted in any relevant BMJ Author's Self Archiving Policies, I confirm this Work has not been accepted for publication elsewhere, is not being considered for publication elsewhere and does not duplicate material already published. I confirm all authors consent to publication of this Work and authorise the granting of this licence.

For peer review only

Estimating the cost impact of atrial fibrillation using a prospective cohort study and population-based controls

Authors

Aebersold Helena[a], Foster-Witassek Fabienne[a], Serra-Burriel Miquel[a], Beat Brüngger[b], Aeschbacher Stefanie[c,d], Beer Jürg H.[e,f], Blozik Eva[g], Blum Manuel R.[h,q], Bonati Leo H.[i,j], Conen David[k], Conte Giulio[l], Felder Stefan[m], Huber Carola A.[b], Kuehne Michael[c,d], Moschovitis Giorgio[n], Mueller Andreas[o], Paladini Rebecca E.[c,d], Reichlin Tobias[p], Rodondi Nicolas[h,q], Springer Anne[c,d], Stauber Annina[o], Sticherling Christian[c,d], Szucs Thomas[r], Osswald Stefan[c,d], Schwenkglenks Matthias[a,r], on behalf of the Swiss-AF Investigators

[a] Epidemiology, Biostatistics and Prevention Institute, University of Zurich, Zurich, Switzerland

[b] Department of Health Sciences, Helsana Group, Zurich, Switzerland

[c] Cardiology Division, Department of Medicine, University Hospital Basel, Switzerland

[d] Cardiovascular Research Institute Basel, University Hospital Basel, Switzerland

[e] Department of Medicine, Cantonal Hospital of Baden, Switzerland

[f] Center for Molecular Cardiology, University of Zürich, Switzerland

[g] Institute of Primary Care, University of Zurich, Zurich, Switzerland

[h] Institute of Primary Health Care (BIHAM), University of Bern, Switzerland

[i] Department of Neurology, University Hospital Basel, Basel Switzerland

[j] Research Department, Reha Rheinfelden, Rheinfelden, Switzerland

[k] Population Health Research Institute, McMaster University, Hamilton, Canada

[l] Division of Cardiology, Cardiocentro Ticino (CCT), Lugano, Switzerland

[m] University of Basel, Faculty of Business and Economics, Switzerland

[n] Division of Cardiology, Ente Ospedaliero Cantonale (EOC), Istituto Cardiocentro Ticino, Ospedale Regionale di Lugano, Lugano, Switzerland

[o] Department of Cardiology, Triemli Hospital Zurich, Switzerland

[p] Department of Cardiology, Inselspital, Bern University Hospital, University of Bern, Switzerland

[q] Department of General Internal Medicine, Inselspital, Bern University Hospital, University of Bern, Switzerland

[r] Institute of Pharmaceutical Medicine (ECPM), University of Basel, Basel, Switzerland

Correspondence

Helena Aebersold

For peer review only - http://bmjopen.bmj.com/site/about/guidelines.xhtml

University of Zurich

Epidemiology, Biostatistics & Prevention Institute (EBPI)

Hirschengraben 84, 8001 Zurich, Switzerland

helena.aebersold@uzh.ch

Word Count: 3,093

For peer review only

For peer review only - http://bmjopen.bmj.com/site/about/guidelines.xhtml

Abstract

Aims

Atrial Fibrillation (AF) costs are expected to be substantial, but cost comparisons with the general population are scarce. Using data from the prospective Swiss-AF cohort study and population-based controls, we estimated the impact of AF on direct healthcare costs from the Swiss statutory health insurance perspective.

Methods

Swiss-AF patients, enrolled from 2014-2017, had documented, prevalent AF. We analysed 5 years of follow-up, where clinical data, and health insurance claims in 42% of the patients were collected on a yearly basis. Controls from a health insurance claims database were matched for demographics and region. The cost impact of AF was estimated using five different methods: i) ordinary least square regression (OLS), ii) OLS-based two-part modelling, iii) generalised linear model (GLM)-based two-part modelling, iv) 1:1 nearest neighbour propensity score matching, and v) a cost adjudication algorithm using Swiss-AF data non-comparatively and considering clinical data. Cost-of-illness at the Swiss national level was modelled using obtained cost estimates, prevalence from the Global Burden of Disease Project, and Swiss population data.

Results

The 1,024 Swiss-AF patients with available claims data were compared with 16,556 controls without known AF. AF patients accrued CHF 5,600 (EUR 5,091) of AF-related direct healthcare costs per year, in addition to non-AF related healthcare costs of CHF 11,100 (EUR 10,091) per year accrued by AF patients and controls. All five methods yielded comparable results. AF-related costs at the national level were estimated to amount to 1% of Swiss healthcare expenditure.

Conclusions

We robustly found direct medical costs of AF patients were 50% higher than those of population-based controls. Such information on the incremental cost burden of AF may support healthcare capacity planning.

Keywords

atrial fibrillation, cost-of-illness, two-part model, population-based controls, healthcare costs

For peer review only - http://bmjopen.bmj.com/site/about/guidelines.xhtml

For peer review only

For peer review only - http://bmjopen.bmj.com/site/about/guidelines.xhtml

Strengths and limitations

- This study used 5 years of follow-up data from a large prospective cohort of prevalent atrial fibrillation (AF) patients.
- The direct medical cost impact of AF was assessed by comparison with population-based controls drawn from a large health insurance database.
- Several regression-based and propensity score-based methods were used to judge robustness and AF costs were also assessed using a non-comparative approach.
- The cohort of AF patients may not be fully representative of all AF patients.
- A limited degree of residual presence of AF in the control population cannot be ruled out.

For peer review only

Introduction

Atrial fibrillation (AF) is the most common form of serious arrhythmia worldwide, and a major cause of stroke and heart failure. More than 11 million people live with AF in Europe.[1,2] Given demographic ageing, Europe is expected to face a larger increase in AF prevalence by 2050 than any other region globally.[1]

Several studies on cost-of-illness of AF have estimated costs at the patient or nationwide levels. Direct healthcare costs per patient were estimated to range from EUR 2,315–3,307 annually in Europe[3–6], and from USD 6,410–8,705 in the USA[7,8]. At the national level, direct costs of AF in Europe may range from EUR 660–2,548 million[9–12], in the US they were estimated at around USD 6 billion[8,13]. These costs are substantial, accounting for 0.28-1.7% of the national health expenditures of these countries[12,14–16].

So far, most attempts assessing the cost impact of AF remained descriptive. To our knowledge, only two studies[8,16] compared costs between AF patients and a control population. Even less evidence is available for cost changes since 2010, as most cost-of-illness studies rely on data collected earlier.

We used a recent real-world dataset from a large prospective cohort study of AF patients to assess the yearly cost impact of AF. Comparing with a population-based control sample, direct healthcare costs of AF were estimated at the patient level and transferred to the national level. Results were compared with estimates resulting from an adjudication algorithm only using the cohort data in a non-comparative approach.

For peer review only

For peer review only - http://bmjopen.bmj.com/site/about/guidelines.xhtml

Methods

Study Design and Data Sources

Swiss-AF is a large, ongoing prospective observational cohort study across 14 clinical centres in Switzerland, investigating AF-related cognition, complications, and economic aspects. Patients were enrolled between 2014 and 2017 if they had a history of documented AF and were older than 65 years; 228 patients were enrolled aged 45-64 to enhance the study of socio-economic aspects. A data cut of 2014-2020 was used in this analysis. The detailed study setup has been published earlier.[17]

Alongside clinical data, health economic data were collected. These included medical resource use at the study centres, and health insurance claims from four cooperating health insurers covering 42% of the study sample. In Switzerland, health insurance is compulsory and offered to anyone, covering inpatient and outpatient services. The benefit package is uniform across the country and defined by law.

To assess the cost impact of AF, a population-based reference sample was provided by Helsana, an insurer covering about 15% of the Swiss population. Helsana enrolees were eligible for the reference sample if they were not Swiss-AF patients, were in the same age range as the Swiss-AF population, and had statutory health insurance claims data available for a period equivalent to the one available for Swiss-AF patients. For the reference sample a subset of 19,002 patients was randomly selected, frequency-matched to the Swiss-AF patients by age, gender and geographic region (supplementary **Table S1**). To ensure similar observation times, start dates for the controls were randomly assigned using the distribution of Swiss-AF enrolment dates. Sensitivity analyses with different starting and ending dates were run without altering the results significantly. Individuals within the reference sample could have AF, as Swiss claims data do not have direct diagnosis information for outpatient services. Hence, a categorization algorithm (supplementary **Table S2**) was developed together with clinicians from the Swiss-AF centres to distinguish such persons. Using codes from the International Classification of Diseases 10th Revision (ICD10)[18], the Swiss diagnosis related group-based (SwissDRG) [19] flat fee reimbursement system for inpatient episodes, the Swiss invasive medical procedures catalogue (CHOP)[20], the anatomical therapeutic chemical classification (ATC) of medicines[21], and the national tariff for outpatient physician services (Tarmed)[22], three categories resulted: “AF likely”, “AF possible”, and “AF not obvious”. We assigned the category of “AF likely” to patients with a very high probability of having AF, as most codes were hospitalisation-based. Persons categorized as “AF possible” had codes possibly but not clearly allocable to AF. All other patients were classified as “AF not obvious” and considered as controls (**Figure 1**).

For peer review only - http://bmjopen.bmj.com/site/about/guidelines.xhtml

Equivalent claims data were available for the Swiss-AF and control patients, reflecting all claims for reimbursement by the Swiss statutory health insurance. The claims data included detailed information on outpatient services and drugs, and less detailed information on inpatient services based on SwissDRG[19]. Given the absence of clinical data for the control sample, the presence of major chronic morbidities was approximated, uniformly for Swiss-AF patients and controls, based on outpatient drug claims, using the pharmaceutical cost groups (PCG) approach[23].

Outcome Measures

Our main outcome of interest was the AF-induced part of direct medical healthcare costs from the perspective of the Swiss statutory health insurance. To assess the cost impact of AF, the Swiss-AF patients were compared with the population-based controls, using different multivariable regression methods: i) ordinary least square regression (OLS), ii) OLS-based two-part modelling, iii) generalised linear model (GLM)-based two-part modelling, and iv) 1:1 nearest neighbour propensity score matching. Furthermore, v) estimates were compared with AF costs estimated using a previously developed adjudication algorithm[24]. In brief, the AF-adjudication algorithm combined clinical event data collected in Swiss-AF with health insurance claims, adjudicating each cost component as AF-related or non-AF related. We distinguished between total, outpatient, and inpatient costs. All cost calculations considered individual start dates and follow-up times and were aggregated to a yearly level. Given the relative stability of prices over the observation period, costs were taken as recorded in the health insurance database. To facilitate comparison with other countries, main cost results are presented in Euros (EUR) in addition to Swiss francs (CHF), based on an exchange rate (averaged 2014-2020) of EUR 1.0 = CHF 1.1. Individual follow-up times were censored at five years after the start date due to the small number of longer follow-up periods available.

Covariates

Covariates available for both the Swiss-AF and control population included the following types: Firstly, patient characteristics: age, sex, and area of residence (greater regions of Switzerland). Secondly, PCGs as proxies for comorbidities: acid related disorders (i.e. gastro-oesophageal reflux disease), bone diseases, cancer, dementia, epilepsy, respiratory illness, rheumatic conditions, glaucoma, gout, iron deficiency, chronic pain, psychiatric diseases, use of antipsychotic drugs, thyroid disease, and other rare diseases. Thirdly, year of follow-up. Insurance characteristics were obtained from three of four insurers and considered in a sensitivity analysis.

For peer review only - http://bmjopen.bmj.com/site/about/guidelines.xhtml

Statistical analysis and estimation of AF costs per person

First, the characteristics of the included Swiss-AF and control patients were described with standard methods. Healthcare costs per patient and cost trajectories over time were descriptively analysed for both populations, distinguishing between total, outpatient, and inpatient costs. Cost trajectories over time were depicted as line plots not considering missing data points.

Second, the mentioned multivariable regression approaches were pursued to assess the cost impact of AF, using the above-listed covariates as independent variables. All approaches included a time fixed effect for month of observation.

The two-part alternatives to OLS were pursued because healthcare costs are characterised by a significant proportion of zero values and right-skewed distributions of non-zero costs.[25] In the first part of the two-part models, the probability of having any costs in a given year of follow-up was estimated using a logistic model. The same covariates were used in the second part of the model, estimating the costs conditional on having occurred. Again, OLS was chosen for the second part to achieve direct cost estimates. Alternatively, generalised linear models (GLMs) with an assumed gamma distribution and logarithmic link function were used in the second part, to better account for the heteroscedasticity typically present in healthcare costs.[26] The cost ratios of the GLM part were converted to marginal effects to enable a direct comparison with the OLS-based results. Mean annual costs were finally calculated by multiplying the predicted values of both modelling parts.[27] To estimate the marginal cost impact of AF, all patients were assumed to have AF, or not to have AF. Both sets of predicted values were calculated, and the difference was interpreted as the cost impact of AF.[28] A further analysis was run by estimating the AF costs with propensity score matching, using a 1:1 nearest neighbour approach. Given the characteristics of the data, the GLM-based two-part modelling approach was considered theoretically most suitable, and the corresponding results were treated as primary.

Third, the different regression-based estimates of AF costs were compared with the estimates of AF costs resulting from applying the AF adjudication algorithm to the Swiss-AF patients' claims data.[24]

AF costs at the national level

Fourth, cost of illness of AF for Switzerland was roughly approximated as total costs per year, and costs per inhabitant and year, for the time period 2000-2019. Mean annual AF-related costs were taken from the GLM-based two-part model and assumed to follow the trend of healthcare expenditures in Switzerland for the period (index 2019

For peer review only - http://bmjopen.bmj.com/site/about/guidelines.xhtml

= 100%). AF prevalence was taken from the data base of the Global Burden of Disease Project for the Swiss population older than 30.[2] For cost calculations per capita, the Swiss population size was used with no age restriction, obtained from the Swiss Federal Statistical Office[29].

All analyses were conducted using R V3.6.3.

Patient and public involvement

Patients or the public were not involved in the design, or conduct, or reporting, or dissemination plans of our research.

For peer review only

For peer review only - http://bmjopen.bmj.com/site/about/guidelines.xhtml

Results

Patient population

Figure 1 shows the cohort selection. Of 2,415 Swiss-AF patients, 1,024 (42.4%) had claims data available and were included in the analysis (patients without available claims data showed similar characteristics[24]). In the population-based reference sample, 16,556 individuals were classified as "AF not obvious" and included as controls. Baseline characteristics by cohort are shown in **Table 1**. The supplementary **Figure S1** provides details on the numbers of patients at risk, cumulative numbers of events, the development of costs and Kaplan-Meier survival estimates across the full observation period 2014-2020 by cohort.

Table 1. Baseline characteristics.

	Swiss-AF	Controls	
N	1 024	16 556	SMD
**Characteristics**			
Age *mean (SD)*	73.04 (8.17)	72.64 (8.52)	0.401
Sex male *N (%)*	741 (72.4)	11766 (71.1)	0.145
**Comorbidities (PCG) *N (%)***			
Acid related disorders	397 (38.8)	2802 (17.4)	0.326
Bone diseases	44 (4.3)	644 (4.0)	0.035
Cancer	35 (3.4)	510 (3.2)	0.067
Cardiovascular	754 (73.8)	10381 (63.7)	0.402
Dementia	27 (2.6)	797 (5.0)	0.097
Diabetes	122 (11.9)	2298 (14.3)	0.161
Epilepsy	66 (6.5)	982 (6.1)	0.077
Glaucoma	103 (10.1)	1634 (10.2)	0.035
Gout	96 (9.4)	935 (5.8)	0.151
Hyperlipidaemia	425 (41.6)	5649 (35.0)	0.174
Iron deficiency	66 (6.5)	567 (3.5)	0.116
Pain	386 (37.8)	2484 (15.4)	0.347
Psychiatric	266 (26.0)	2837 (17.6)	0.136
Antipsychotic	16 (1.6)	878 (5.5)	0.142
Respiratory	144 (14.1)	1915 (11.9)	0.141
Rheumatic conditions	406 (39.7)	3074 (19.1)	0.309
Thyroid disorders	87 (8.5)	908 (5.7)	0.083
Other rare diseases	27 (2.6)	696 (4.4)	0.107
Number of PCGs *mean (SD)*	3.39 (2.53)	2.41 (1.98)	0.31
**Mother tongue N (%)**			0.108
German	755 (73.7)	12944 (78.2)	
French	141 (13.8)	1708 (10.3)	
Italian	128 (12.5)	1904 (11.5)	
**Greater Region *N (%)***			0.182
Zurich	125 (12.2)	2083 (12.6)	
Lake Geneva Region	56 (5.5)	1086 (6.6)	

For peer review only - http://bmjopen.bmj.com/site/about/guidelines.xhtml

Espace Mitelland	289 (28.2)	3702 (22.4)	
Northwestern Switzerland	310 (30.3)	5990 (36.2)	
Eastern Switzerland	67 (6.5)	944 (5.7)	
Southern Switzerland	125 (12.2)	1904 (11.5)	
Central Switzerland	52 (5.1)	847 (5.1)	

Abbreviations: AF: atrial fibrillation, PCG: pharmaceutical cost groups, SD: standard deviation, SMD: standardized mean difference.

Healthcare costs over time

The evolution of mean annual costs by cohort and cost component is depicted in **Figure 2** (details in **Table S3**, **Figure S2**). The unadjusted average total cost per patient and year amounted to CHF 19,037 (EUR 17,306) for Swiss-AF patients, around 1.7-fold more than for control patients. In both cohorts, inpatient and outpatient costs each contributed half of the total costs on average.

AF-related and non-AF related healthcare costs

Table 2 compares the model-based estimated differences in healthcare costs between AF patients and controls, interpreted as AF-related costs. Details for each model are in the supplement (**Tables S4-S7**). All estimates of AF-related costs were in a similar range. The GLM-based two-part model yielded total AF costs of CHF 5,588 (EUR 5,080) annually, while outpatient costs were CHF 1,425 (EUR 1,295), and inpatient costs CHF 2,779 (EUR 2,526).

Table 2. Estimates of difference in healthcare costs between AF patients and controls: comparison of alternative models.

	Dependent variable	*Model*			
		**Two part GLM**	**Two part OLS**	**Propensity score matching**	**OLS**
**Total costs**	*Odds ratio (OR) (Logistic part)*	1.50 [1.46, 1.54]		–	–
	Marginal effect / Cost estimate (GLM / OLS part)	6 374 [5 609, 7 139]	5 743 [5 210, 6 277]	–	–
	Combined two part / direct estimate	5 588	5 187	5 692	5 124

For peer review only - http://bmjopen.bmj.com/site/about/guidelines.xhtml

For peer review only

**Outpatient costs**	*OR (Logistic part)*	1.46 [1.42, 1.50]		–	–
	Marginal effect / Cost estimate (GLM / OLS part)	1 299 [1 097, 1 501]	1 043 [860, 1 226]	–	–
	Combined two part / direct estimate	1 425	1 246	1 342	1 124
**Inpatient costs**	*OR (logistic part)*	1.13 [1.08, 1.17]		–	–
	Marginal effect / Cost estimate (GLM / OLS part)	35 154 [28 827, 41 481]	37 322 [32 916, 41 728]	–	–
	Combined two part / direct estimate	2 779	2 957	4 350	3 999

Notes: The two part models used a logistic regression in the first part, and GLM or OLS respectively in the second part. Propensity score matching was done 1:1, and OLS refers to a direct (non-two part) OLS estimate. The brackets show 95% confidence intervals. An exchange rate of EUR 1.0 = CHF 1.1 can be used to convert the costs into Euros to facilitate comparison with other countries. Abbreviations: GLM: generalised linear model, OLS: ordinary least squares regression, OR: odds ratio.

For peer review only

Figure 3 compares the estimates of AF-related costs from the GLM- and OLS-based two-part models with the estimates for the Swiss-AF patients based on the AF-adjudication algorithm without controls. The estimated AF-related costs were very similar for all three methods, ranging from CHF 5,187 (OLS-based) to CHF 5,588 (GLM-based), and CHF 5,679 (adjudication-based). AF-related costs from the adjudication algorithm are shown by subgroup, revealing details not available from the regression estimates: AF-treatment costs contributed most to AF-related costs, while the costs of AF-related complications contributed relatively little. Non-AF-related costs induced by diseases other than AF, i.e. accrued by the Swiss-AF patients and the controls, were similar across all approaches. They amounted to CHF 11,100 (EUR 10,091) per year OLS- and GLM-based, and CHF 13,400 (EUR 12,182) per year adjudication-based.

Cost of illness in Switzerland

For peer review only - http://bmjopen.bmj.com/site/about/guidelines.xhtml

Figure S3 shows the estimated evolution of AF-related costs at the Swiss national level, in total and in CHF per inhabitant. Since 2000 the increase in costs was faster than the prevalence increase of AF in the population. Estimates amounted to CHF 700 million (EUR 636 million) in 2019, equivalent to about CHF 80 per inhabitant. Male patients contributed 1.5 times more to the costs than female patients due to higher prevalence, and most of the costs were accrued in patients older than 70 years (supplementary **Figure S4**).

For peer review only

For peer review only - http://bmjopen.bmj.com/site/about/guidelines.xhtml

Discussion

This study presents up-to-date evidence of real-world AF-related healthcare costs. To the best of our knowledge, it is the first study comparing AF-related cost estimates using population-based controls with a data-derived bottom-up approach to adjudication of AF costs. We obtained similar results for all estimation methods used: mean annual AF-related costs amounted to CHF 5,600 (EUR 5,091); indicating roughly 50% higher direct medical costs of Swiss AF patients compared to the population-based controls. At the national level, AF-related costs amounted to CHF 700 million (EUR 636 million) in 2019, equivalent to about 1% of the Swiss healthcare expenditure.

Our estimates of AF-related direct medical costs of CHF 5,600 annually are consistent with previously published estimates, despite notable differences in study designs and data collection approaches. In Europe, annual direct medical cost estimates at the patient level ranged from EUR 2,315–3,785 (Spain EUR 2,315 (2006)[4], Germany EUR 2,405 (2005)[3], Sweden EUR 2,787 (2006)[3], Italy EUR 3,225 (2006)[4], France EUR 3,307 (2004)[6], Scotland GBP 3,785 (2015)[5]). After accounting for purchasing power parity (PPP), our estimate for Switzerland is still somewhat higher, but comparable. As Ringborg[4] has shown, differences within Europe are notable even after accounting for PPP, reflecting differences in the healthcare systems of the countries. Moreover, Switzerland is known to have a relatively more expensive healthcare system than other European countries.

Transferred to the Swiss national level, direct medical AF costs amounted to CHF 700 million in 2019. AF-related cost estimates for European countries ranged from EUR 660–2,548 million (Germany EUR 660 million (2004)[9], France EUR 1,942 million (2012)[10], Sweden EUR 240 million (2007)[11], United Kingdom GBP 244 million (1995) to model-based estimates of 2,548 million (2020)[12,15]). In the USA, AF-related costs were estimated to be around USD 6 billion (2008)[8,13]. It is difficult to compare the existing cost-of-illness studies due to methodological differences, while differences in their timing and in population size can e.g. be captured by expressing AF-related costs as a share of the gross domestic product (GDP) or total healthcare expenditure in the relevant year. In Switzerland, the estimated AF-related costs amounted to 0.1% of the GDP in 2019, equivalent to roughly 1% of the total healthcare expenditure. This is again comparable with the existing literature. In Portugal, AF-related costs were estimated to be 0.08% of the GDP, including indirect costs but excluding bleeding-related events and services.[30] AF-related cost estimates as a share of healthcare expenditures ranged from 0.28-1.7%:

For peer review only - http://bmjopen.bmj.com/site/about/guidelines.xhtml

Germany 0.28%[14], USA 0.42%[14], UK 0.62%[12], Australia 1.01%[14], UK based on modelling 0.91-1.62%[15], Denmark 1.7%[16].

Our estimates of AF-related costs in the large, prospective Swiss-AF cohort were highly consistent and robust. In particular, the regression-based estimates of AF costs using a matched control population were remarkably similar to the cost estimates based on direct adjudication to AF. The adjudication algorithm was derived using clinical and claims data for the Swiss AF sample only, without comparison to the population-based controls. So far, most literature has focussed on estimating costs from clinical or claims data[3,4,6,9,10,30]; only very few comparisons with a control population are available[8,16]. While lending strong credibility to our results, the observed similarity also suggests that lacking controls, the AF-related portion of healthcare costs may still be estimated quite accurately with a well-defined algorithm supported by clinical data.

There are still several limitations of our work requiring discussion. Most importantly, the Swiss-AF study population is not truly representative of all AF patients in Switzerland, given enrolment in in- and outpatient clinical centres and an expected under-representation of patients younger than 65 years driven by eligibility criteria. It would in fact be extremely difficult, if not impossible, to recruit a truly representative sample of AF patients into any study. We still expect our cost estimates to provide a reasonable approximation of the typical AF-related costs of Swiss patients with clinically diagnosed AF. The decision to enrol patients independently of time since diagnosis supports this notion, all the more given the observed high degree of stability of our results over time. However, we cannot exclude that enrolment of the Swiss-AF patients in clinical centres may have led to a certain overestimation of inpatient cost in the first year of observation. Second, the selection algorithm used to define the control population is likely to have missed some patients with AF. However, the lack of exclusion of these patients should not have biased the results strongly, as they did not display indicators of AF-related hospitalization or major procedures. If anything, a moderate underestimation of AF costs may have occurred. Third, cost calculations were based on claims data, and not all claims may have been handed in for reimbursement. However, in patients with a chronic disease and substantial healthcare costs, this is rather not expected. We could not acquire insurance characteristics from one insurer and have consider these in a sensitivity analysis without distortion of our results. Fourth, the controls were provided by one health insurance only. Major differences between insurers are not expected in the Swiss statutory health insurance, as the primary benefit package is uniform across the country and defined by law. A further limitation affects the estimation of the cost-of-

For peer review only - http://bmjopen.bmj.com/site/about/guidelines.xhtml

illness at the national level. There were several assumptions made: a) AF-related cost estimates were based on the results of the GLM-based two part model, b) the development of costs per patient over time was assumed to follow the development of healthcare expenditures in Switzerland, and c) AF patients under the age of 30 were not considered in the prevalence estimates. As a last limitation, this analysis focused on direct medical costs from the perspective of the Swiss statutory health insurance. Costs of lost productivity were not considered and the total impact of AF on the economy was thus not captured. Separate work will address the topic of impact of AF on productivity in younger Swiss-AF patients.

In conclusion, the results of this study indicate that AF patients incur 50% higher costs than comparable population-based controls. Costs were at a comparable level as reported by other cost-of-illness studies for AF. Different regression-based approaches to estimating AF-related costs led to similar results, confirming the robustness of our findings. A well-defined bottom-up approach using clinical and claims data but no control population also yielded similar results. This finding is valuable for the interpretation of the existing cost-of-illness literature and may inform decisions on investments in healthcare policies. To control the high costs of AF, future steps may include conducting real-world analyses to understand contributing factors and services, assessing the cost-effectiveness of AF-related treatments to guide resource allocation, and studying risk factors to develop targeted interventions aimed at reducing AF incidence and improving healthcare efficiency.

For peer review only

Funding

This work is supported by grants of the Swiss National Science Foundation (grant numbers 105318_189195 / 1, 33CS30_148474, 33CS30_177520, 32473B_176178, and 32003B_197524), the Swiss Heart Foundation, the Foundation for Cardiovascular Research Basel (FCVR), and the University of Basel.

Acknowledgements

We thank Lukas Kauer from the health insurance CSS, Pascal Godet from the health insurance Sanitas, Andri Signorell from the health insurance Helsana, and the health insurance KPT for providing the claims data for this study.

Contributors

All authors certify that they have participated sufficiently in the work to take public responsibility for the content, including participation in the concept, design, analysis, writing, or revision of the manuscript.

Concept: HA, FF, MSB, MS
Design and methods: HA, FF, MSB, MS, BB, SA, JHB, EB, MRB, LHB, DC, GC, SF, CAH, MK, GM, AM, REP, TR, NR, ASp, ASt, CS, TS, SO
Acquisition of data: HA, FF, MSB, MS, BB, SA, ASp, MK, GM, REP, SO
Analysis: HA, FF, MSB, MS
Interpretation of results: all authors
Writing: HA, FF, MSB, MS
Reviewing it critically for important intellectual content: all authors
Final approval: all authors
Guarantor of this manuscript: HA, MS

Disclosures

Prof. Beer reports grant support from the Swiss National Foundation of Science, The Swiss Heart Foundation and the Stiftung Kardio; grant support, speakers- and consultation fees to the institution from Bayer, Sanofi and Daichii Sankyo. Dr Blozik reports grants from Swiss Cancer Research Foundation; institutional grants from Amgen, MSD, Novartis, Pfizer, all outside the submitted work. Dr Bonati reports personal fees and nonfinancial support from Amgen, grants from AstraZeneca, personal fees and nonfinancial support from Bayer, personal fees from Bristol-Myers Squibb, personal fees from Claret Medical, grants from Swiss National Science Foundation, grants from University of Basel, grants from Swiss Heart Foundation, outside the submitted work. Dr

For peer review only - http://bmjopen.bmj.com/site/about/guidelines.xhtml

Conen received consulting fees from Roche Diagnostics, and speaker fees from Servier and BMS/Pfizer, all outside of the current work. Dr Kühne reports personal fees from Bayer, personal fees from Böhringer Ingelheim, personal fees from Pfizer BMS, personal fees from Daiichi Sankyo, personal fees from Medtronic, personal fees from Biotronik, personal fees from Boston Scientific, personal fees from Johnson&Johnson, personal fees from Roche, grants from Bayer, grants from Pfizer, grants from Boston Scientific, grants from BMS, grants from Biotronik, grants from Daiichi Sankyo. Dr Moschovitis has received consultant fees for taking part to advisory boards from Novartis, Boehringer Ingelheim, Bayer, Astra Zeneca and Daiichi Sankyo, all outside of the presented work. Dr Müller reports fellowship and training support from Biotronik, Boston Scientific, Medtronic, Abbott/St. Jude Medical, and Biosense Webster; speaker honoraria from Biosense Webster, Medtronic, Abbott/St. Jude Medical, AstraZeneca, Daiichi Sankyo, Biotronik, MicroPort, Novartis, and consultant honoraria for Biosense Webster, Medtronic, Abbott/St. Jude Medcal, and Biotronik. Dr Osswald received research grants from the Swiss National Science Foundation and from the Swiss Heart Foundation, research grants from Foundation for CardioVascular Research Basel, research grants from Roche, educational and speaker office grants from Roche, Bayer, Novartis, Sanofi AstraZeneca, Daiichi-Sankyo and Pfizer. Dr Reichlin has received research grants from the Swiss National Science Foundation, the Swiss Heart Foundation, and the sitem insel support fund, all for work outside the submitted study. Speaker/consulting honoraria or travel support from Abbott/SJM, Astra Zeneca, Brahms, Bayer, Biosense-Webster, Biotronik, Boston-Scientific, Daiichi Sankyo, Medtronic, Pfizer-BMS and Roche, all for work outside the submitted study. Support for his institution's fellowship program from Abbott/SJM, Biosense-Webster, Biotronik, Boston-Scientific and Medtronic for work outside the submitted study. Dr Schwenkglenks reports grants from Swiss National Science Foundation, for the conduct of the study; grants and personal fees from Amgen, grants from MSD, grants from Novartis, grants from Pfizer, grants from The Medicines Company, all outside the submitted work. Dr Serra-Burriel reports grants from the European Commission outside of the present work. Dr Sticherling has received speaker honoraria from Biosense Webster and Medtronic and research grants from Biosense Webster, Daiichi-Sankyo, and Medtronic. The remaining authors have nothing to disclose.

Ethics

The Swiss-AF study protocol was approved by the local ethics committee (Ethikkommission Nordwest- und Zentralschweiz, 2014-067, PB_2016-00793), and written informed consent was obtained from each participant.

For peer review only - http://bmjopen.bmj.com/site/about/guidelines.xhtml

The population-based reference data set from Helsana was provided anonymously based on a waiver provided by the competent ethics committee (Kantonale Ethikkommission Zürich, 2020-01346).

Data availability

The Swiss-AF patient informed consent forms state that the data, containing personal and medical information, are exclusively available for research institutions in an anonymized form and are not allowed to be made publicly available. Researchers interested in obtaining the Swiss-AF data for research purposes can contact the Swiss-AF scientific lead. Contact information is provided on the Swiss-AF website (http://www.swissaf.ch/contact.htm). Authorization of the responsible ethics committee is mandatory before the requested data can be transferred to external research institutions.

The population-based reference data set from Helsana was provided anonymously. These claims data cannot be shared publicly because they are the property of Helsana. Considering SNSF policies encouraging data sharing, the data may be shared via Helsana with scientific institutions under specific conditions and considering all data protection rules; final decisions are taken by Helsana. Any such sharing would also require ethical clarification of responsibility and/or clearance, as applicable.

For peer review only

For peer review only - http://bmjopen.bmj.com/site/about/guidelines.xhtml

References

1. Velleca, M., Costa, G., Goldstein, L., Bishara, M. & Ming Boo, L. *A Review of the Burden of Atrial Fibrillation: Understanding the Impact of the New Millennium Epidemic across Europe*. *EMJ EUROPEAN MEDICAL JOURNAL* vol. 110 (2019).
2. Global Burden of Disease (GBD 2019) | Institute for Health Metrics and Evaluation. https://www.healthdata.org/gbd/2019.
3. Jönsson, L., Eliasson, Å., Kindblom, J., Almgren, O. & Edvardsson, N. Cost of illness and drivers of cost in atrial fibrillation in Sweden and Germany. *Appl Health Econ Health Policy* 8, 317–325 (2010).
4. Ringborg, A. *et al.* Costs of atrial fibrillation in five European countries: Results from the Euro Heart Survey on atrial fibrillation. *Europace* 10, 403–411 (2008).
5. Ciminata, G., Geue, C., Langhorne, P. & Wu, O. A two-part model to estimate inpatient, outpatient, prescribing and care home costs associated with atrial fibrillation in Scotland. *BMJ Open* 10, e028575 (2020).
6. le Heuzey, J.-Y. *et al.* Cost of care distribution in atrial fibrillation patients: the COCAF study. *Am Heart J* 147, 121–126 (2004).
7. Patel, N. J. *et al.* Contemporary Trends of Hospitalization for Atrial Fibrillation in the United States, 2000 Through 2010. *Circulation* 129, 2371–2379 (2014).
8. Kim, M., Johnston, S., Chu, B., Dalal, M. R. & Schulman, K. Estimation of total incremental health care costs in patients with atrial fibrillation in the United States. *Circ Cardiovasc Qual Outcomes* 4, 313–320 (2011).
9. McBride, D., Mattenklotz, A. M., Willich, S. N. & Brüggenjürgen, B. The costs of care in atrial fibrillation and the effect of treatment modalities in Germany. *Value in Health* 12, 293–301 (2009).
10. Cotté, F. E. *et al.* Burden of stroke and other cardiovascular complications in patients with atrial fibrillation hospitalized in France. *Europace* 18, 501 (2016).
11. Ericson, L., Bergfeldt, L. & Björholt, I. Atrial fibrillation: the cost of illness in Sweden. *The European Journal of Health Economics* 12, 479–487 (2010).
12. Stewart, S., Murphy, N. F., Walker, A., McGuire, A. & McMurray, JJ. Cost of an emerging epidemic: an economic analysis of atrial fibrillation in the UK. *Heart* 90, 286–292 (2004).
13. Coyne, K. S. *et al.* Assessing the direct costs of treating nonvalvular atrial fibrillation in the United States. *Value in Health* 9, 348–356 (2006).
14. Ball, J., Carrington, M. J., McMurray, J. J. V. & Stewart, S. Atrial fibrillation: Profile and burden of an evolving epidemic in the 21st century. *Int J Cardiol* 167, 1807–1824 (2013).

For peer review only - http://bmjopen.bmj.com/site/about/guidelines.xhtml

15. Burdett, P. & Lip, G. Y. H. Atrial fibrillation in the UK: predicting costs of an emerging epidemic recognizing and forecasting the cost drivers of atrial fibrillation-related costs. *Eur Heart J Qual Care Clin Outcomes* 0, 1–8 (2020).
16. Johnsen, S. P., Dalby, L. W., Täckström, T., Olsen, J. & Fraschke, A. Cost of illness of atrial fibrillation: a nationwide study of societal impact. *BMC Health Serv Res* 17, 1–8 (2017).
17. Conen, D. *et al.* Design of the Swiss Atrial Fibrillation Cohort Study (Swiss-AF): structural brain damage and cognitive decline among patients with atrial fibrillation. *Swiss Med Wkly* 147, (2017).
18. ICD. https://www.who.int/standards/classifications/classification-of-diseases (2021).
19. SwissDRG. https://www.swissdrg.org/de/akutsomatik/swissdrg-system-1002021 (2021).
20. CHOP. https://www.bfs.admin.ch/bfs/de/home/statistiken/kataloge-datenbanken/publikationen.assetdetail.9286150.html (2021).
21. ATC. https://www.swissmedic.ch/swissmedic/en/home/services/listen_neu.html (2021).
22. TARMED. https://www.bag.admin.ch/bag/de/home/versicherungen/krankenversicherung/krankenversicherung-leistungen-tarife/Aerztliche-Leistungen-in-der-Krankenversicherung/Tarifsystem-Tarmed.html (2021).
23. Huber, C. A., Szucs, T. D., Rapold, R. & Reich, O. Identifying patients with chronic conditions using pharmacy data in Switzerland: an updated mapping approach to the classification of medications. *BMC Public Health* 13, 1–10 (2013).
24. Aebersold, H. *et al.* Patient clusters and cost trajectories in the Swiss Atrial Fibrillation cohort. *Heart* heartjnl-2022-321520 (2022) doi:10.1136/HEARTJNL-2022-321520.
25. Belotti, F., Deb, P., Manning, W. G. & Norton, E. C. Twopm: Two-Part Models: *https://doi.org/10.1177/1536867X1501500102* 15, 3–20 (2015).
26. Deb, P. & Norton, E. C. Modeling Health Care Expenditures and Use. *Annu Rev Public Health* 39, 489–505 (2018).
27. Blough, D. K., Madden, C. W. & Hornbrook, M. C. Modeling risk using generalized linear models. *J Health Econ* 18, 153–171 (1999).
28. Smith, V. A., Neelon, B., Maciejewski, M. L. & Preisser, J. S. Two parts are better than one: modeling marginal means of semicontinuous data. *Health Serv Outcomes Res Methodol* 17, 198–218 (2017).

For peer review only

For peer review only - http://bmjopen.bmj.com/site/about/guidelines.xhtml

29. Bundesamt für Statistik | Bundesamt für Statistik. https://www.bfs.admin.ch/bfs/de/home.html.

30. Gouveia, M. *et al.* Burden of disease and cost of illness of atrial fibrillation in Portugal. *Cardiologia* 34, 1–11 (2015).

For peer review only

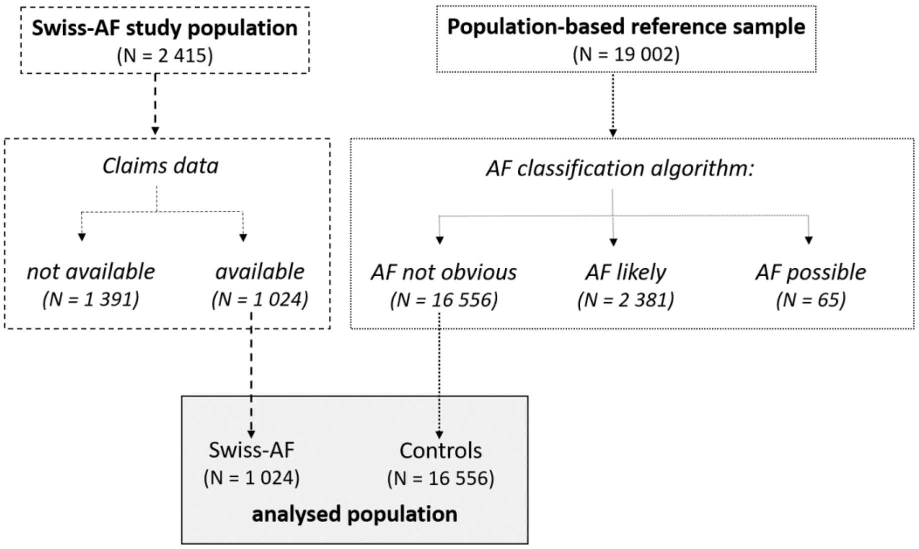

659x401mm (59 x 59 DPI)

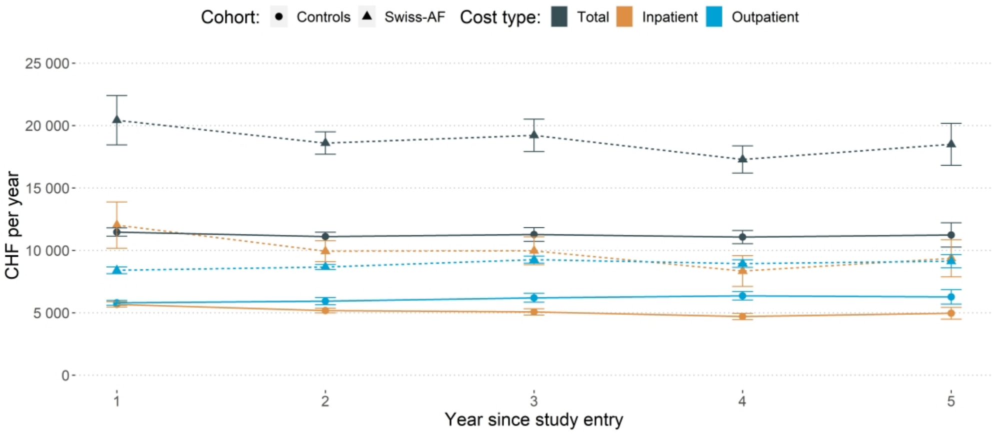

349x159mm (600 x 600 DPI)

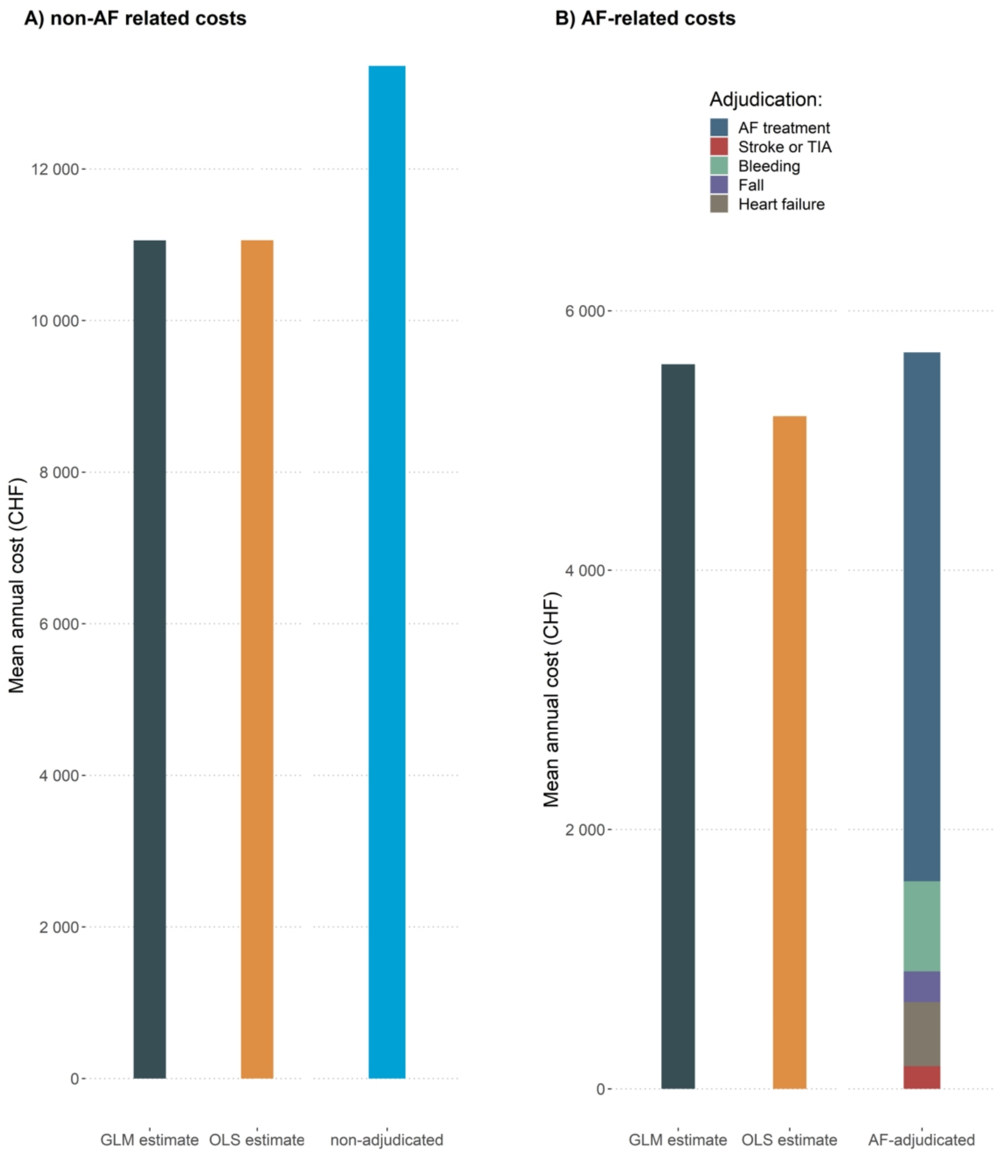

84x99mm (600 x 600 DPI)

Table S1. Sample size determination for the population-based control sample

In the absence of exact solutions for the determination of the required size of the population-based, non-AF control sample, we tried to estimate a plausible magnitude based on published cost studies. Our study aim was to compare the costs of prevalent atrial fibrillation (AF) patients with the costs of controls not having AF. In the absence of published comparisons of this type, we used the AF-attributable versus non-AF-attributable costs of AF patients as a fallback. We found that mean attributable costs may differ by roughly 0.3 standard deviations from the mean of non-attributable costs [1, 2, 3]. Based on the results of Turakhia [4], an expected minimum effect size (Cohen's d) of the cost of AF would be approximately 0.1. Given the possibility of such a small effect size and to be on the safe side we assumed a 50% smaller effect, i.e. Cohen's d of 0.05.

AF patients were planned to be compared to controls differing in several dimensions, and a variety of sub-analyses were planned to be performed to characterize the cost impact of AF. To mimic the impact of this situation on the required size of the control sample, a Bonferroni correction for multiple comparisons was assumed, with an estimated number of 15 hypothesis: checking for divergences in gender, age, accumulation of costs over time in different subgroups, various types of costs etc.

With a standard statistical power function and assuming the parameter values and corrections explained above (Cohen's d = 0.05, number of hypotheses = 15), a sample size of 17'000 valid controls was estimated to be required to obtain a 95% statistical power and a 5% false positive risk. This became the planned size of the non-AF control sample. Considering that some otherwise eligible people would have AF, the size of the full reference sample was inflated to the point where 17'000 non-AF controls were reached.

[1] Jönsson et al. 2010. Cost of Illness and drivers of Cost in Atrial Fibrillation in Sweden and Germany, Applied Health Economics and Health Policy, 8, 317-325, DOI: 10.2165/11319880-000000000-00000

[2] Brüggenjürgen et al. 2007, The Impact of Atrial Fibrillation on the Cost of Stroke: the Berlin Acute Stroke Study, Value in Health, 10:2, 137-143, DOI: 10.1111/j.1524-4733.2006.00160.x

[3] Wodchis et al. 2012. A Review of the Cost of Atrial Fibrillation, Value in Health, 15:2, 240-248, DOI: https://doi.org/10.1016/j.jval.2011.09.009

[4] Turakhia et al. 2015. Economic Burden of Undiagnosed Nonvalvular Atrial Fibrillation in the United States, Am J Cariol, 116:5, 733-739, DOI: 10.1016/j.amjcard.2015.05.045.

For peer review only

Table S2. Algorithm classifying the population-based reference sample as "AF likely" and "AF possible". Individuals with none of the listed criteria present were classified as "AF not obvious" and considered as controls.

code		allocation	
		AF likely	AF possible
ICD10	**I48.0** Vorhofflimmern, paroxysmal	1	
ICD10	**I48.1** Vorhofflimmern, persistierend	1	
ICD10	**I48.2** Vorhofflimmern, permanent	1	
ICD10	**I48.3** Vorhofflattern, typisch	1	
ICD10	**I48.4** Vorhofflattern, atypisch	1	
ICD10	**I48.9** Vorhofflimmern und Vorhofflattern, nicht näher bezeichnet	1	
ICD10	**I49.8** Sonstige näher bezeichnete kardiale Arrhythmien		1
ICD10	**I49.9** Kardiale Arrhythmie, nicht näher bezeichnet		1
DRG	**F50A** Ablative Massnahmen bei Tachyarrhythmie mit bestimmter Ablation und komplexem Eingriff, Alter < 16 Jahre		1
DRG	**F50D** Ablative Massnahmen bei Tachyarrhythmie, Alter > 15 Jahre		1
ICD10 + DRG	ICD **I48 +** DRG **F50A**	1	
ICD10 + DRG	ICD **I48 +** DRG **F50D**	1	
CHOP	**Z37.34.24** Lokalisationen bei Ablationsverfahren bei Tachyarrhythmien	1	
CHOP	**Z99.61** Vorhofskardioversion	1	
CHOP	**Z99.62** Externe Kardioversion	1	
Tarmed	**17.1510** Kardioversion bei Vorhofflimmern/Vorhofflattern, als alleinige Leistung	1	
ATC	**C01BD07** Dronedarone (Multaq)	1	

Notes: Abbreviations: AF: atrial fibrillation, ATC: anatomical therapeutic chemical classification, CHOP: Swiss invasive medical procedures catalogue, DRG: diagnosis related group, ICD10: international classification of diseases (10th revision).

For peer review only

Figure S1. Trajectories of monthly costs and Kaplan-Meier curves by cohort.

A)

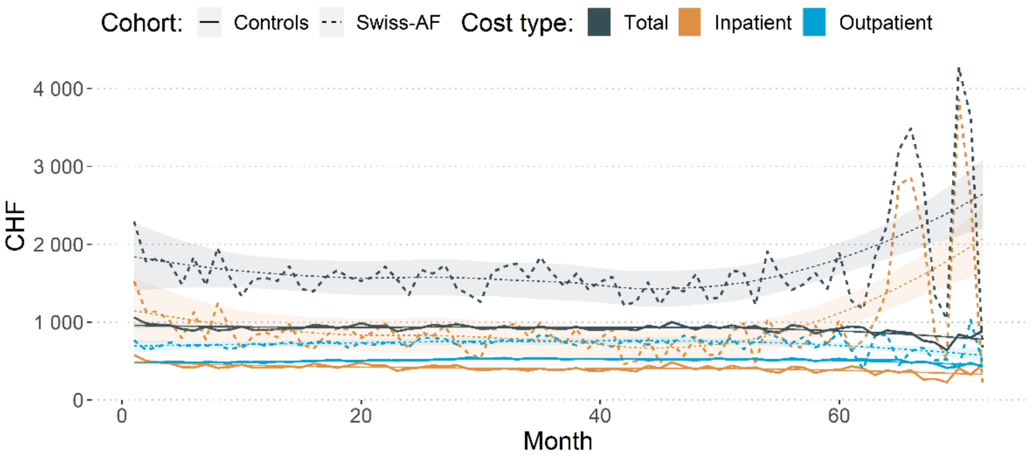

B)

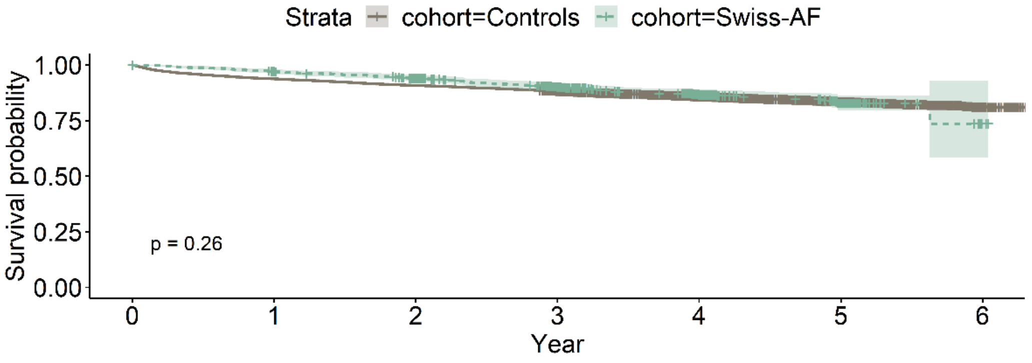

C) N at risk

Number at risk

Strata \ Year	0	1	2	3	4	5	6
cohort=Controls	16556	15532	15041	14279	8714	4299	385
cohort=Swiss-AF	1024	982	920	706	375	136	2

D) deaths

Cumulative number of events

Strata \ Year	0	1	2	3	4	5	6
cohort=Controls	5	1026	1515	1939	2271	2439	2504
cohort=Swiss-AF	0	29	60	96	117	126	127

E) loss to FU

Cumulative number of events

Strata \ Year	0	1	2	3	4	5	6
cohort=Controls	0	0	0	0	0	0	0
cohort=Swiss-AF	7	14	23	28	34	37	39

F) admin. censoring

Cumulative number of events

Strata \ Year	0	1	2	3	4	5	6
cohort=Controls	0	0	0	357	5572	9844	13675
cohort=Swiss-AF	0	0	26	212	505	735	856

Notes: median (interquartile range IQR) follow-up: Swiss-AF 3.41 (1.08) years, controls 4.10 (1.72) years; total patient-years of follow-up: SAF 3 571.24, cohort 66 068.24.

Table S3. Observed annual costs in CHF by cost component and cohort.

	Swiss-AF		***Controls***	
Cost component	*Median [IQR]*	*Mean (SD)*	*Median [IQR]*	*Mean (SD)*
Total	4 518 [825, 11 771]	19 037 (59 998)	2 135 [0, 7 473]	11 192 (38 939)
Total inpatient	0 [0, 0]	10 235 (56 327)	0 [0, 0]	5 077 (34 925)
Total outpatient drugs	508 [0, 29 56]	2 495 (7 382)	235 [0, 1 781]	1 984 (7 852)
Total outpatient without drugs	2 282 [59, 7 225]	6 307 (13 154)	801 [0, 4 310]	4 131 (10 260)
Total AF-adj.	400 [0, 3 213]	5 679 (36 135)	*NA*	*NA*
Total AF-adj. inpatient	0 [0, 0]	3 458 (35 188)	*NA*	*NA*
Total AF-adj. outpatient drugs	0 [0, 250]	591 (1 392)	*NA*	*NA*
Total AF-adj. outpatient without drugs	0 [0, 1 251]	1 630 (6 899)	*NA*	*NA*
AF-adjudication:				
Total AF treatment	226 [0, 2 773]	4 078 (2 8640)	*NA*	*NA*
Total stroke or TIA	0 [0, 0]	174 (9124)	*NA*	*NA*
Total bleeding	0 [0, 0]	696 (17462)	*NA*	*NA*
Total fall	0 [0, 0]	237 (4434)	*NA*	*NA*
Total heart failure	0 [0, 0]	494 (8469)	*NA*	*NA*

Abbreviations: adj.: adjudicated, AF: atrial fibrillation, IQR: interquartile range, PCG: pharmaceutical cost groups, SD: standard deviation, SMD: standardized mean difference.

For peer review only

Figure S2. Boxplot distribution of mean annual costs by cost outcome and cohort.

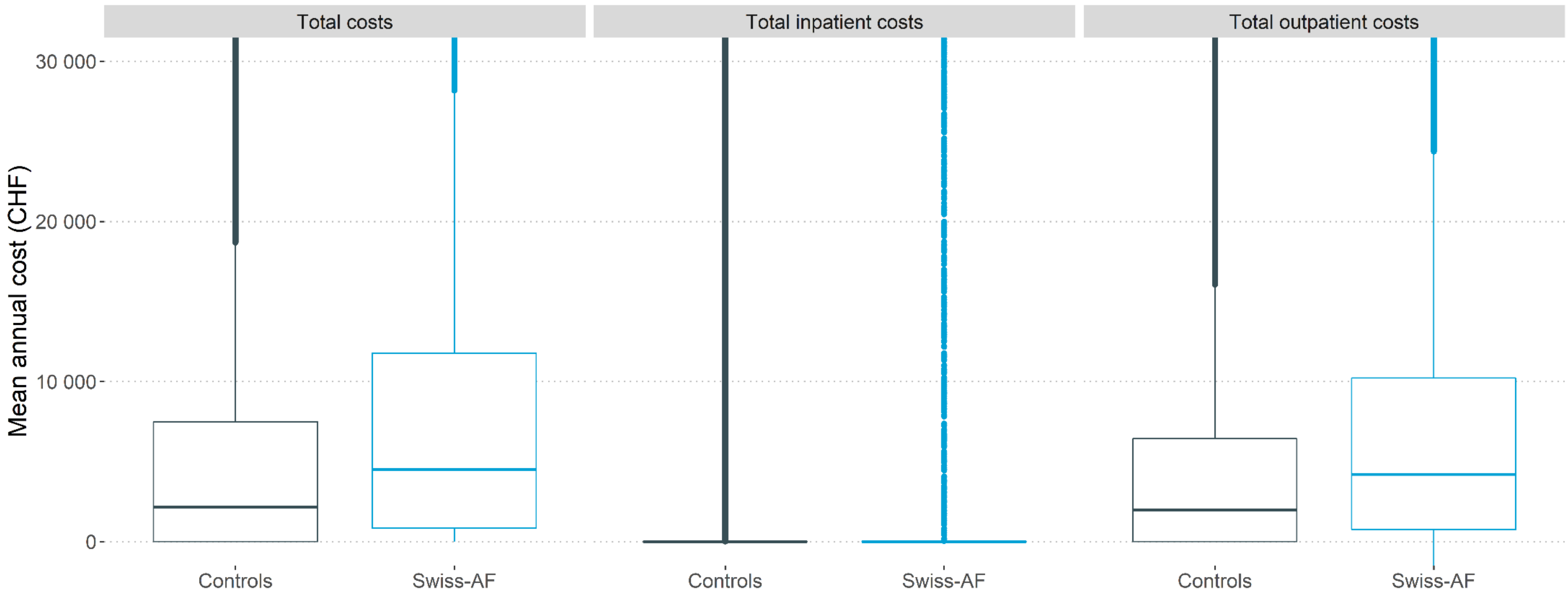

Table S4. Regression results from GLM-based two part modelling.

	Total costs		Outpatient costs		Inpatient costs	
	Odds ratio (Logistic part)	*Marginal effect (GLM part)*	*Odds ratio (Logistic part)*	*Marginal effect (GLM part)*	*Odds ratio (Logistic part)*	*Marginal effect (GLM part)*
Cohort: Swiss-AF	1.5 [1.46, 1.54]	6 374 [5 609, 7 139]	1.46 [1.42, 1.5]	1 299 [1 097, 1 501]	1.13 [1.08, 1.17]	35 154 [28 827, 41 481]
Month	1.00 [1.00, 1.00]	29 [20, 38]	1.00 [1.00, 1.00]	35 [33, 38]	1.00 [1.00, 1.00]	- 335 [- 408, -262]
Age	1.03 [1.03, 1.03]	242 [223, 260]	1.03 [1.02, 1.03]	36 [30, 41]	1.09 [1.09, 1.09]	- 3 075 [- 3 244, - 2 906]
Sex: Male	0.92 [0.91, 0.93]	3 254 [2 963, 3 545]	0.92 [0.91, 0.93]	1 485 [1 393, 1 577]	1.05 [1.02, 1.07]	10 449 [8 031, 12 866]
PCG acid related disorders	1.69 [1.66, 1.72]	2 610 [2 231, 2 989]	1.66 [1.63, 1.69]	1 487 [1 367, 1 606]	1.30 [1.27, 1.34]	- 3 545 [- 6 209, - 882]
PCG bone diseases	1.94 [1.87, 2.01]	5 278 [4 418, 6 138]	1.91 [1.84, 1.98]	4 517 [4 214, 4 821]	0.97 [0.93, 1.02]	18 455 [12 539, 24 372]
PCG cancer	2.12 [2.03, 2.21]	16 094 [14 613, 17 575]	2.09 [2, 2.18]	12 834 [12 269, 13 399]	1.22 [1.16, 1.29]	18 812 [11 626, 25 999]
PCG cardio	1.89 [1.87, 1.91]	- 402 [- 738, - 66]	1.90 [1.87, 1.92]	317 [214, 419]	0.98 [0.96, 1.01]	8 045 [5 312, 10 779]
PCG dementia	2.05 [1.98, 2.13]	1 819 [1 166, 2 471]	1.93 [1.87, 2]	949 [745, 1 154]	2.14 [2.07, 2.22]	- 26 586 [- 29 160, - 24 012]
PCG diabetes	1.67 [1.64, 1.7]	3 790 [3 355, 4 225]	1.66 [1.63, 1.69]	2 220 [2 083, 2 358]	1.25 [1.21, 1.28]	5 212 [1 986, 8 439]
PCG epilepsy	2.26 [2.18, 2.34]	5 403 [4 703, 6 103]	2.19 [2.12, 2.27]	2 636 [2 421, 2 851]	1.58 [1.53, 1.63]	- 3 375 [- 6 723, - 28]
PCG glaucoma	1.56	- 493	1.56	638	0.87	- 1 171

For peer review only

	[1.53, 1.59]	[- 902, - 83]	[1.53, 1.59]	[500, 776]	[0.85, 0.9]	[- 4 610, 2 268]
PCG gout	1.35<br>[1.32, 1.39]	1 296<br>[713, 1 878]	1.36<br>[1.32, 1.39]	509<br>[329, 688]	1.02<br>[0.98, 1.06]	7 684<br>[27 98, 12 571]
PCG hyperlipidemia	1.32<br>[1.3, 1.33]	- 1 142<br>[- 1 437, - 847]	1.32<br>[1.3, 1.33]	- 275<br>[- 368, - 182]	0.86<br>[0.84, 0.88]	5 201<br>[2 606, 7 796]
PCG iron deficiency	1.57<br>[1.51, 1.64]	5 284<br>[4 401, 6 167]	1.56<br>[1.5, 1.62]	3 695<br>[3 401, 3 989]	1.20<br>[1.15, 1.25]	2 373<br>[- 2 429, 7 176]
PCG pain	1.47<br>[1.44, 1.5]	6 097<br>[5 628, 6 567]	1.45<br>[1.42, 1.49]	2 326<br>[2 186, 2 465]	1.82<br>[1.78, 1.87]	- 3 500<br>[- 6 189, - 812]
PCG psychiatric	2.19<br>[2.15, 2.24]	1 909<br>[1 534, 2285]	2.17<br>[2.12, 2.21]	1 048<br>[930, 1 167]	1.60<br>[1.56, 1.64]	- 15 150<br>[- 17 606, - 12 693]
PCG antipsychotic	2.92<br>[2.78, 3.06]	9 281<br>[8278, 1 0285]	2.50<br>[2.39, 2.62]	1 347<br>[1 108, 1 586]	5.98<br>[5.8, 6.17]	- 39 595<br>[- 41 844, - 37 345]
PCG respiratory	1.67<br>[1.64, 1.7]	2 124<br>[1 689, 2 558]	1.66<br>[1.63, 1.69]	1 192<br>[1 055, 1 328]	1.09<br>[1.06, 1.12]	9 644<br>[6 096, 13 193]
PCG rheumatic conditions	1.64<br>[1.61, 1.66]	- 1<br>[- 332, 330]	1.64<br>[1.62, 1.67]	458<br>[352, 564]	0.85<br>[0.82, 0.87]	8 762<br>[5 847, 11 677]
PCG thyroid disorders	1.47<br>[1.43, 1.51]	- 306<br>[- 850, 237]	1.47<br>[1.43, 1.51]	454<br>[273, 635]	0.90<br>[0.87, 0.94]	2 587<br>[- 2 050, 7 223]
PCG other rare diseases	2.26<br>[2.17, 2.35]	4 675<br>[3 889, 5 462]	2.22<br>[2.14, 2.3]	3022<br>[2 766, 3 277]	1.54<br>[1.49, 1.6]	- 1 274<br>[- 5 112, 2 564]
Urbanisation: agglomeration	0.97<br>[0.96, 0.99]	- 115<br>[- 436, 206]	0.98<br>[0.97, 0.99]	- 154<br>[- 256, - 53]	0.98<br>[0.96, 1]	1 242<br>[- 1 460, 3 945]
Urbanisation: rural	0.91<br>[0.9, 0.92]	- 44<br>[- 461, 374]	0.91<br>[0.9, 0.92]	- 307<br>[- 437, - 177]	1.13<br>[1.09, 1.16]	- 8 008<br>[- 11 371, - 4 645]
Greater Region: Lake Geneva	1.2<br>[1.17, 1.23]	2 819<br>[2 116, 3 523]	1.19<br>[1.16, 1.22]	2 131<br>[1 899, 2 362]	1.01<br>[0.97, 1.06]	18 469<br>[13 431, 23 508]
	1.07	- 771	1.06	- 499	0.82	8 683

For peer review only

Greater Region: Espace Mittelland	[1.05, 1.09]	[- 1 247, - 295]	[1.04, 1.08]	[- 650, - 348]	[0.79, 0.85]	[4 836, 12 529]
Greater Region: Northwestern Switzerland	1.1<br>[1.09, 1.12]	656<br>[217, 1 096]	1.10<br>[1.09, 1.12]	- 134<br>[- 272, 3]	0.95<br>[0.92, 0.98]	11 761<br>[8 460, 15 063]
Greater Region: Eastern Switzerland	0.93<br>[0.91, 0.96]	- 504<br>[- 1 182, 173]	0.94<br>[0.91, 0.96]	- 900<br>[- 1 105, - 695]	0.97<br>[0.92, 1.02]	6 304<br>[747, 11 861]
Greater Region: Southern Switzerland	1.26<br>[1.24, 1.29]	-720<br>[- 1 247, - 193]	1.26<br>[1.24, 1.29]	241<br>[68, 414]	0.67<br>[0.64, 0.7]	20 870<br>[15 771, 25 969]
Greater Region: Central Switzerland	0.94<br>[0.91, 0.96]	- 676<br>[- 1 379, 26]	0.93<br>[0.91, 0.95]	- 237<br>[- 463, - 11]	0.91<br>[0.86, 0.96]	- 3 079<br>[- 8 627, 2 470]
Observations	798 940	545 385	798 940	543 131	798 940	47 028

Notes: The brackets show 95% confidence intervals. Abbreviations: AF: atrial fibrillation, GLM: generalized linear model, PCG: pharmaceutical cost groups.

For peer review only

Table S5. Regression results from OLS-based two part modelling.

	Total costs		Outpatient costs		Inpatient costs	
	Odds ratio (logistic part)	*Cost estimate (OLS part)*	*Odds ratio (logistic part)*	*Cost estimate (OLS part)*	*Odds ratio (logistic part)*	*Cost estimate (OLS part)*
Cohort: Swiss-AF	1.5 [1.46, 1.54]	5 744 [5 210, 6 277]	1.46 [1.42, 1.5]	1 043 [860, 1 226]	1.13 [1.08, 1.17]	37 322 [32 916, 41 728]
Month	1.00 [1.00, 1.00]	5.47 [- 2.49, 13.43]	1.00 [1.00, 1.00]	26.33 [23.61, 29.06]	1.00 [1.00, 1.00]	- 330.16 [- 397.79, -262.52]
Age	1.03 [1.03, 1.03]	208 [191, 225]	1.03 [1.02, 1.03]	12 [6, 17]	1.09 [1.09, 1.09]	- 2 833 [- 2 975, - 2 690]
Sex: Male	0.92 [0.91, 0.93]	3 378 [3 090, 3 666]	0.92 [0.91, 0.93]	1 802 [1 703, 1 901]	1.05 [1.02, 1.07]	9 021 [6 663, 11 379]
PCG acid related disorders	1.69 [1.66, 1.72]	2 568 [2 239, 2 896]	1.66 [1.63, 1.69]	1 454 [1 341, 1 566]	1.30 [1.27, 1.34]	- 1 799 [- 4 324, 726]
PCG bone diseases	1.94 [1.87, 2.01]	6 789 [6 167, 7 411]	1.91 [1.84, 1.98]	5 529 [5 316, 5 742]	0.97 [0.93, 1.02]	13 650 [9 006, 18 294]
PCG cancer	2.12 [2.03, 2.21]	17 579 [16 855, 18 302]	2.09 [2, 2.18]	14 032 [13 784, 14 279]	1.22 [1.16, 1.29]	15 126 [9 514, 20 738]
PCG cardio	1.89 [1.87, 1.91]	- 325 [- 633, - 16]	1.90 [1.87, 1.92]	339 [234, 445]	0.98 [0.96, 1.01]	7 699 [5 040, 10 358]
PCG dementia	2.05 [1.98, 2.13]	1 897 [1 343, 2 451]	1.93 [1.87, 2]	869 [679, 1 060]	2.14 [2.07, 2.22]	- 23 773 [- 26 889, - 20 657]
PCG diabetes	1.67 [1.64, 1.7]	3 847 [3 497, 4 198]	1.66 [1.63, 1.69]	2 435 [2 315, 2 555]	1.25 [1.21, 1.28]	5 543 [2 641, 8 446]
PCG epilepsy	2.26 [2.18, 2.34]	6 908 [6 395, 7 421]	2.19 [2.12, 2.27]	3 450 [3 274, 3 626]	1.58 [1.53, 1.63]	- 8 073 [- 11 290, - 4 856]
PCG glaucoma	1.56	- 638	1.56	541	0.87	- 832

For peer review only

	[1.53, 1.59]	[- 1 028, - 249]	[1.53, 1.59]	[407, 674]	[0.85, 0.9]	[- 4 077, 2 413]
PCG gout	1.35 [1.32, 1.39]	1 766 [1 258, 2 275]	1.36 [1.32, 1.39]	806 [632, 980]	1.02 [0.98, 1.06]	8 036 [3 787, 12 285]
PCG hyperlipidemia	1.32 [1.3, 1.33]	- 1 269 [- 1 545, - 992]	1.32 [1.3, 1.33]	- 493 [- 588, - 398]	0.86 [0.84, 0.88]	4 874 [2 483, 7 266]
PCG iron deficiency	1.57 [1.51, 1.64]	6 803 [6 164, 7 442]	1.56 [1.5, 1.62]	4 636 [4 417, 4 855]	1.20 [1.15, 1.25]	1 784 [- 2 597, 6 165]
PCG pain	1.47 [1.44, 1.5]	6 773 [6 412, 7 134]	1.45 [1.42, 1.49]	2 817 [2 693, 2 941]	1.82 [1.78, 1.87]	- 2 141 [- 4 690, 407]
PCG psychiatric	2.19 [2.15, 2.24]	2 214 [1 883, 2 544]	2.17 [2.12, 2.21]	1 306 [1 193, 1 419]	1.60 [1.56, 1.64]	- 15 352 [- 17 778, - 12 926]
PCG antipsychotic	2.92 [2.78, 3.06]	9 387 [8 764, 10 009]	2.50 [2.39, 2.62]	1 256 [1 042, 1 470]	5.98 [5.8, 6.17]	- 34 176 [- 37 082, - 31 269]
PCG respiratory	1.67 [1.64, 1.7]	2 062 [1 692, 2 433]	1.66 [1.63, 1.69]	1 180 [1 053, 1 307]	1.09 [1.06, 1.12]	5 583 [2 507, 8 660]
PCG rheumatic conditions	1.64 [1.61, 1.66]	- 648 [- 956, - 341]	1.64 [1.62, 1.67]	26 [- 79, 131]	0.85 [0.82, 0.87]	7 306 [4 712, 9 900]
PCG thyroid disorders	1.47 [1.43, 1.51]	72 [- 442, 585]	1.47 [1.43, 1.51]	495 [319, 671]	0.90 [0.87, 0.94]	2 701 [- 1 520, 6 922]
PCG other rare diseases	2.26 [2.17, 2.35]	4 566 [3 979, 5 152]	2.22 [2.14, 2.3]	2 874 [2 673, 3 075]	1.54 [1.49, 1.6]	- 5 617 [- 9 245, - 1 989]
Urbanisation: agglomeration	0.97 [0.96, 0.99]	- 213 [- 511, 85]	0.98 [0.97, 0.99]	- 279 [- 381, - 177]	0.98 [0.96, 1]	1 560 [- 927, 4 046]
Urbanisation: rural	0.91 [0.9, 0.92]	- 416 [- 803, - 29]	0.91 [0.9, 0.92]	- 501 [- 634, - 369]	1.13 [1.09, 1.16]	- 6 086 [- 9 376, - 2 796]
Greater Region: Lake Geneva	1.2 [1.17, 1.23]	3 425 [2 832, 4 018]	1.19 [1.16, 1.22]	2 319 [2 116, 2 522]	1.01 [0.97, 1.06]	15 877 [11 270, 20 483]
	1.07	- 899	1.06	- 583	0.82	7 964

For peer review only

Greater Region: Espace Mittelland	[1.05, 1.09]	[- 1 353, - 445]	[1.04, 1.08]	[- 739, - 428]	[0.79, 0.85]	[4 098, 11 830]
Greater Region: Northwestern Switzerland	1.1<br>[1.09, 1.12]	493<br>[85, 901]	1.10<br>[1.09, 1.12]	- 255<br>[- 395, - 115]	0.95<br>[0.92, 0.98]	12 598<br>[9 256, 15 939]
Greater Region: Eastern Switzerland	0.93<br>[0.91, 0.96]	- 330<br>[- 979, 319]	0.94<br>[0.91, 0.96]	- 857<br>[- 1 079, - 634]	0.97<br>[0.92, 1.02]	1 036<br>[- 4 494, 6 567]
Greater Region: Southern Switzerland	1.26<br>[1.24, 1.29]	- 803<br>[- 1 308, - 298]	1.26<br>[1.24, 1.29]	171<br>[- 2, 344]	0.67<br>[0.64, 0.7]	13 761<br>[9 162, 18 360]
Greater Region: Central Switzerland	0.94<br>[0.91, 0.96]	- 781<br>[- 1 460, - 101]	0.93<br>[0.91, 0.95]	- 167<br>[- 399, 66]	0.91<br>[0.86, 0.96]	- 1 150<br>[- 7 175, 4 875]
Observations	798 940	545 385	798 940	543 131	798 940	47 028

Notes: The brackets show 95% confidence intervals. Abbreviations: AF: atrial fibrillation, OLS: ordinary least square, PCG: pharmaceutical cost groups.

For peer review only

Table S6. Comparison of cohort characteristics before and after propensity score matching.

	Before propensity score matching (1:1)				*After propensity score matching (1:1)*			
	**Swiss-AF**	**Controls**			**Swiss-AF**	**Controls**		
N	1 024	16 556	p	SMD	958	958	p	SMD
**Characteristics**								
Age *mean (SD)*	73.04 (8.17)	72.64 (8.52)	0.139	0.049	73.01 (8.20)	72.96 (8.37)	0.908	0.005
Sex: Male *N (%)*	741 (72.4)	11766 (71.1)	0.394	0.029	694 (72.4)	652 (68.1)	0.04	0.096
**Comorbidities (PCG)** *N (%)*								
Acid related	397 (38.8)	2802 (17.4)	<0.001	0.491	372 (38.8)	387 (40.4)	0.513	0.032
Bone	44 (4.3)	644 (4.0)	0.719	0.014	43 (4.5)	42 (4.4)	1	0.005
Cancer	35 (3.4)	510 (3.2)	0.748	0.013	33 (3.4)	29 (3.0)	0.699	0.024
Cardiovascular	754 (73.8)	10381 (63.7)	<0.001	0.22	706 (73.7)	676 (70.6)	0.14	0.07
Dementia	27 (2.6)	797 (5.0)	0.001	0.122	27 (2.8)	28 (2.9)	1	0.006
Diabetes	122 (11.9)	2298 (14.3)	0.04	0.07	110 (11.5)	101 (10.5)	0.559	0.03
Epilepsy	66 (6.5)	982 (6.1)	0.719	0.014	64 (6.7)	67 (7.0)	0.856	0.012
Glaucoma	103 (10.1)	1634 (10.2)	0.939	0.004	98 (10.2)	115 (12.0)	0.245	0.056
Gout	96 (9.4)	935 (5.8)	<0.001	0.134	89 (9.3)	87 (9.1)	0.937	0.007
Hyperlipidemia	425 (41.6)	5649 (35.0)	<0.001	0.136	395 (41.2)	371 (38.7)	0.283	0.051
Iron deficiency	66 (6.5)	567 (3.5)	<0.001	0.134	60 (6.3)	62 (6.5)	0.925	0.009
Pain	386 (37.8)	2484 (15.4)	<0.001	0.523	363 (37.9)	358 (37.4)	0.85	0.011
Psychiatric	266 (26.0)	2837 (17.6)	<0.001	0.204	250 (26.1)	269 (28.1)	0.355	0.045
Antipsychotic	16 (1.6)	878 (5.5)	<0.001	0.213	16 (1.7)	15 (1.6)	1	0.008
Respiratory	144 (14.1)	1915 (11.9)	0.045	0.064	137 (14.3)	148 (15.4)	0.521	0.032
Rheumatic	406 (39.7)	3074 (19.1)	<0.001	0.465	378 (39.5)	378 (39.5)	1	<0.001
Thyroid	87 (8.5)	908 (5.7)	<0.001	0.111	78 (8.1)	88 (9.2)	0.465	0.037
Other rare diseases	27 (2.6)	696 (4.4)	0.011	0.093	27 (2.8)	20 (2.1)	0.376	0.047
**Socioeconomic**								
Mother tongue *N (%)*			0.001	0.116			0.253	0.076
German	755 (73.7)	12944 (78.2)			737 (76.9)	759 (79.2)		
French	141 (13.8)	1708 (10.3)			132 (13.8)	108 (11.3)		

For peer review only

Italian	128 (12.5)	1904 (11.5)			89 (9.3)	91 (9.5)		
Urbanisation *N (%)*			0.236	0.056			0.973	0.011
Urban	253 (26.2)	4330 (26.2)			252 (26.3)	250 (26.1)		
Agglomeration	500 (51.9)	8953 (54.1)			497 (51.9)	502 (52.4)		
Rural	211 (21.9)	3273 (19.8)			209 (21.8)	206 (21.5)		
**Greater Region *N (%)***			<0.001	0.167			0.994	0.038
Zurich	125 (12.2)	2083 (12.6)			120 (12.5)	128 (13.4)		
Lake Geneva Region	56 (5.5)	1086 (6.6)			53 (5.5)	53 (5.5)		
Espace Mitelland	289 (28.2)	3702 (22.4)			278 (29.0)	266 (27.8)		
Northwestern Switzerland	310 (30.3)	5990 (36.2)			307 (32.0)	308 (32.2)		
Eastern Switzerland	67 (6.5)	944 (5.7)			66 (6.9)	66 (6.9)		
Southern Switzerland	125 (12.2)	1904 (11.5)			86 (9.0)	91 (9.5)		
Central Switzerland	52 (5.1)	847 (5.1)			48 (5.0)	46 (4.8)		

Notes: Abbreviations: AF: atrial fibrillation, PCG: pharmaceutical cost groups, SD: standard deviation, SMD: standardized mean difference.

For peer review only

Table S7. Regression results from ordinary (single-part) OLS modelling.

	Total costs	Outpatient costs	Inpatient costs
Cohort: Swiss-AF	5 124<br>[4 726, 5 522]	1 125<br>[986, 1 263]	3 999<br>[3 636, 4 362]
Month	8<br>[3, 14]	20<br>[18, 22]	- 12<br>[- 17, - 7]
Age	201<br>[190, 212]	44<br>[40, 47]	158<br>[147, 168]
Sex: Male	2 197<br>[1 996, 2 398]	1 158<br>[1 088, 1 228]	1 039<br>[856, 1 223]
PCG acid related disorders	3 206<br>[2 953, 3 458]	1 780<br>[1 692, 1 868]	1 426<br>[1 195, 1 656]
PCG bone diseases	6 983<br>[6 502, 7 465]	5 330<br>[5 162, 5 497]	1 653<br>[1 214, 2 093]
PCG cancer	16 504<br>[15 944, 1 7063]	12 765<br>[12 570, 12 960]	3 738<br>[3 228, 4 249]
PCG cardio	1 379<br>[1 171, 1 587]	1 118<br>[1 045, 1 190]	261<br>[71, 451]
PCG dementia	2 907<br>[2 472, 3 342]	1 320<br>[1 168, 1 471]	1 587<br>[1 190, 1 984]
PCG diabetes	4 195<br>[3 930, 4 460]	2 599<br>[2 507, 2 691]	1 596<br>[1 354, 1 838]
PCG epilepsy	7 533<br>[7 127, 7 938]	3 836<br>[3 694, 3 977]	3 697<br>[3 327, 4 067]
PCG glaucoma	434<br>[143, 725]	932<br>[830, 1 033]	- 497<br>[- 763, - 232]
PCG gout	2 168	1 061	1 107

For peer review only - http://bmjopen.bmj.com/site/about/guidelines.xhtml

	[1 784, 2 553]	[927, 1 195]	[756, 1 458]
PCG hyperlipidemia	- 396<br>[- 598, - 194]	- 40<br>[- 110, 30]	- 356<br>[- 540, - 171]
PCG iron deficiency	6 671<br>[6 173, 7 170]	4 392<br>[4 219, 4 566]	2 279<br>[1 824, 2 734]
PCG pain	6 620<br>[6 341, 6 899]	2 821<br>[2 724, 2 919]	3 799<br>[3 544, 4 054]
PCG psychiatric	3 328<br>[3 072, 3 584]	1 907<br>[1 818, 1 996]	1 421<br>[1 188, 1 654]
PCG antipsychotic	10 213<br>[9 717, 10 709]	1 960<br>[1 787, 2 132]	8 254<br>[7 800, 8 707]
PCG respiratory	2 669<br>[2 390, 2 949]	1 520<br>[1 423, 1 617]	1 149<br>[894, 1 404]
PCG rheumatic conditions	328<br>[96, 561]	528<br>[447, 609]	- 200<br>[- 412, 12]
PCG thyroid disorders	656<br>[268, 1 044]	724<br>[589, 859]	- 68<br>[- 422, 286]
PCG other rare diseases	5 353<br>[4 894, 5 812]	3 268<br>[3 108, 3 428]	2 084<br>[1 665, 2 503]
Urbanisation: agglomeration	- 205<br>[- 412, 2]	- 217<br>[- 289, - 145]	12<br>[- 177, 202]
Urbanisation: rural	-503<br>[-768, -237]	-455<br>[-548, -363]	-48<br>[-290, 195]
Greater Region: Lake Geneva	3 002<br>[2 585, 3 419]	1 963<br>[1 818, 2 108]	1 038<br>[658, 1 419]
Greater Region: Espace Mittelland	- 406<br>[- 716, - 97]	- 267<br>[- 375, - 160]	- 139<br>[- 421, 144]
	592	- 29	620

For peer review only

Greater Region: Northwestern Switzerland	[313, 870]	[- 126, 68]	[366, 875]
Greater Region: Eastern Switzerland	- 394<br>[- 826, 39]	- 623<br>[- 773, - 472]	229<br>[-166, 624]
Greater Region: Southern Switzerland	9<br>[- 342, 360]	475<br>[353, 598]	- 466<br>[- 787, - 146]
Greater Region: Central Switzerland	- 610<br>[- 1 059, - 162]	- 197<br>[- 354, - 41]	- 413<br>[- 823, - 3]
Observations	798 940	798 940	798 940

Notes: The brackets show 95% confidence intervals. Abbreviations: AF: atrial fibrillation, OLS: ordinary least square, PCG: pharmaceutical cost groups.

For peer review only

Figure S3. Cost of illness estimated total AF costs per year, in Switzerland and per inhabitant in the general population.

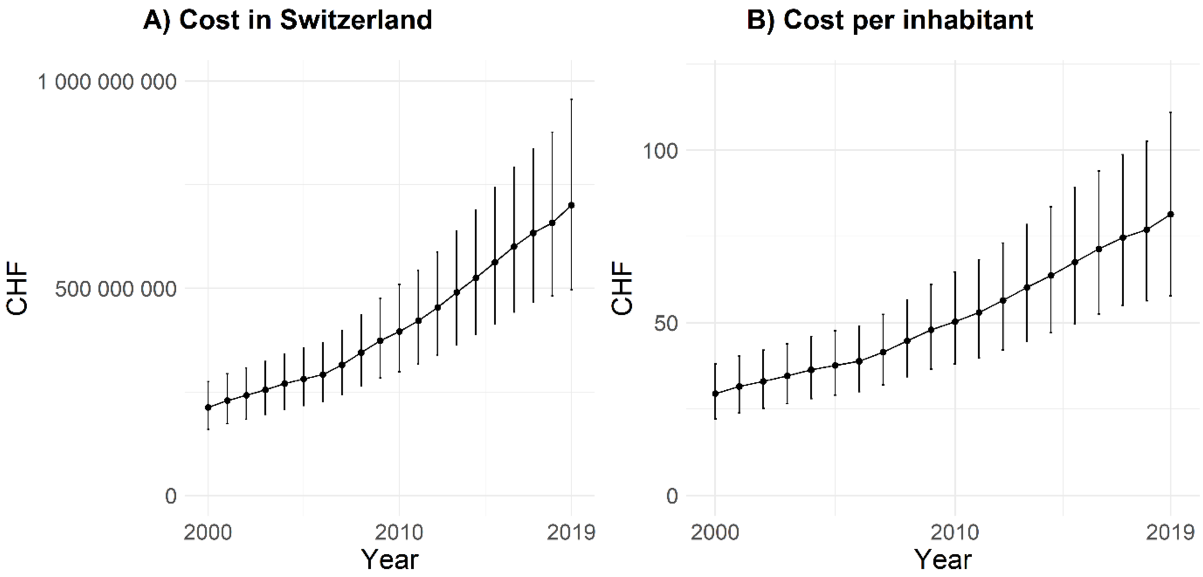

For peer review only

Figure S4. Cost of illness by sex and age group: estimated total AF costs per year, in Switzerland and per inhabitant in the general population.

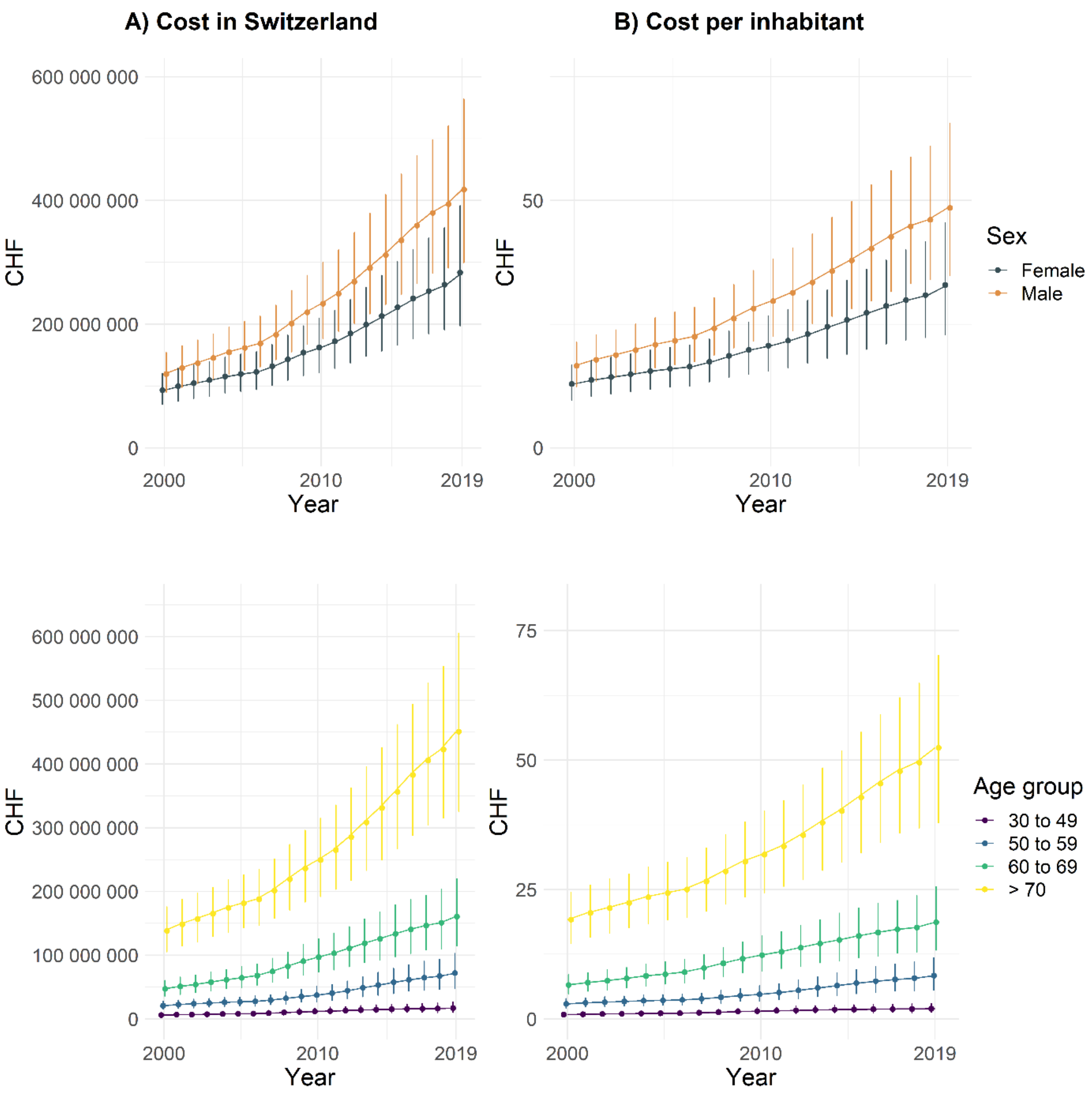

Swiss-AF investigators

University Hospital Basel and Basel University: Stefanie Aeschbacher, Katalin Bhend, Steffen Blum, Leo Bonati, David Conen, Ceylan Eken, Urs Fischer, Corinne Girroy, Elisa Hennings, Elena Herber, Vasco Iten, Philipp Krisai, Michael Kühne, Maurin Lampart, Mirko Lischer, Nina Mäder, Christine Meyer-Zürn, Pascal Meyre, Andreas U. Monsch, Luke Mosher, Christian Müller, Stefan Osswald, Rebecca E. Paladini, Anne Springer, Christian Sticherling, Thomas Szucs, Gian Völlmin. Principal Investigator: Stefan Osswald; Local Principal Investigator: Michael Kühne

University Hospital Bern: Faculty: Faculty: Drahomir Aujesky, Juerg Fuhrer, Laurent Roten, Simon Jung, Heinrich Mattle; Research fellows: Seraina Netzer, Luise Adam, Carole Elodie Aubert, Martin Feller, Axel Loewe, Elisavet Moutzouri, Claudio Schneider; Study nurses: Tanja Flückiger, Cindy Groen, Lukas Ehrsam, Sven Hellrigl, Alexandra Nuoffer, Damiana Rakovic, Nathalie Schwab, Rylana Wenger, Tu Hanh Zarrabi Saffari. Local Principal Investigator: Nicolas Rodondi, Tobias Reichlin

Stadtspital Triemli Zurich: Christopher Beynon, Roger Dillier, Michèle Deubelbeiss, Franz Eberli, Christine Franzini, Isabel Juchli, Claudia Liedtke, Samira Murugiah, Jacqueline Nadler, Thayze Obst, Jasmin Roth, Fiona Schlomowitsch, Xiaoye Schneider, Katrin Studerus, Noreen Tynan, Dominik Weishaupt. Local Principal Investigator: Andreas Müller

Kantonspital Baden: Simone Fontana, Corinne Friedli, Silke Kuest, Karin Scheuch, Denise Hischier, Nicole Bonetti, Alexandra Grau, Jonas Villinger, Eva Laube, Philipp Baumgartner, Mark Filipovic, Marcel Frick, Giulia Montrasio, Stefanie Leuenberger, Franziska Rutz. Local Principal Investigator: Jürg-Hans Beer

Cardiocentro Lugano: Angelo Auricchio, Adriana Anesini, Cristina Camporini, Maria Luce Caputo, Francois Regoli, Martina Ronchi. Local Principal Investigator: Giulio Conte

Kantonsspital St. Gallen: Roman Brenner, David Altmann, Michaela Gemperle. Local Principal Investigator: Peter Ammann

Hôpital Cantonal Fribourg: Mathieu Firmann, Sandrine Foucras, Martine Rime. Local Principal Investigator: Daniel Hayoz

Luzerner Kantonsspital: Benjamin Berte, Kathrin Bühler, Virgina Justi, Frauke Kellner-Weldon, Melanie Koch, Brigitta Mehmann, Sonja Meier, Myriam Roth, Andrea Ruckli-Kaeppeli, Ian Russi, Kai Schmidt, Mabelle Young, Melanie Zbinden. Local Principal Investigator: Richard Kobza

Ente Ospedaliero Cantonale Lugano: Elia Rigamonti, Carlo Cereda, Alessandro Cianfoni, Maria Luisa De Perna, Jane Frangi-Kultalahti, Patrizia Assunta Mayer Melchiorre, Anica Pin,Tatiana Terrot, Luisa Vicari. Local Principal Investigator: Giorgio Moschovitis.

University Hospital Geneva: Georg Ehret, Hervé Gallet, Elise Guillermet, Francois Lazeyras, Karl-Olof Lovblad, Patrick Perret, Philippe Tavel, Cheryl Teres. Local Principal Investigator: Dipen Shah

University Hospital Lausanne: Nathalie Lauriers, Marie Méan, Sandrine Salzmann, Jürg Schläpfer. Local Principal Investigator: Alessandra Pia Porretta

Bürgerspital Solothurn: Andrea Grêt, Jan Novak, Sandra Vitelli. Local Principal Investigator: Frank-Peter Stephan

Ente Ospedaliero Cantonale Bellinzona: Jane Frangi-Kultalahti, Augusto Gallino, Luisa Vicari. Local Principal Investigator: Marcello Di Valentino

For peer review only

University of Zurich/University Hospital Zurich: Helena Aebersold, Fabienne Foster, Matthias Schwenkglenks.

Medical Image Analysis Center AG Basel: Jens Würfel (Head), Anna Altermatt, Michael Amann, Marco Düring, Petra Huber, Esther Ruberte, Tim Sinnecker, Vanessa Zuber.

Clinical Trial Unit Basel: Michael Coslovsky (Head), Pascal Benkert, Gilles Dutilh, Milica Markovic, Pia Neuschwander, Patrick Simon, Olivia Wunderlin

Schiller AG Baar: Ramun Schmid

For peer review only

STROBE Statement—Checklist of items that should be included in reports of ***cohort studies***

Estimating the cost impact of atrial fibrillation using a prospective cohort study and population-based controls

	Item No	Recommendation	addressed in the manuscript
**Title and abstract**	1	(*a*) Indicate the study's design with a commonly used term in the title or the abstract	p. 1
		(*b*) Provide in the abstract an informative and balanced summary of what was done and what was found	p. 3
**Introduction**			
Background/rationale	2	Explain the scientific background and rationale for the investigation being reported	p. 5
Objectives	3	State specific objectives, including any prespecified hypotheses	p. 5
**Methods**			
Study design	4	Present key elements of study design early in the paper	p. 6
Setting	5	Describe the setting, locations, and relevant dates, including periods of recruitment, exposure, follow-up, and data collection	p. 6
Participants	6	(*a*) Give the eligibility criteria, and the sources and methods of selection of participants. Describe methods of follow-up	p. 6, 7
		(*b*) For matched studies, give matching criteria and number of exposed and unexposed	NA
Variables	7	Clearly define all outcomes, exposures, predictors, potential confounders, and effect modifiers. Give diagnostic criteria, if applicable	p. 7
Data sources/ measurement	8*	For each variable of interest, give sources of data and details of methods of assessment (measurement). Describe comparability of assessment methods if there is more than one group	p. 6-8
Bias	9	Describe any efforts to address potential sources of bias	p. 6-8
Study size	10	Explain how the study size was arrived at	p. 6-7, Table S1-S2
Quantitative variables	11	Explain how quantitative variables were handled in the analyses. If applicable, describe which groupings were chosen and why	p. 7-9
Statistical methods	12	(*a*) Describe all statistical methods, including those used to control for confounding	p. 7-9
		(*b*) Describe any methods used to examine subgroups and interactions	p. 7-9
		(*c*) Explain how missing data were addressed	p. 7
		(*d*) If applicable, explain how loss to follow-up was addressed	p. 7
		(*e*) Describe any sensitivity analyses	p. 7-8
**Results**			
Participants	13*	(a) Report numbers of individuals at each stage of study—eg numbers potentially eligible, examined for eligibility, confirmed eligible, included in the study, completing follow-up, and analysed	p. 10, Table S1-S2
		(b) Give reasons for non-participation at each stage	p. 10
		(c) Consider use of a flow diagram	p. 10, Figure 1
Descriptive data	14*	(a) Give characteristics of study participants (eg demographic, clinical, social) and information on exposures and potential confounders	p.10, Table 1, Figure S1
		(b) Indicate number of participants with missing data for each variable of	NA

For peer review only - http://bmjopen.bmj.com/site/about/guidelines.xhtml

		interest	
		(c) Summarise follow-up time (eg, average and total amount)	Figure S1
Outcome data	15*	Report numbers of outcome events or summary measures over time	p. 10
Main results	16	(*a*) Give unadjusted estimates and, if applicable, confounder-adjusted estimates and their precision (eg, 95% confidence interval). Make clear which confounders were adjusted for and why they were included	p. 10, Tables S4-S7
		(*b*) Report category boundaries when continuous variables were categorized	NA
		(*c*) If relevant, consider translating estimates of relative risk into absolute risk for a meaningful time period	NA
Other analyses	17	Report other analyses done—eg analyses of subgroups and interactions, and sensitivity analyses	p. 10-11; Figures S3
**Discussion**			
Key results	18	Summarise key results with reference to study objectives	p. 11-13
Limitations	19	Discuss limitations of the study, taking into account sources of potential bias or imprecision. Discuss both direction and magnitude of any potential bias	p. 12-13
Interpretation	20	Give a cautious overall interpretation of results considering objectives, limitations, multiplicity of analyses, results from similar studies, and other relevant evidence	p. 11-13
Generalisability	21	Discuss the generalisability (external validity) of the study results	p. 11-13
**Other information**			
Funding	22	Give the source of funding and the role of the funders for the present study and, if applicable, for the original study on which the present article is based	p. 14

*Give information separately for exposed and unexposed groups.

Note: An Explanation and Elaboration article discusses each checklist item and gives methodological background and published examples of transparent reporting. The STROBE checklist is best used in conjunction with this article (freely available on the Web sites of PLoS Medicine at http://www.plosmedicine.org/, Annals of Internal Medicine at http://www.annals.org/, and Epidemiology at http://www.epidem.com/). Information on the STROBE Initiative is available at http://www.strobe-statement.org.

For peer review only
